# Methodologies for generating and evaluating clinical and performance evidence for high-risk and innovative medical devices and *in vitro* diagnostics: a scoping review

**DOI:** 10.3389/fmedt.2026.1857401

**Published:** 2026-06-24

**Authors:** Nensi Bralić, Ružica Bandić, Miro Vuković, Danira Matijaca, Renata Valsami, Panagiotis Zoumpoulakis, Kalle Schnitzer, Mari Levula, Risto Roine, Antti Viitala, Antti Pihlava, Olivia McDermott, Ana Marušić

**Affiliations:** 1 Department of Research in Biomedicine and Health, University of Split School of Medicine, Split, Croatia; 2 Central Medical Library, University of Split School of Medicine, Split, Croatia; 3 Greek Patients' Association, Athens, Greece; 4 Clinipower Finland Oy, Savonlinna, Finland; 5 SGS Fimko Ltd, Helsinki, Finland; 6 College of Science and Engineering, University of Galway, Galway, Ireland

**Keywords:** evidence-based medicine, *in vitro* diagnostic devices, medical devices, observational studies as topic, postmarketing surveillance, randomized controlled trials as topic, real-world evidence

## Abstract

**Introduction:**

High-risk and innovative medical devices (MDs) and *in vitro* diagnostic devices (IVDs) in the European Union are subject to increasingly stringent and evolving regulatory requirements. This scoping review maps methodological approaches to evidence generation across their life cycle.

**Methods:**

A comprehensive and systematic search of major bibliographic databases and grey literature sources was conducted (January 2025, updated November 2025). Studies were selected through dual independent screening using predefined inclusion criteria. Data were extracted using a structured approach and supported by AI-assisted thematic analysis. Findings were synthesized narratively to map methodological approaches to evidence generation and evaluation.

**Results:**

A total of 15,679 records were identified, with 14,259 remaining after duplicate removal and 859 full texts assessed for eligibility. Of these, 90 studies met the inclusion criteria. In addition, records identified through other methods were screened, with 75 progressing to full-text assessment; of these, 8 were included**.** Evidence generation for high-risk and innovative MDs and IVDs requires an integrated, life-cycle approach combining multiple study designs. While randomized controlled trials remain important, observational studies, real-world evidence, registries, and post-market surveillance are essential for assessing long-term safety and effectiveness. However, current practices show substantial variability, methodological limitations, and regulatory inconsistencies.

**Conclusions:**

An integrated lifecycle approach is needed for robust evaluation of high-risk and innovative MDs and IVDs. Strengthening methodological standards, improving the quality of real-world data, and enhancing regulatory harmonization are essential.

**Systematic Review Registration:**

Open Science Framework (OSF), doi: 10.17605/OSF.IO/XNBSP.

## 1 Introduction

High-risk and innovative medical devices (MDs) and *in vitro* diagnostic medical devices (IVDs) in the European Union (EU) are developed and assessed within a rapidly evolving regulatory landscape shaped by Regulation EU 2017/745 on medical devices (MDR) and Regulation EU 2017/746 on *in vitro* diagnostic medical devices (IVDR) (1, 2). These regulations have significantly strengthened evidentiary requirements and emphasize a continuous, life-cycle-based approach to evidence generation, extending from pre-market evaluation to post-market surveillance (PMS). Such an approach reflects the increasing complexity of these technologies and the need for robust, high-quality evidence to support regulatory and clinical decision-making (3, 4). Under the MDR, devices are classified by risk, with Class IIb covering medium- to high-risk products and Class III covering the highest-risk devices, which require the most stringent conformity assessment procedures and comprehensive clinical evaluation. For high-risk implantable devices, a total product life-cycle (TPLC) approach is advocated, encompassing preclinical testing, clinical studies, and post-market evidence generation through registries and real-world data (RWD) (5, 6). Similarly, the IVDR requires higher-risk IVDs (Classes C and D) to demonstrate both analytical and clinical performance, with increasing levels of evidence required as risk increases. For Class D devices, the most rigorous clinical evidence is expected, often supported by prospective or high-quality retrospective studies, and accompanied by enhanced post-market obligations (2, 7).

In addition, many innovative technologies, including artificial intelligence–based devices, may fall into higher-risk classes depending on their intended purpose and potential clinical impact (1, 5). For example, software intended to provide information for diagnostic or therapeutic decision-making may be classified into higher-risk classes when such decisions have a significant impact on patient health (1).

Traditional randomized controlled trials (RCTs), while important, are often insufficient to capture the complexity and real-world performance of MDs and IVDs (1, 2). In contrast to medicinal products, which typically follow a more standardized and sequential development pathway based on phased RCTs (e.g., Phase I–III) (8), the evaluation of MDs and IVDs increasingly relies on RWD, patient registries, and observational studies as complementary sources of evidence (9–11). However, incorporating such data into regulatory frameworks and decision-making requires rigorous methodological approaches that adequately account for bias, data integrity, and applicability (12). Additional challenges arise from operator dependency, learning curves, and rapid technological evolution, which distinguish device evaluation from pharmaceutical research (13, 14). Furthermore, variability in evidence requirements and assessment practices across EU member states adds another layer of complexity and underscores the need for greater alignment (15, 16).

Given these challenges, integrated and strategically planned approaches to evidence generation are increasingly essential, combining traditional and real-world evidence (RWE) within a coherent framework. This scoping review aims to identify, map, and evaluate the current evidence on methodological approaches used in the clinical investigation, performance assessment, and life-cycle evaluation of high-risk and innovative MDs and IVDs, to support the development of more consistent and robust methodologies for evidence-based decision-making in the EU. Although the review is primarily framed within the EU MDR/IVDR context, relevant international regulatory frameworks and methodological approaches were also considered to provide comparative perspectives and broader contextual insights**.**

## 2 Methods

This scoping review was conducted in accordance with the methodological guidelines for scoping reviews set out in the Joanna Briggs Institute Manual for Evidence Synthesis (17). The protocol was prospectively registered on the Open Science Framework (OSF) (18).

### 2.1 Search strategy and data sources

A detailed and comprehensive search strategy was developed to ensure methodological consistency and broad coverage of the literature. Key bibliographic databases, including PubMed, Scopus/Embase, and Web of Science, were systematically searched alongside specialized industry and professional resources such as MedTech Europe, JAMS, and FDB Prism. Additional sources included project and journal databases (the CORDIS database of EU-funded projects and journals focused on Health Technology Assessment) as well as preprint repositories such as PrePubMed, OSF Preprints, and search.bio Preprint. The search was conducted on 30 January 2025 in collaboration with an experienced librarian (DM), using a strategy that prioritized sensitivity over specificity, given the field's complex, variable terminology. Both peer-reviewed and grey literature were included, with peer-reviewed studies identified through bibliographic databases and grey literature through purposefully selected sources. Given the rapidly evolving nature of the field, the search was subsequently updated on 11 November 2025 to capture the most recent evidence**.** The full search strategies for all databases and sources are provided in Supplementary Appendix 1**.**

### 2.2 Inclusion and exclusion criteria

Original research papers, systematic reviews, theses, opinion articles, and relevant guidelines were included, without language restrictions. Only papers published on or after 5 April 2017, in line with the date of adoption of the EU MDR and IVDR, were considered.

#### 2.2.1 Inclusion criteria

The inclusion criteria encompassed the records that discussed how performance evidence for MDs and IVDs is generated and assessed, including approaches and methods used in performance evaluation. Studies addressing methodological aspects of different study designs were included, particularly those analysing the appropriateness, advantages, disadvantages, or other methodological considerations of designs such as RCTs and non-RCTs, uncontrolled clinical trials, cohort, case-control, and cross-sectional studies, as well as case series and case reports. In addition, sources examining RWD and RWE, registries, and master protocols (including platform, basket, and umbrella trials) were considered, with emphasis not only on their application but specifically on their role in the performance evaluation of MDs/IVDs. Records analyzing the use of RWD and registries in regulatory decision-making, performance evaluation, and evidence development were also included, as were reviews of PMS methodologies and data structures, particularly those focused on monitoring performance after-market placement.

#### 2.2.2 Exclusion criteria

The exclusion criteria comprised records not related to MDs or IVDs, including studies focused on medicinal products, general clinical care, or other technologies, as well as those that only briefly mention MDs/IVDs without substantive analysis. Documents that merely describe existing regulatory frameworks without providing methodological insight or interpretation were also excluded.

### 2.3 Study selection

All identified search sources were imported into Rayyan, a web-based systematic review tool (19). Screening was conducted in two stages: first, titles and abstracts were independently reviewed; then, potentially relevant records were analyzed in full text. A high number of records for full-text screening reflects the limited information available at the title and abstract stage, where many records lacked structured abstracts, represented non-original publications (e.g., commentaries, reviews, or regulatory documents), or did not provide sufficient methodological detail to allow confident exclusion. Therefore, a deliberately inclusive approach was adopted to avoid prematurely excluding potentially relevant studies.

Twelve reviewers (AM, NB, RB, MV, RV, PZ, ML, RR, AV, AP, KS, OM) worked in pairs and independently selected studies. Both reviewers had to agree on inclusion. Disagreements were discussed and, if needed, mediated by lead researchers (AM, NB, RB, MV).

### 2.4 Data extraction

A structured data extraction process was implemented using a standardized spreadsheet, with each included study assigned a unique identification number. Bibliographic and methodological information was collected for all studies, including title, first author, year of publication, time span of included studies (for secondary research), and study design. The extraction was conducted in two phases. In Phase 1 (study categorization), four reviewers (AM, NB, RB, MV) worked in pairs to classify studies according to predefined thematic categories: study designs, use of real-world evidence, use of registries, post-market surveillance, life-cycle assessment, and other topics. Although registries are commonly considered a source of real-world evidence and post-market surveillance activities form part of a broader life-cycle approach, these categories were analysed separately to enable a more granular assessment of their specific methodological and regulatory roles. Studies could be assigned to multiple categories, and reviewers assessed whether each topic was covered in sufficient detail to proceed with further extraction. In Phase 2 [detailed topic extraction with artificial intelligence (AI)], an AI-assisted approach was used to manage the large volume and complexity of data. The large language model GPT-4o was applied via the ChatGPT Plus platform to support text mining and structured extraction (20). The methodological approach included three steps: (1) prompt formulation, where prompts were developed and tested on a sample of 10 studies or categories, initially generated using ChatGPT and subsequently refined to ensure accuracy and consistency; this iterative testing process involved comparing AI-generated outputs across sample studies and refining prompts to improve clarity, relevance, and consistency of extracted information. The final prompts used for extraction and synthesis are provided in Supplementary Appendix; (2) topic extraction, in which GPT-4o identified key elements for each study and category, including methodological overview, regulatory considerations, limitations and challenges, and variability and gaps in literature; AI-generated outputs were reviewed independently by the authors to verify factual accuracy, contextual appropriateness, and alignment with the original source material. Any inconsistencies, uncertainties, or ambiguous interpretations identified during review were resolved through discussion and consensus among the authors, with direct re-examination of the original documents where necessary; and (3) mapping, where extracted data were summarized and organized by category to produce a structured synthesis of the evidence. Final category-level summaries were developed by grouping findings across studies and evidence types to identify recurring themes, methodological patterns, and regulatory considerations. Original study records and extracted source materials were provided to GPT-4o to support analysis and evidence synthesis. All AI-generated outputs were subsequently reviewed, summarized, verified, and refined by the authors to ensure factual accuracy, contextual appropriateness, and alignment with the original documents.

### 2.5 Data analysis and presentation

The results are presented in narrative form, in accordance with PRISMA-ScR guidelines (17). As this was a scoping review, a risk-of-bias assessment was not performed (21). A structured overview of the available evidence was prepared, focusing on the primary methods for generating and evaluating clinical and performance data. The synthesis combined deductive and inductive approaches to identify relevant concepts, patterns, and variations in the literature.

## 3 Results

### 3.1 Study identification and analytical structure

**Figure 1:**
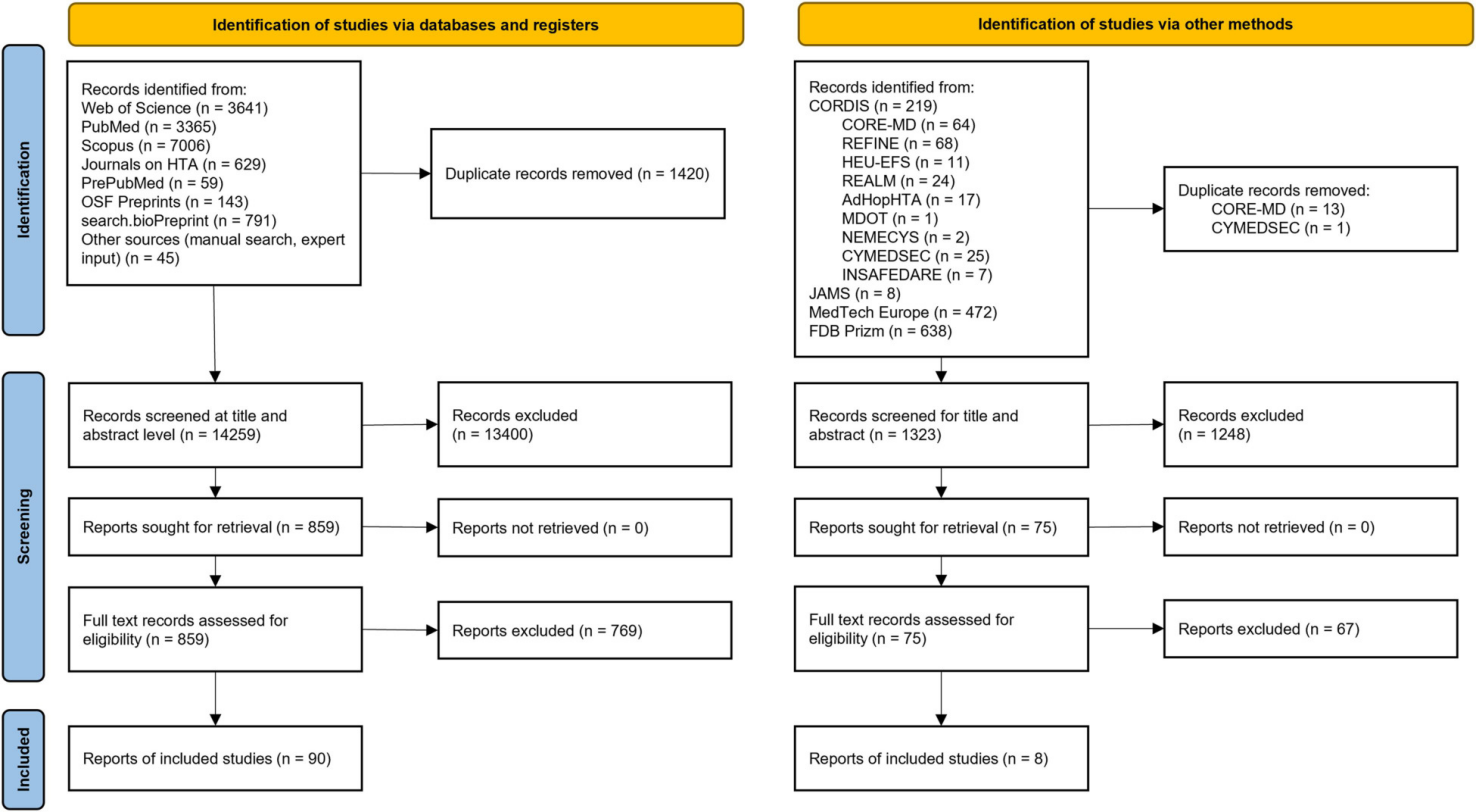
PRISMA flowchart.

The summary of the record identification, screening, and inclusion process is presented in the PRISMA flow diagram (Figure 1). The database search identified 15,679 records; after removing duplicates, 14,259 remained for screening. Following title and abstract review, 859 full texts were assessed. An additional 75 records identified through other methods proceeded to full-text assessment, of which 8 were included in the review. In total, 98 studies were included in the final review (5, 7, 22–117) (Supplementary Table 1).

All included studies were published between 2017 and 2025, following the introduction of the EU MDR and EU IVDR regulations. The records were categorized by document type and purpose. Non-empirical literature (*n* = 30) included commentaries, expert opinions, opinion papers, and perspective articles. Evidence syntheses (*n* = 31) comprised systematic reviews, scoping reviews, narrative reviews, and other structured approaches to summarizing existing evidence. Original research articles (*n* = 19) comprised primary studies that involved original data collection and analysis, using both quantitative and qualitative methodologies. Project-based documents (*n* = 15) included outputs of research and innovation initiatives, such as deliverables, white papers, and technical or methodological reports. Additionally, two book chapters (*n* = 2) published in edited volumes and one doctoral dissertation (*n* = 1), representing a PhD thesis relevant to the methodological focus of the review, were included. Given the broad scope of the review, diverse evidence types were included to capture methodological, regulatory, and implementation perspectives relevant to evidence generation for high-risk and innovative MDs and IVDs. All included sources were analyzed using a common structured data-charting and narrative synthesis approach. No formal weighting or hierarchical ranking of evidence types was applied, consistent with the scoping review methodology. Different source types contributed to the synthesis according to their content and purpose; empirical studies and evidence syntheses primarily informed methodological and evidentiary aspects, whereas non-empirical and project-based documents contributed contextual, conceptual, and regulatory perspectives.

**Figure 2:**
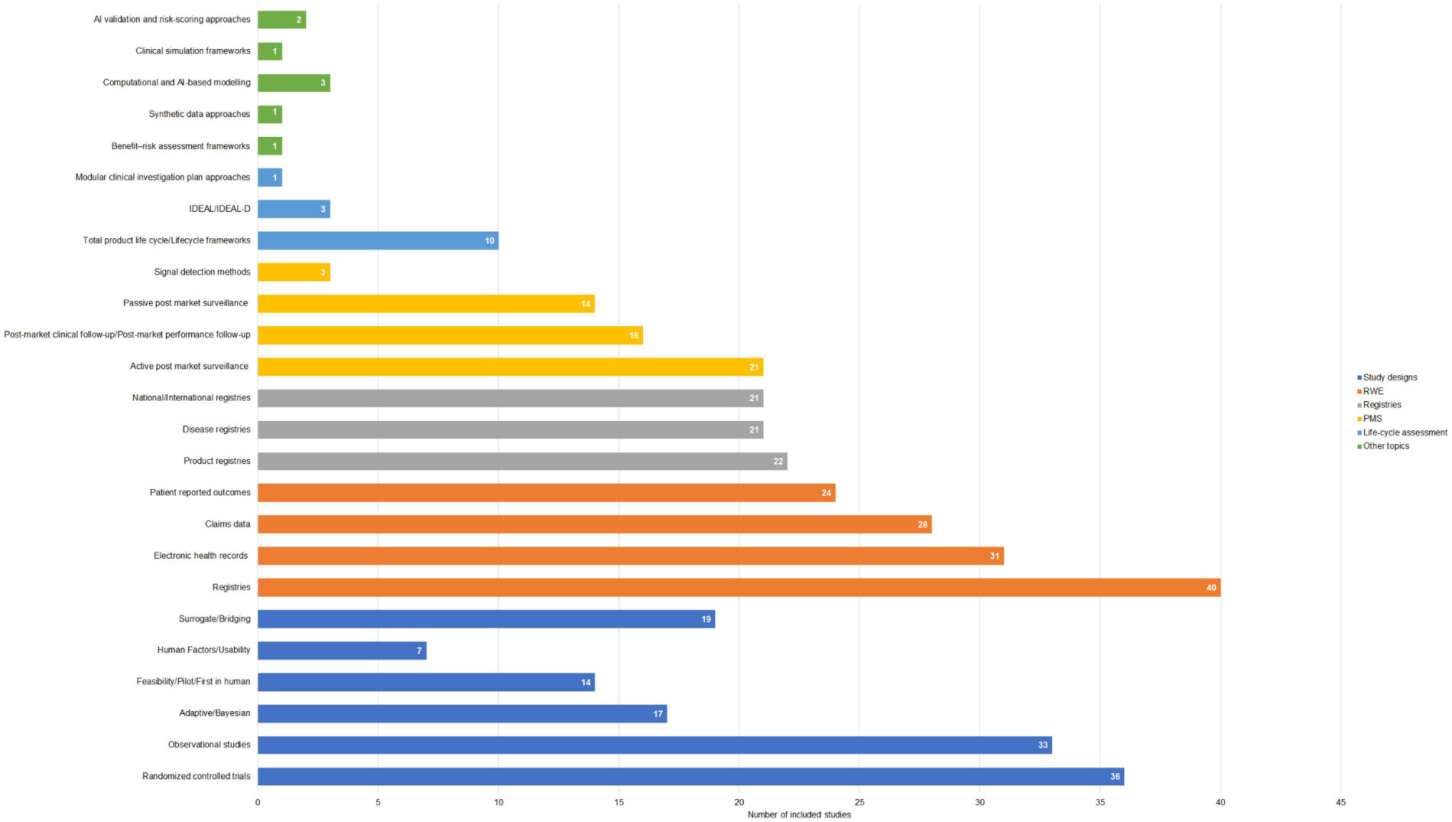
Distribution of included studies across thematic categories and subcategories related to evidence generation for high-risk and innovative medical devices and IVDs. Categories were analysed separately to enable a more granular evaluation of their specific methodological and regulatory roles; therefore, registries were examined independently despite being commonly considered a source of real-world evidence (RWE), and post-market surveillance (PMS) activities were analysed separately from broader life-cycle assessment frameworks. Because some studies addressed multiple topics, individual records could be assigned to more than one category. AI – artificial intelligence, IDEAL(-D) – idea, development, exploration, assessment, and long term study (-device).

 Figure 2 summarizes the thematic categorization of the included studies and demonstrates the broad distribution of methodological, regulatory, and evidence-generation topics identified across the literature, with some studies contributing to multiple categories.

### 3.2 Study designs

Various study designs are used to generate and assess clinical and performance evidence for high-risk and innovative MDs and IVDs, with the choice depending on the device life-cycle stage, regulatory context, characteristics, and feasibility.

#### 3.2.1 Overview of methods

RCTs are widely regarded as the gold standard for proving safety, efficacy, and comparative effectiveness because they reduce bias and enable causal conclusions. They are most often used in pivotal confirmatory trials, especially for mature, high-risk, or innovative devices, although their use in the MD/IVD field is frequently constrained by ethical, logistical, and practical challenges, such as small patient groups, ongoing device modifications, and operator-dependent procedures (24, 29, 31, 32, 36, 39, 41, 43, 46–48, 50, 51, 55, 57, 60–62, 65, 66, 68, 72, 74, 76, 80, 82, 89, 98–100, 102, 107, 108, 113, 114, 117). Consequently, the literature describes frequent reliance on alternative and complementary study designs when RCTs are not feasible (109, 113, 114). Study design selection should reflect device risk, intended use, and regulatory purpose, with clinical evidence proportional to the risk classification and study designs that minimize bias, ensure applicability, and provide adequate precision (111, 114, 117). In-silico clinical trials (ISCTs), which use computational modeling and simulation to evaluate device performance, safety, and efficacy through virtual patient populations, were discussed as an emerging or complementary study design, particularly for innovative or high-risk devices and in contexts where traditional clinical trials are constrained (111).

Non-randomized and observational approaches, such as prospective and retrospective cohort studies, case-control studies, single-arm trials, and registry-based designs, are commonly used when RCTs are not practical. They are widely applied in both pre-market and post-market settings and are often supported by advanced statistical methods, including propensity score matching and inverse probability weighting, to help reduce confounding. A key strength of these designs is their strong real-world generalisability and their ability to capture long-term outcomes (23, 24, 29, 31, 32, 39, 42, 44, 46–48, 50, 57, 60–63, 65, 66, 68, 72, 74, 78, 80, 82, 95, 98, 100, 102, 107, 112, 114, 117).

Adaptive and Bayesian designs, both subtypes of RCTs, were prominent in the reviewed literature. Adaptive designs allow protocol changes based on interim results to improve efficiency and reduce resource use. Bayesian approaches incorporate prior information and enable flexible adjustment of sample size, which is particularly useful for rapidly evolving technologies or rare conditions. Both are increasingly accepted by regulators due to their flexibility and fit with real-world development constraints (31, 32, 34–36, 43, 47, 48, 50, 55, 60, 61, 67, 76, 98, 99, 104).

Early feasibility, pilot, and exploratory studies, including first-in-human designs, play a key role in early development. These small, flexible studies enable initial assessment of safety, performance, and usability while supporting iterative refinement, and they are increasingly recognized by regulators, especially for novel and high-risk technologies (31, 40, 46, 47, 64, 81, 87, 89, 90, 92, 98, 99, 108, 114).

Surrogate endpoints and bridging studies are used when direct clinical outcomes are difficult to measure or when transitioning between device versions. Although they improve efficiency, their acceptance depends on strong statistical validation and clear evidence that surrogate markers reliably reflect meaningful clinical outcomes (25, 31, 32, 36, 41, 47, 50, 60, 62, 66–68, 71, 79, 88, 94, 99, 113, 114).

Usability and human factors studies were particularly highlighted for software-based devices or those requiring substantial user input. They assess interactions between users and devices to identify use-related risks and support safety claims. Both formative and summative studies were reported, conducted in either simulated settings or real clinical environments (40, 50, 79, 95, 98, 104, 112).

Different study designs contribute to varying levels of evidence, ranging from analytical and clinical validation to assessment of clinical utility, depending on the device’s intended use and risk (112, 117).

PMS and RWE studies, such as post-market clinical follow-up (PMCF) and registry-based tracking, were highlighted as essential for generating continuous evidence. These approaches enable long-term monitoring of safety, the identification of uncommon adverse events, and the ongoing evaluation of the benefit-risk balance. Their role is particularly important for high-risk and pediatric devices, although limitations in data completeness, reliability, and methodological clarity persist (24, 29, 31, 32, 34, 40, 42, 46–48, 50, 57, 60–63, 65, 68, 71, 72, 74–78, 80–82, 86, 88, 89, 95, 98–100, 104, 107, 108, 114, 117).

#### 3.2.2 Regulatory considerations

Regulatory frameworks consistently underline the need to select study designs that are fit for purpose, taking into account device risk, intended use, and existing evidence. Study designs play different roles in the pre-market and post-market stages of device evaluation. In the pre-market phase, designs such as RCTs and feasibility studies are used to produce initial and confirmatory evidence for regulatory approval (24, 31, 32, 34–36, 39–43, 46–48, 50, 51, 55, 57, 60, 62–66, 68, 71, 74, 76, 77, 80–82, 87–90, 92, 95, 98–100, 102, 104, 107, 108, 112–114). Although RCTs remain the gold standard for pivotal evidence, regulators permit alternative study designs when there is sound justification (24, 25, 29, 31, 32, 34–36, 39–44, 46–48, 50, 51, 55, 57, 60–66, 68, 71, 74–82, 86–90, 92, 95, 98, 99, 102, 104, 107, 108, 111–113, 117). For high-risk devices, regulators may expect prospective pivotal studies supported by complementary evidence sources (111, 112).

Bridging and equivalence studies, together with surrogate endpoint validation and literature reviews, help substantiate claims when moving between device versions or when establishing comparability (24, 60, 67, 71, 79, 82, 88, 94, 95).

In the post-market setting, PMCF, RWE, and registry studies are used to meet regulatory requirements for ongoing monitoring and periodic reassessment (23, 24, 29, 31, 32, 42, 46–48, 57, 61, 65, 74, 76–78, 81, 82, 98–100, 102, 104, 107, 108, 113, 114, 117).

However, important gaps remain, particularly the absence of harmonized guidance on how to incorporate human factors, RWE, and software- or AI-specific methods into regulatory submissions (65, 104, 111).

#### 3.2.3 Limitations and challenges

A key challenge is that RCTs are sometimes impractical due to ethical, logistical, or real-world constraints, especially in settings with small populations or rapidly evolving innovations (24, 25, 29, 31, 32, 36, 41, 43, 46–48, 50, 51, 57, 60, 61, 63, 65, 66, 68, 74, 76, 82, 89, 98–100, 102, 104, 107, 108, 113). Non-randomized studies may still be subject to bias even after statistical adjustments (23, 29, 32, 39, 41, 42, 44, 46–48, 57, 60–63, 68, 72, 74, 75, 78, 80, 82, 89, 95, 102, 108, 113, 117), and adaptive or Bayesian designs can be difficult to plan and execute statistically (32, 34–36, 43, 47, 48, 50, 55, 92, 99, 104). Early feasibility studies are restricted by small sample sizes and limited generalizability (46, 47, 64, 87, 92, 99, 108, 113), while usability studies often require substantial resources (40, 79, 95, 104), and bridging or surrogate studies rely heavily on strong underlying assumptions (31, 47, 60, 62, 67, 68, 71, 82, 88, 94, 99). RWE studies also continue to struggle with issues such as data quality, incomplete information, and reduced reliability when appropriate comparators are lacking (24, 29, 31, 32, 42, 46–48, 65, 71, 76, 78, 82, 86, 100, 102, 107, 108).

#### 3.2.4 Variability and gaps in literature

Several areas of inconsistency were identified. Unclear and overlapping terminology for study types (such as “pilot,” “feasibility,” and “exploratory”) makes it difficult to classify study designs consistently (64, 108).

Differences between jurisdictions persist, with U.S. guidance typically being more structured than EU documents, resulting in variation in study rigor and reporting practices (31, 34, 40, 48, 51, 60–62, 64, 87, 90).

There is no unified guidance on how to incorporate usability, human factors, and RWE into regulatory submissions, particularly for AI-driven or software-based devices (34, 40, 95, 98).

Approaches to surrogate and composite endpoints are inconsistent, with differing criteria for their acceptance (31, 34, 36, 50, 60, 62, 67, 68, 82, 99).

Generating post-market evidence remains challenging due to issues with data quality, standardization, and the integration of RWE into regulatory decision-making (24, 29, 32, 34, 42, 44, 46–48, 50, 57, 60, 61, 63, 65, 71, 74, 75, 77, 78, 80–82, 86, 98–100, 102, 104, 107, 113).

There is also a shortage of device-specific methodological guidance, especially for paediatric, AI, software, and companion diagnostic technologies (34, 39, 41, 43, 55, 57, 64–66, 71, 72, 74–77, 79, 81, 86, 88, 89, 94, 95, 102, 104, 112).

**Figure 3:**
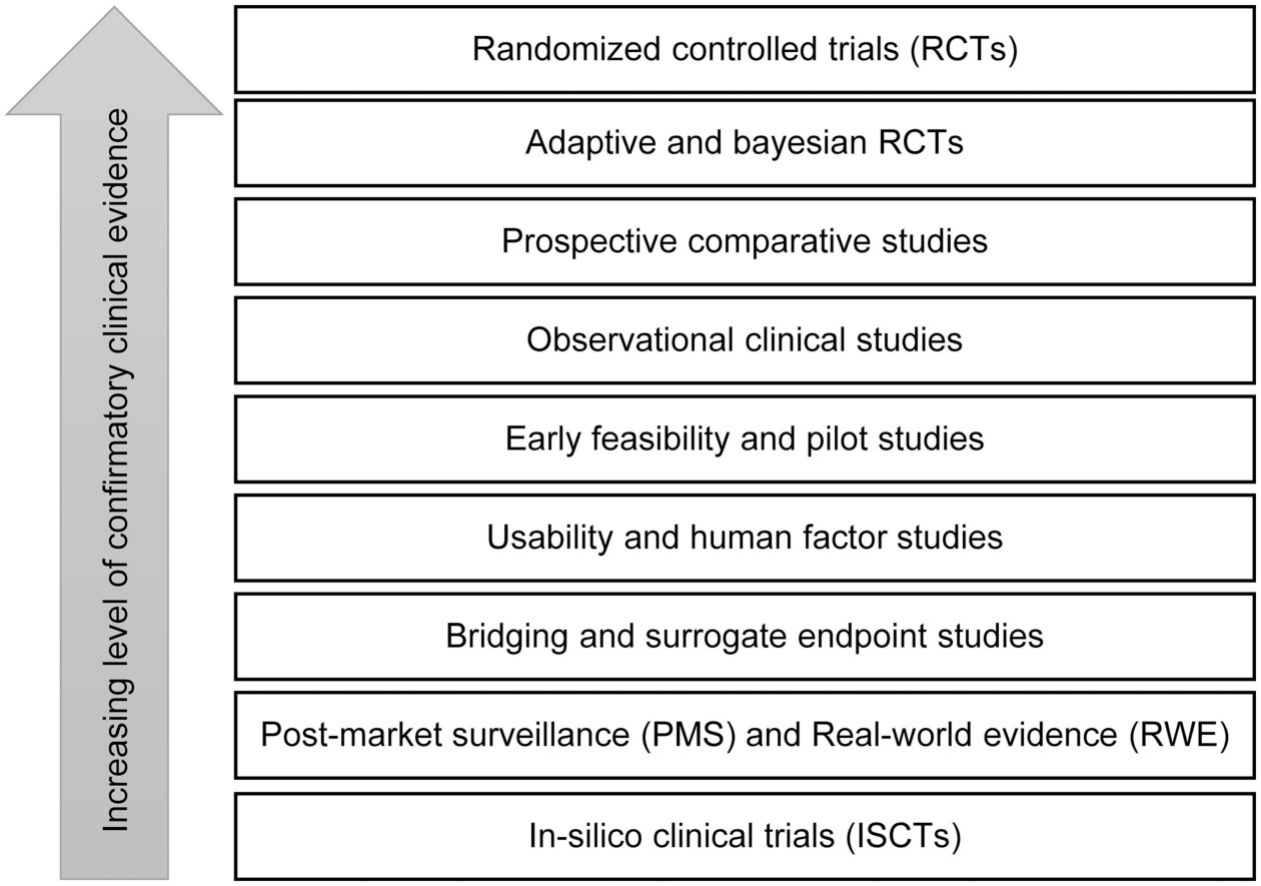
Conceptual continuum of evidence-generation approaches for high-risk medical devices. The upward direction indicates increasing levels of confirmatory clinical evidence and should be interpreted as a complementary framework rather than a strict evidence hierarchy.

Overall, as illustrated in Figure 3, evidence generation for high-risk medical devices extends beyond conventional clinical trials and increasingly relies on integrated frameworks combining traditional and emerging approaches across different levels of evidence throughout the device lifecycle.

### 3.3 Use of real-world evidence

RWE is increasingly seen as a crucial element in generating and assessing clinical and performance data for high-risk and innovative MDs and IVDs. The studies reviewed consistently highlighted RWE as an important complement to traditional RCTs and, in some cases, a practical alternative, particularly where RCTs are not feasible.

#### 3.3.1 Overview of methods

The reviewed studies reported a broad range of RWD sources, spanning established patient and device registries, electronic health records (EHRs), administrative claims data, patient-reported outcomes (PROs), and digital health platforms.

Patient and device registries were frequently highlighted as the most structured and reliable sources of longitudinal information, especially for implantable or high-risk devices (26–28, 31, 33, 34, 42, 45, 47, 52–54, 56, 57, 59, 61, 63, 65, 69, 70, 73, 81–85, 96, 97, 103, 106, 109, 114, 116).

EHRs provided rich clinical histories and ongoing outcome monitoring, enabling pragmatic and registry-based studies (26–29, 34, 42, 47, 48, 54, 56, 59, 61, 63, 65, 69, 70, 73, 78, 83–85, 96, 97, 101, 103–106, 109, 114, 116).

Claims and billing datasets offered population-level insights into healthcare use and safety, and were commonly applied in PMS (26–29, 34, 48, 53, 54, 56, 59, 61, 63, 65, 70, 73, 82–85, 96, 97, 101, 103–106, 109, 116).

PROs provide a patient-centred perspective on device performance and quality of life and are increasingly included in regulatory evidence (26, 29, 31, 42, 45, 47, 48, 53, 54, 63, 65, 69, 70, 73, 78, 84, 85, 96, 97, 103, 106, 112, 114, 116).

Other data sources mentioned included mobile technologies, wearable devices, digital health platforms, laboratory information systems, administrative databases, spontaneous adverse event reports, and published literature (7, 27–29, 34, 42, 53, 54, 56–59, 63, 69, 70, 73, 83–86, 96, 97, 101, 103–105, 109). Together, these diverse data types supported the generation of evidence on safety, effectiveness, long-term performance, and user experience. RWE research designs encompassed both retrospective and prospective observational studies, registry-based trials, and hybrid approaches that integrate RWD into Bayesian or pragmatic trial frameworks.

#### 3.3.2 Regulatory considerations

Across the reviewed literature, the degree to which regulators accept RWD varies widely by region, largely due to differences in the development and level of detail of their guidance frameworks.

Within the EU, the MDR and IVDR acknowledge RWE as a complement and, in some cases, an alternative to traditional clinical evidence, especially for high-risk or novel devices and in PMCF. However, EU guidance tends to be less detailed and is applied inconsistently across member states. Differences in methodological expectations have been identified as a barrier to the consistent inclusion of RWE in regulatory submissions, prompting calls for clearer, more comprehensive EU-level guidance (7, 31, 45, 47–49, 53, 56, 57, 59, 65, 69, 78, 81–84, 103, 104, 116). In the United States, the FDA has taken an active, supportive approach to incorporating RWE into regulatory decision-making. Multiple guidance documents released in 2017, 2018, 2021, and 2023 specify the circumstances in which RWE may be used in both pre-market and post-market settings. RWE can support initial approvals, label expansions, and post-market surveillance, provided the data are fit for purpose, relevant, and reliable. The FDA also promotes the use of RWD in innovative study designs, such as Bayesian, registry-based, and pragmatic trials (26–28, 33, 34, 45, 48, 49, 53, 54, 58, 59, 61, 63, 69, 70, 73, 82, 83, 85, 96, 97, 101, 103–106, 109, 112, 116).

Beyond the U.S. and EU, other regions are also progressing in their use of RWE. In China, the National Medical Products Administration (NMPA), together with pilot regulatory zones such as Boao Lecheng, explicitly supports RWE in applications for imported and innovative medical devices. These initiatives are supported by structured frameworks that ensure data quality and enable multidisciplinary oversight (27, 33, 42, 49, 52, 59, 96).

Despite increasing momentum, several common requirements and limitations remain across regulatory systems. Acceptance of RWE generally depends on demonstrating strong methodological rigor, well-defined protocols, and transparency in data management and analysis (7, 26, 27, 29, 34, 42, 45, 47–49, 52–54, 56–59, 61, 63, 65, 69, 70, 73, 78, 82–86, 96, 97, 101, 104–106, 109, 114, 116).

RWE has been shown to support several regulatory functions throughout the medical device life cycle. One key role is to demonstrate safety, performance, and effectiveness, particularly in real-world clinical conditions. Research highlights its usefulness for tracking long-term outcomes, identifying rare adverse events, and evaluating device performance outside controlled trial settings. This is especially important for high-risk or innovative devices, where traditional clinical trials may not fully reflect performance in routine practice (26–29, 31, 33, 34, 42, 45, 47, 52–54, 56–59, 61, 65, 69, 70, 73, 78, 81–86, 96, 97, 101, 103–106, 109, 112, 114, 116).

RWE is also increasingly important for regulatory submissions and compliance. It is used in both pre-market and post-market contexts to support label expansions, meet post-approval evidence requirements, and support PMCF activities under the EU MDR and IVDR frameworks (7, 27, 28, 31, 33, 34, 42, 45, 47, 49, 53, 54, 56–59, 61, 63, 65, 69, 70, 73, 81–86, 96, 97, 101, 103–106, 109, 112, 114, 116).

In certain situations, RWE can also act as an alternative to RCTs. When RCTs are impractical or unethical, RWE may partially or fully replace them, provided that strong methodological standards and transparency are ensured (7, 26, 28, 29, 31, 33, 34, 42, 45, 47–49, 52–54, 56–59, 61, 63, 65, 69, 70, 73, 78, 82–86, 96, 97, 101, 103–106, 109, 114, 116).

The literature further identifies several specific regulatory applications of RWE. These include its use as an external control arm in single-arm studies, integration into Bayesian adaptive trial designs, and application in pragmatic and registry-based RCTs. Additionally, RWE can help bridge evidence across different regions or populations, supporting international regulatory approvals and facilitating market access (26, 29, 34, 48, 52, 61, 63, 65, 70, 82–85, 96, 97, 104, 105, 114).

#### 3.3.3 Limitations and challenges

Concerns about data quality and completeness are central challenges in using RWE to assess MDs. Problems such as missing information, underreporting, inconsistent coding practices, lack of standardized definitions, and incomplete follow-up can undermine the reliability of RWE findings (26–29, 31, 33, 34, 42, 45, 47, 48, 52–54, 56–59, 61, 63, 65, 69, 70, 73, 78, 81–86, 96, 97, 101, 103–106, 109, 114, 116).

Methodological difficulties were also widely recognized. Bias and confounding, including unmeasured confounding, particularly selection and information bias, threaten internal validity. Although sophisticated statistical approaches, such as propensity score methods, g-methods, and Bayesian techniques, are increasingly applied to mitigate these issues, some residual bias often persists, especially in non-randomized studies (7, 26, 27, 29, 31, 33, 34, 42, 45, 47, 48, 52, 54, 56, 57, 59, 61, 63, 65, 69, 70, 78, 81–86, 96, 101, 104–106, 109, 114, 116).

Another limitation concerns the broader applicability of the results. Differences in healthcare systems, clinical practices, and patient pathways across regions mean that RWE findings may not always be transferable beyond the specific context in which they were generated (26, 42, 48, 52, 54, 69, 82, 83, 85, 86, 109, 116).

Finally, operational and infrastructure-related barriers were identified. These include limited data interoperability, a lack of harmonized standards, and constraints linked to data privacy regulations. In addition, many organizations lack adequate analytical infrastructure or specialized expertise to manage and interpret complex RWD, further restricting its routine use in regulatory decision-making (26–29, 31, 33, 45, 48, 53, 54, 58, 59, 61, 63, 65, 69, 70, 73, 78, 83–85, 96, 97, 101, 103–106, 109, 116).

#### 3.3.4 Variability and gaps in literature

Beyond the previously identified regulatory, methodological, and technical limitations, the literature pointed to uneven evidence across device categories. While implantable and other high-risk devices are well represented, the available evidence on the current and potential use of RWE remains uneven across device types and IVD subtypes. This imbalance in the evidence base may contribute to variability in how RWE is applied and evaluated across regulatory contexts, particularly for high-risk and innovative devices, highlighting a notable research gap (65, 70, 73, 81, 82, 86).

**Figure 4:**
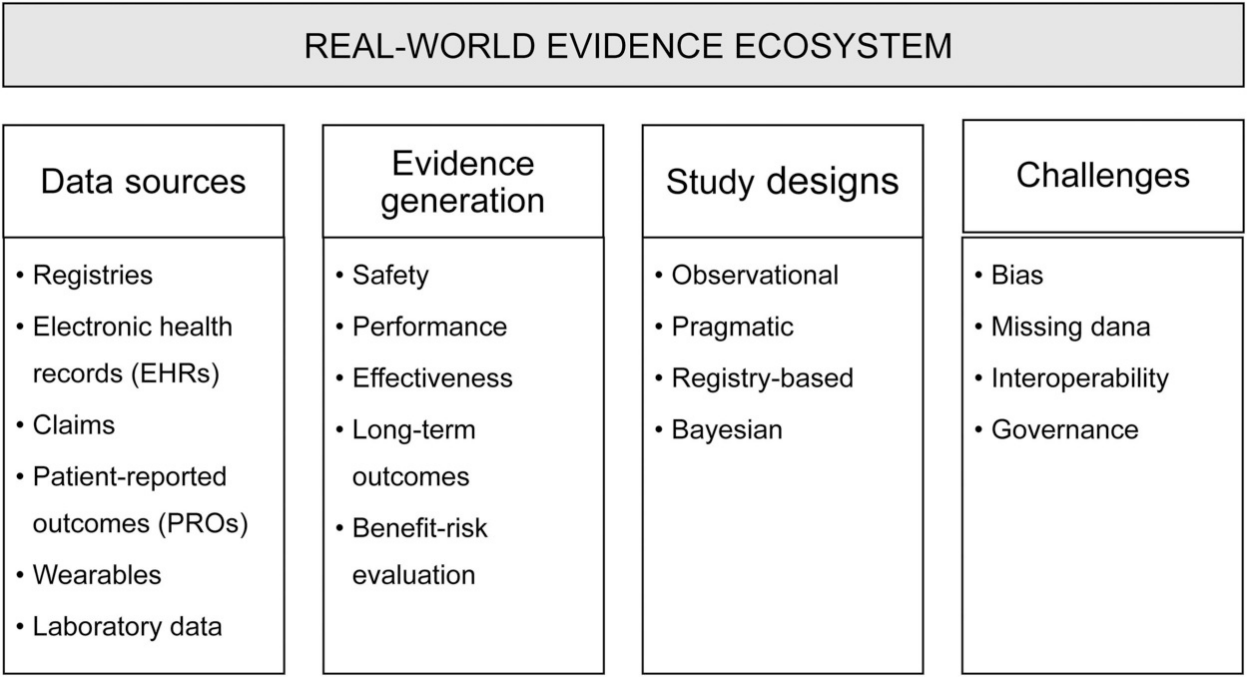
Conceptual overview of the real-world evidence ecosystem for high-risk and innovative medical devices and IVDs, highlighting key data sources, including registries, evidence-generation domains, study designs, and associated challenges.

Figure 4 provides an overview of the real-world evidence ecosystem, demonstrating how different data sources, study designs, and methodological considerations contribute to evidence generation across the device lifecycle.

### 3.4 Use of registries

The literature consistently highlights registries as a key structured source of RWD. Such data sources are widely used to support the generation and evaluation of clinical and performance evidence for high-risk and innovative MDs and IVDs.

#### 3.4.1 Overview of methods

The reviewed literature identified several categories of registries, each contributing in different ways to the generation of clinical and performance evidence.

Product-specific registries are designed to systematically collect outcome data related to specific medical technologies. These registries are frequently used for high-risk or innovative devices, including cardiovascular implants, orthopaedic implants, and certain *in vitro* diagnostic tests developed for rare conditions. In many publications, they are described as particularly valuable within PMS activities and studies supporting label expansions, as they provide structured real-world information on device utilization, patient exposure, and follow-up outcomes (7, 27, 28, 31–34, 42, 45, 47, 48, 53, 56, 61–63, 69, 70, 73, 97, 103, 114).

Disease-specific registries collect data from individuals diagnosed with a particular medical condition. They are especially useful in situations where RCTs are difficult to conduct, for example, in rare diseases or paediatric populations where patient numbers are limited. Such registries can contribute to evidence generation both before market authorization and during the post-market phase (7, 27, 28, 32, 33, 45, 47, 48, 56, 57, 60–63, 69, 70, 73, 76, 97, 103, 114).

Another category includes large national or international registries, which are typically coordinated by governmental institutions or professional medical societies. Due to their broad population coverage, these registries enable population-level surveillance and are particularly valuable for evaluating widely used or well-established technologies. They also support comparative effectiveness analyses across different devices or treatment approaches (27, 28, 31–33, 45–48, 53, 57, 60, 61, 63, 69, 70, 73, 76, 97, 103, 114).

Registries that focus on specific medical procedures or healthcare services represent another important data source. Although they are less often used as the sole basis for generating evidence, they are frequently combined with other registry types to improve data completeness and strengthen the evidence submitted for regulatory purposes (73, 97).

As shown in Figure 4, registries represent one of the major data sources within the broader real-world evidence ecosystem; however, they were considered separately in this review to enable a more detailed evaluation of their specific methodological and regulatory contributions.

#### 3.4.2 Regulatory considerations

In the pre-market setting, registries can complement evidence generated through traditional clinical trials, particularly for novel or high-risk MDs where pre-market clinical evidence may be limited. When conducting RCTs is impractical or infeasible, registry data may be used to establish historical control groups, support the expansion of device indications, or serve as external comparators in non-randomized studies (7, 28, 32, 34, 42, 47, 48, 57, 60, 62, 63, 69, 70, 73, 97, 103, 114). Their importance becomes even greater during PMS. Registries are frequently used to track long-term safety and effectiveness, identify potential adverse events, and evaluate the ongoing benefit–risk balance of medical devices. They also contribute to PMCF activities required under the EU MDR and provide input for periodic safety update reports (PSUR) and clinical evaluation reports (CER) (7, 27, 28, 31–34, 42, 45–48, 53, 56, 57, 60, 61, 69, 70, 73, 76, 97, 103, 114).

Within the framework of the EU MDR and IVDR, registries are identified as potential data sources in post-market follow-up activities, as reflected in Annex XIV, Part B, Section 6.2 (b) of EU MDR (1) and Annex XIII, Part B, Section 5.2 (b) of EU IVDR (2). Despite this recognition, registries are presented as one of several possible data sources, and their use is not explicitly mandated (1, 2). Their acceptance by NBs is therefore determined on a case-by-case basis and largely depends on the robustness of the data and the transparency of the underlying methodology (7, 27, 31, 45–47, 53, 56, 57, 60, 103).

Beyond the EU, several regulatory authorities and international organizations have also issued guidance on how registries can support regulatory decisions. Agencies such as the FDA and the International Medical Device Regulators Forum (IMDRF) have outlined fundamental criteria that registry-derived data must meet to be considered suitable for regulatory purposes. These criteria typically include ensuring that the data are relevant to the regulatory question, reliable, scientifically valid, and aligned with regulatory objectives (27, 28, 31–34, 42, 45, 46, 48, 57, 61–63, 70, 73, 103, 114).

Moreover, specific evaluation frameworks have been developed to assess whether registries are appropriate for use in regulatory contexts. For instance, initiatives such as the Clinical Trials Transformation Initiative (CTTI) and the Medical Device Innovation Consortium (MDIC) have proposed structured approaches that define criteria for evaluating elements such as data quality, governance mechanisms, and the validity of analytical methods (34, 42, 45, 48, 61, 63, 69, 70, 73, 103, 114).

The literature increasingly highlights the importance of interoperability. There is growing interest in integrating registry data with other healthcare data sources, including EHRs, insurance claims databases, and biobanks. Such data linkages can support more comprehensive evidence generation and strengthen the potential for regulatory acceptance (7, 27, 28, 31–33, 42, 45, 47, 48, 53, 56, 60, 61, 69, 70, 73, 97, 103, 114).

In addition, registries contribute to health technology assessment (HTA) processes by generating RWE on device performance after market entry, patient health outcomes, and economic value (31, 53, 60, 69, 114).

#### 3.4.3 Limitations and challenges

One of the most frequently discussed challenges related to the use of registries concerns the reliability and completeness of the collected data. Many registries face difficulties, such as missing information, irregular patient follow-up, or inconsistent outcome definitions, which can weaken the strength and credibility of the resulting evidence (7, 27, 28, 31–34, 42, 45–48, 53, 56, 57, 60–63, 69, 70, 73, 76, 97, 103, 114).

Closely related to this issue are challenges associated with data standardization and interoperability. Differences in data structures, coding practices, and data collection procedures across registries make it difficult to integrate datasets or conduct cross-registry comparisons. Although initiatives promoting harmonization, such as adopting common data models and using UDIs, have been proposed, their implementation remains uneven (7, 27, 28, 31–33, 42, 45, 47, 48, 53, 56, 57, 60, 61, 69, 70, 73, 97, 103, 114).

Additional concerns relate to data accessibility and population coverage. In some cases, registry data are not easily accessible for research or regulatory purposes. Moreover, incomplete reporting or limited representation of specific patient groups may reduce the overall representativeness of the data and affect regulatory authorities’ acceptance of registry-based evidence (27, 53, 73, 103).

From a methodological perspective, most registries rely on observational data, which are inherently more susceptible to bias and confounding than randomized studies. To mitigate these limitations and strengthen the reliability of the findings, sophisticated statistical approaches, such as propensity score techniques or Bayesian analytical methods, are often required (7, 27, 32–34, 42, 47, 48, 61, 63, 69, 70, 76, 97, 114).

Finally, uncertainty still exists regarding how registry data should be used within regulatory frameworks. Although certain regulatory systems recognize and accept such evidence under defined conditions, their acceptance varies across jurisdictions and typically depends on strict methodological requirements and high data-quality standards (28, 31, 32, 45–48, 53, 56, 57, 60–63, 69, 70, 76, 97, 103, 114).

#### 3.4.4 Variability and gaps in literature

The literature review reveals several recurring sources of heterogeneity and key gaps that limit the regulatory usefulness of registries for MDs and IVDs.

Many registries lack well-defined comparator groups. This limitation reduces their value for comparative effectiveness analyses and diminishes their potential contribution to regulatory evaluation processes (32, 46–48, 53, 60, 62, 63, 76, 103, 114).

Finally, concerns regarding sustainability and governance structures remain prominent. Differences in funding mechanisms, governance arrangements, and the degree of stakeholder involvement can affect the long-term maintenance, expansion, and overall reliability of registry initiatives (27, 34, 42, 57, 69, 97).

### 3.5 Post-market surveillance

PMS is generally described as an ongoing, lifecycle-based process that is integrated into manufacturers’ quality management systems and regulatory oversight frameworks. It includes both reactive (passive) and proactive (active) activities, with a growing emphasis on shifting from mainly complaint-based surveillance toward more structured, proactive evidence generation.

#### 3.5.1 Overview of methods

Reactive or passive PMS activities collect information after events occur and rely primarily on spontaneous or user-initiated reporting. These include customer complaints and incident reports, reports of serious incidents and adverse events within vigilance systems (including field safety corrective actions and field safety notices), spontaneous reporting systems operated by authorities or manufacturers, as well as service and maintenance reports, device fault reports, trend reporting, literature reviews, and independent post-market clinical studies (7, 24, 27, 31, 33, 37, 47, 48, 56, 59, 60, 103, 106, 113). Although these systems provide broad coverage and are relatively low-cost, they have important limitations. Passive PMS is often affected by underreporting, incomplete or delayed reports, and heterogeneous data, which limits the ability to accurately estimate event rates or confirm suspected safety signals (31, 33, 37, 47, 48, 59, 60, 100, 103, 106, 113).

Proactive or active PMS involves the planned, systematic collection of data from real-world devices used to detect safety or performance issues earlier and reduce remaining uncertainties after market entry. Unlike reactive approaches, it typically relies on structured, study-like methods (28, 46, 59, 61, 62, 91, 93, 103, 106). Key proactive activities include PMCF for medical devices and post-market performance follow-up (PMPF) for IVDs, which involve the systematic collection of clinical or performance data under routine conditions. These activities may include new clinical investigations, extensions of pre-market studies, or studies based on RWD. Proactive PMS may also involve post-market clinical investigations or confirmatory trials, including RCTs or observational study designs, particularly when devices are introduced with limited pre-market evidence (7, 24, 30, 31, 33, 46–48, 56, 57, 60–62, 100, 103, 106). Additional approaches include structured user and patient feedback, manufacturer-sponsored device registries, and surveillance using large healthcare data sources, such as electronic health records, claims databases, and registry networks. Together, these systems support the prospective and structured identification and evaluation of device-related risks (24, 27, 28, 30, 33, 46, 48, 56, 59, 91, 93, 100, 103, 106).

Several analytical methods are used to analyse PMS data. Signal detection in spontaneous reporting systems often uses disproportionality analyses and other statistical approaches, including Bayesian, machine-learning, and sequential monitoring methods (27, 56, 59). PMS also relies on observational study designs, such as cohort or case-control studies, with statistical techniques to control for confounding and bias (27, 31, 59, 61). In addition, near-real-time monitoring methods and simulation frameworks may be used, particularly for newly marketed high-risk devices (27, 28, 93).

**Figure 5:**
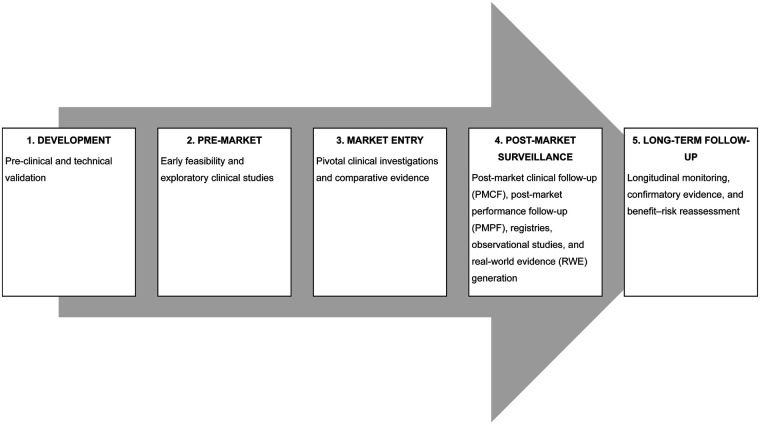
Evidence generation across the medical device life cycle for high-risk and innovative devices and IVDs.

 Figure 5 positions post-market surveillance within the broader life-cycle framework, emphasizing its role in generating continuous evidence and ongoing reassessment of device safety, performance, and the benefit–risk balance.

#### 3.5.2 Regulatory considerations

PMS is consistently described as a key regulatory requirement under the EU MDR and EU IVDR and is positioned within a broader framework of continuous, lifecycle-based evidence generation (1, 2). It is defined as a core component of the manufacturer's quality management system and is mandated under Article 83 of the EU MDR, while the IVDR similarly incorporates PMS through PMPF requirements. PMS activities continue after CE marking (fr. *Conformité Européenne*, CE), particularly for devices granted accelerated or conditional approvals based on preliminary evidence. In this context, PMS plays an essential role in collecting RWD on safety, performance, and clinical benefits that may not be fully established during pre-market evaluation (7, 24, 30, 31, 33, 46–48, 56, 57, 60–62, 91, 100, 103, 113). The literature also emphasizes that access to accelerated or early-access pathways often requires clear commitments to post-market evidence generation, such as confirmatory clinical studies, registries, or RWE programs. Failure to fulfil these obligations may lead to regulatory consequences, including non-compliance findings, delays in surveillance audits, or even the device's market withdrawal (24, 31, 46, 62).

Regulatory tools and infrastructures are increasingly used to support more structured post-market surveillance. In the EU, systems such as UDI and EUDAMED aim to improve device traceability, enable linkage across PMS datasets, and facilitate monitoring of manufacturers’ post-market evidence commitments (31, 37, 60, 106, 113). In the U.S., initiatives such as NEST and active-surveillance networks use RWD to support both pre-market and post-market regulatory decisions and to pilot registry-based surveillance approaches (27, 28, 61, 93, 106). For AI-enabled devices, emerging regulatory concepts, including real-world performance monitoring and Algorithm Change Protocols, reflect a shift toward continuous PMS integrated with the software lifecycle, where significant modifications may trigger regulatory re-evaluation (91).

Risk-based and modular approaches to PMS planning are widely recommended in the literature. The intensity of PMS and PMCF/PMPF activities should scale with factors such as device risk class, novelty, and clinical context, with more rigorous post-market evidence expected for high-risk, innovative, or paediatric devices (7, 24, 30, 31, 33, 46–48, 56, 57, 59, 91, 100, 103, 106, 113). Some sources also propose modular PMS structures, where a core PMS plan defines overarching processes and methodologies, while device-specific modules outline tailored surveillance activities. Early dialogue with regulators and notified bodies is also encouraged to ensure that planned PMS and PMCF/PMPF activities meet conformity assessment expectations (24, 30, 56).

PMS serves several key regulatory functions throughout the device lifecycle. It helps verify and refine benefit–risk profiles by confirming clinical claims and identifying previously unrecognized risks or long-term outcomes. PMS also supports ongoing updates of clinical and performance evaluations, risk management documentation, and mandatory regulatory reports. In addition, PMS findings may trigger regulatory actions such as design or labelling changes, recalls, or market withdrawal, while the generated RWE can inform health technology assessments, reimbursement decisions, and clinical guidelines (7, 24, 27, 28, 30, 31, 33, 37, 46–48, 56, 57, 59–62, 91, 93, 100, 103, 106, 113).

#### 3.5.3 Limitations and challenges

The literature highlights significant limitations, including underreporting, incomplete or non-standardized information, lack of denominator data, and delayed signal detection. As a result, these sources are generally considered to provide low-quality evidence and are primarily used for trend detection and hypothesis generation rather than for confirming device safety and performance (7, 24, 27, 28, 31, 33, 37, 47, 48, 56, 59, 60, 100, 103, 106, 113).

Furthermore, the literature also highlights significant challenges, including heterogeneous governance and data quality across jurisdictions, incomplete national or international coverage, limited interoperability between registries, and the substantial resources required to establish and maintain them. In many areas, particularly for certain device types and paediatric devices, registries remain limited or only partially developed (24, 27, 28, 30, 31, 33, 37, 46, 48, 56, 57, 60, 61, 103, 106, 113).

Active surveillance systems based on RWD and RWE offer important advantages for PMS. They enable large-scale and near-real-time monitoring of devices in routine clinical practice, capturing diverse patient populations, operator learning curves, and iterative device modifications. Such systems can also support advanced analytical approaches, including machine learning and natural language processing, to identify complex safety or performance patterns (7, 27, 28, 30, 31, 33, 37, 59–61, 93, 103, 106). However, the literature highlights persistent challenges, including data quality and completeness issues, fragmented datasets across institutions, and limited interoperability. Additional barriers include privacy and data-protection constraints, the need for specialized analytical expertise, and, in the case of AI-enabled or connected devices, difficulties in distinguishing device malfunctions from data-quality issues or cybersecurity issues (7, 24, 27, 28, 30, 31, 33, 37, 48, 56, 59–61, 91, 93, 103, 106).

Structured post-market follow-up activities, including PMCF and PMPF studies as well as post-market trials, are widely recognized as essential for addressing evidence gaps and verifying the ongoing safety and performance of medical devices (7, 24, 30, 31, 33, 46–48, 56, 57, 59, 61, 62, 100, 103, 106). However, the literature highlights several recurring challenges. Post-approval studies are often difficult to recruit for and complete, with relatively low completion rates for high-risk devices. Methodological limitations are also common, including non-randomized or unblinded designs and reliance on surrogate endpoints. Additional difficulties arise in small or specialized populations, such as paediatric or rare-disease contexts, where statistical power and long-term follow-up are limited. Resource constraints, particularly for small and medium-sized enterprises, and variable expectations from notified bodies further complicate the design and implementation of PMCF and PMPF activities. Despite these challenges, such studies remain critical for confirming clinical claims, refining benefit–risk assessments (BRA), and addressing residual uncertainties after market entry (24, 28, 30, 31, 57, 59, 61, 62, 100, 103, 106).

Several systemic and governance challenges limit the effectiveness of PMS. These include fragmented PMS infrastructures, particularly in Europe, limited enforcement of post-market commitments, and insufficient incentives for rigorous post-market studies. Additional barriers include clinician underreporting, institutional resistance to new data-sharing technologies, and resource asymmetries that favour larger manufacturers, especially for AI-enabled or connected devices (7, 24, 27, 28, 30, 31, 33, 37, 46, 56, 57, 60–62, 91, 100, 103, 113).

#### 3.5.4 Variability and gaps in literature

The literature varies in the depth and specificity with which PMS methodologies are described. Detailed methodological frameworks, such as registry-based architectures, RWE study designs, and signal-detection approaches, are discussed alongside more general descriptions that mainly highlight weaknesses in current practice. In emerging areas such as AI-enabled devices or blockchain-based PMS, discussion remains largely conceptual with limited practical evidence (7, 24, 28, 30, 31, 37, 46, 56, 59, 60, 62, 91, 93, 100, 104, 106, 113). Recurring gaps include limited and inconsistent post-market comparative evidence, a lack of harmonized guidance on registry governance and data quality, insufficient standardization of datasets and device identifiers, restricted transparency and data sharing, and inadequate infrastructure and funding for registries and RWD systems (7, 24, 28, 30, 31, 33, 37, 46, 56, 57, 60–62, 93, 100, 103, 106, 113).

### 3.6 Life-cycle assessment

**“**Life-cycle” refers to the continuous generation of clinical and performance evidence for high-risk medical devices and IVDs, from early development to post-market use. It emphasizes the generation of ongoing evidence through frameworks that connect pre-clinical research, clinical trials, and post-market RWE throughout the device's lifespan.

#### 3.6.1 Overview of methods

Life-cycle assessment (LCA) refers to the continuous, stage-linked evaluation of safety, performance, and benefits and risks across a device's entire lifespan, rather than a one-time pre-market assessment. Clinical evaluation is described as a TPLC process, beginning in design and development and continuing through approval, post-market surveillance, and long-term follow-up, with evidence generated and reassessed throughout (5, 30, 36, 46, 47, 49, 58, 64, 107, 108).

Life-cycle assessment is applied across several overlapping dimensions, including clinical development stages, regulatory processes, and evidence planning. It spans from pre-clinical testing and early feasibility studies through exploratory and pivotal clinical trials to post-market registries and long-term follow-up. It also covers regulatory stages, such as early evidence strategy selection, market approval based on clinical evaluation reports, and ongoing PMS and clinical follow-up. In addition, adaptive assessment models and staged frameworks (e.g., IDEAL/IDEAL-D, standing for Idea, Development, Exploration, Assessment, and Long-term study/Device) link early studies, regulatory decisions, and continuous evidence updates throughout the device's lifespan (5, 30, 36, 46, 47, 49, 58, 64, 107, 108).

The literature describes several structured models that operationalize life-cycle thinking. These include IDEAL/IDEAL-D frameworks, which define staged development from preclinical and early human studies to comparative trials and long-term follow-up (46, 107, 108); four-stage evidence schemes, outlining sequential phases from pre-clinical and pre-market research to post-market use and eventual replacement (5, 47); and modular clinical investigation plans (CIPs) that integrate pre-market studies with PMS and follow-up within a flexible protocol structure (30). For digital technologies, lifecycle-integrated approaches include multidimensional assessment frameworks for mobile medical apps, Content–Context–Process models with “living” HTA for decision-support software, and programs such as the FDA Software Precertification Program that rely on continuous monitoring across the software lifecycle (58, 64). Overall, these models frame the lifecycle as a planned, iterative evidence strategy spanning the pre-market, approval, and post-market stages.

A life-cycle framework for evidence generation, highlighting that different evidence-generation activities contribute at distinct stages throughout the device lifecycle, is presented in Figure 5.

#### 3.6.2 Regulatory considerations

The EU MDR (1) introduces a lifecycle approach to device regulation (Articles 61, 83–86), shifting from one-time certification to continuous generation of clinical evidence. It requires ongoing PMS, PMCF, and PSURs (Articles 83–86, Annex XIV Part B, Articles 85–86) to ensure that safety, performance, and benefit–risk are monitored throughout a device's lifetime. EU MDR also structures clinical evaluation from pre-market development to post-market follow-up (5, 30, 49, 64, 107).

Several models are presented as tools that align with MDR's life-cycle approach. Modular CIPs integrate pre-market studies with post-market PMCF and PMS activities within a single evidence plan. Similarly, IDEAL-D frameworks are mapped to EU MDR processes, linking early development stages to CE marking decisions and later stages to post-market follow-up and ongoing safety monitoring (30, 107).

High-risk or innovative IVDs are mentioned within the same life-cycle evaluation framework as MDs. Benefit assessment and clinical evaluation are described as continuous processes that extend from design and development through PMS. However, the texts do not provide a detailed discussion of EU IVDR provisions or specific regulatory requirements (36, 49).

Life-cycle evidence approaches are also reflected in other regulatory systems. China's NMPA links device risk and novelty to pre-market evidence strategies that connect to later lifecycle stages (49). In the U. S., FDA mechanisms such as conditional approvals, post-approval studies, and the Software Precertification Program support lifecycle oversight, particularly for digital technologies (49, 58, 64). Similar challenges with rapidly evolving software are noted in Australia's TGA framework (58). Overall, lifecycle-based evidence generation is used internationally, although the MDR is often described as particularly explicit in its post-market requirements.

#### 3.6.3 Limitations and challenges

Life-cycle approaches face several practical challenges. Their implementation requires complex coordination and careful planning, including correct identification of the innovation stage and collaboration among regulators, HTA bodies, and manufacturers. Many models depend on robust registries and long-term data, but gaps in registry coverage and data completeness can limit comprehensive safety and effectiveness assessments (46, 108). In addition, regulatory guidance remains incomplete in some areas, leaving uncertainty about how lifecycle clinical development should be planned in practice, particularly for rapidly evolving technologies such as software (46, 58, 64). Lifecycle strategies can also be difficult to implement across different regulatory jurisdictions, where requirements for clinical evaluation, post-approval studies, and surveillance differ (49). Finally, inconsistent terminology and classification of innovation can complicate stage assignment and hinder the consistent application of lifecycle evidence frameworks (108).

#### 3.6.4 Variability and gaps across literature

The literature uses various terms for similar concepts, including “life-cycle oriented,” “lifecycle-long,” “total product lifecycle,” “adaptive benefit assessment,” and “ongoing clinical evaluation.” Terminology is not always consistent, and some authors note that vague labels such as “new” or “innovative” can obscure the actual level of evidence and uncertainty, complicating lifecycle-based evidence planning and governance (31, 58, 108).

The level of practical detail in lifecycle approaches varies across the literature, ranging from structured frameworks with defined stages, study types, and tools such as modular clinical investigation plans or IDEAL-D mappings to EU MDR processes to more general descriptions of continuous evidence generation without detailed guidance on study design, data standards, or governance. The MDR establishes strong lifecycle requirements but leaves many practical aspects, such as clinical development planning, to supporting frameworks or manufacturer-developed strategies (30, 46, 47, 64, 107).

Several areas of lifecycle approaches remain underdeveloped. Although the EU MDR requires continuous clinical evaluation and PMCF, it does not provide a clearly defined stepwise methodology for evidence generation, creating a need for supporting frameworks. Lifecycle models also rely heavily on registries for long-term evidence, yet limitations in registry coverage and data completeness indicate gaps in practical infrastructure. In addition, regulatory oversight of rapidly evolving technologies remains challenging, with lifecycle-based approaches, such as living HTA or precertification models, still emerging rather than fully established (46, 58, 64, 107).

### 3.7 Other topics

#### 3.7.1 Overview of methods

The reviewed literature highlights a range of methodological approaches that go beyond conventional classifications of study designs or evidence types. These approaches are especially important for assessing high-risk and innovative MDs and IVDs. By introducing new frameworks and analytical tools, they help address evolving regulatory requirements and emerging technical challenges.

The literature also describes BRA as a regulatory requirement for MDs and IVDs under frameworks such as the EU MDR, the US Federal Food, Drug, and Cosmetic Act (FD&C Act), and international standards including ISO 14971 and ISO/TR 24971. BRA approaches include qualitative frameworks (e.g., FDA and ISO models), quantitative methods such as Multicriteria Decision Analysis (MCDA), modelling approaches like Health Outcomes Modelling (HOM), metrics including Net Benefit Score (NBS) and Benefit–Risk Ratio (BRR), Quantitative Benefit–Risk Determination (QBRD), and hybrid methods combining qualitative and numerical elements (e.g., Bayesian networks). These approaches support the systematic identification and comparison of benefits and risks, although no single method is prescribed across jurisdictions (110).

Synthetic data generation and validation use advanced machine learning methods, such as generative adversarial networks (GANs) and variational autoencoders (VAEs), as well as statistical techniques, to produce artificial datasets that mimic the statistical characteristics of real patient data. These approaches emphasize privacy protection and utility assessment, often applying metrics such as the multivariate Hellinger distance to evaluate data fidelity and support regulatory confidence (22).

Mathematical and computational modelling includes in silico models and simulations used to predict device safety and performance, particularly when clinical trials are impractical or ethically challenging. Examples include simulation studies, computational analyses of MRI-induced heating in pacemakers, and emerging stochastic engineering models that simulate outcomes in virtual patients (32, 41).

Clinical simulation and consensus frameworks use structured simulation studies to assess the usability, safety, and performance of software as a medical device (SaMD), often relying on synthetic patient cases and simulated clinical settings. Frameworks such as SIROS, developed through expert consensus methods (e.g., eDelphi), establish methodological standards for generating regulatory-grade evidence (38).

Clinical usability studies integrate human factors engineering with clinical research to evaluate usability and clinical performance over extended periods. They follow structured protocols for training, data collection, and participant selection, and are conducted in both laboratory and real-world settings (40).

AI-specific validation approaches and performance metrics are used to evaluate AI-based diagnostic devices. They emphasize external validation with independent datasets and rely on technical performance measures such as Dice coefficient, ROC/AUROC, and calibration accuracy, which support generalisability and regulatory relevance (72).

AI methods are also applied in ISCTs, where they generate synthetic patient cohorts, analyse simulation outputs, and support digital twin–based evaluations. These approaches complement mechanistic modelling within structured VVUQ frameworks (e.g., ASME V&V 40) and include machine learning models such as neural networks and support vector machines (111).

The literature also proposes a structured, risk-based approach for the clinical evaluation of AI-enabled medical device software, implemented through the CORE-MD AI Risk Score. This system combines three evidence domains: valid clinical association, valid technical performance, and clinical performance, to determine the level and timing of evidence required throughout the AI life cycle (115).

#### 3.7.2 Regulatory considerations

Synthetic data remains an emerging area and currently lacks explicit regulatory provisions within European frameworks such as ENCePP, MDR, and IVDR. However, exploratory initiatives under Horizon Europe and DARWIN EU are working to define potential pathways for its regulatory acceptance (22).

Modelling and simulation are increasingly recognized by regulators such as the FDA and EMA when models are properly validated and demonstrate sufficient credibility. In some high-risk device contexts, they may be accepted as primary evidence, typically alongside PMS requirements (32, 38, 41).

Simulation frameworks, including SIROS, have also been used to support regulatory submissions, and the FDA has accepted simulation-based evidence for SaMD in certain cases (38, 41).

Usability studies must determine whether clinical trial regulations apply, and engagement with regulatory authorities is often recommended during study design (40).

For AI validation, regulatory approval requires demonstrating technical performance using independent datasets, and, in some cases, reimbursement decisions may also require evidence of clinical utility (72).

With respect to BRA, regulatory authorities expect a transparent, well-documented rationale explaining why the benefits outweigh the residual risks. FDA guidance provides examples of qualitative judgment where uncertainties remain, while ISO TR 24971 outlines structured steps for determining the acceptability of residual risks and for conducting an overall BRA. Despite the regulatory requirement to perform a BRA, neither EU nor US legislation formally endorses a specific quantitative model, and acceptance of particular methodologies is not guaranteed. Consequently, manufacturers retain methodological flexibility but also bear responsibility for demonstrating that their chosen approach is scientifically justified, consistent, and clearly explained (110). For ISCT applications, regulators require documented validation, uncertainty quantification, and risk-based credibility assessment of AI models. Acceptance depends on demonstrated robustness and compliance with relevant regulatory frameworks (111). The CORE-MD AI Risk Score is positioned as a potential tool for future EU regulatory guidance and is reportedly under consideration within MDCG working groups for integration into formal processes. It aligns AI-specific methodological tasks, such as data processing, model calibration, drift monitoring, and version control, with MDR and IVDR evidence requirements across the product life cycle (115).

#### 3.7.3 Limitations and challenges

Qualitative methods may lack transparency in trade-off reasoning, while quantitative methods can be complex, data-intensive, and dependent on assumptions that may not hold for devices with multiple benefits and heterogeneous risk profiles. Emerging risks, such as cybersecurity vulnerabilities, AI-related uncertainties, or human-factors-related harms, are often difficult to quantify due to limited data. Inconsistent methodological application and incomplete evidence further constrain the practical robustness of BRA (110).

On the other hand, synthetic data is constrained by the lack of well-established regulatory standards and methodological guidance in Europe, as well as ongoing concerns about validation and privacy (22). Modelling and simulation require rigorous validation against experimental or clinical data, and their regulatory acceptance depends on the credibility and transparency of the models used (32, 41). Simulation frameworks depend on high fidelity and representativeness but may be prone to bias and can be difficult to apply in complex clinical contexts (38, 41). Usability studies may be affected by participant interaction and training effects, and balancing safety with study validity can be challenging (40). For AI validation, generalisability may be limited by heterogeneous datasets, and internal validation alone is insufficient to demonstrate reliability (38, 72).

In ISCT contexts, AI enables scalable synthetic cohorts and predictive simulations but faces limitations, including regulatory uncertainty, limited real-world validation, algorithmic bias, and transparency challenges. Structured VVUQ is essential for regulatory acceptance (111).

The CORE-MD scoring approach offers transparency and proportionality by structuring AI evidence requirements across three domains, but may oversimplify complex and dynamic AI risks. Limitations include unresolved methods for assessing explainability safety, uncertainty around the reliability of human oversight as a mitigation measure, and challenges posed by drift, off-label use, and adaptive algorithms (115).

#### 3.7.4 Variability and gaps in literature

The literature identifies several areas of variability and limited development in the application of novel methods for generating evidence. A major gap is the lack of harmonized regulatory and methodological standards for synthetic data, although initiatives are underway to address this (22, 38). While modelling and simulation are gaining broader acceptance, their use requires strong validation and transparency. The literature notes that practical guidance on uncertainty quantification and establishing model credibility remains limited (22, 32, 41). Simulation studies also face challenges in achieving sufficient fidelity and representativeness, particularly when replicating complex clinical scenarios or generating synthetic patient data (22, 38, 41). In AI-related research, concerns about algorithm generalisability persist, highlighting the need for diverse training datasets and robust external validation strategies (72). Finally, although usability studies are increasingly recognized as important when user interaction affects clinical outcomes, best practices for integrating human factors with clinical research methods are still evolving (40).

For BRA specifically, the literature identifies substantial variability in methodological application, reflecting the absence of a standardized “one-size-fits-all” approach. Differences in endpoint selection, weighting schemes, use of patient preference data, and treatment of uncertainty lead to inconsistent regulatory submissions. Quantitative methods may not adequately capture complex benefit–risk profiles of innovative or digital devices, while qualitative approaches may lack sufficient clarity in documenting trade-offs. Overall, the literature concludes that integrating qualitative and quantitative elements may provide the most practical path forward, but clearer methodological guidance is needed to ensure consistency, transparency, and regulatory confidence (110). For AI in ISCTs, gaps remain in harmonized validation standards, transparency requirements, and cross-regulatory alignment, limiting consistent regulatory uptake (111). Regarding AI-specific risk-scoring approaches, further validation and empirical testing are needed to confirm the reliability and inter-rater consistency of numeric thresholds across device types. Additionally, harmonized guidance on integrating explainability assessment and oversight of adaptive algorithms into structured scoring systems remains underdeveloped (115).

## 4 Discussion

High-risk and innovative MDs and IVDs in the EU are developed and assessed within a regulatory framework defined by the MDR and IVDR (1, 2). The findings of this scoping review indicate that evidence generation for these technologies cannot rely on a single methodological approach but instead requires a flexible, lifecycle-based framework that integrates multiple study designs. While randomized controlled trials remain an important source of high-quality evidence, observational studies, real-world evidence, registries, and structured post-market surveillance are essential for capturing long-term safety and effectiveness. However, current practices remain characterized by variability, methodological limitations, and regulatory inconsistencies. Addressing these challenges through strengthened methodological standards, improved data quality, and greater regulatory harmonization will be critical to ensuring that lifecycle evidence generation can effectively support the safe and timely introduction of emerging medical technologies. Although this review was primarily framed within the context of the EU MDR and IVDR regulatory frameworks, relevant international regulatory approaches, including those from the FDA and NMPA, were also considered to provide comparative methodological and contextual perspectives. Consequently, conclusions related to conformity assessment procedures, notified body practices, and regulatory implementation should be interpreted primarily within the EU context, whereas observations regarding lifecycle evidence generation, real-world evidence, registries, and methodological challenges reflect broader international trends across medical device regulatory systems.

For high-risk implantable devices, evidence from the product's total life cycle is advocated, with clearly defined phases from preclinical testing, through early clinical studies and randomized trials before marketing, to registries and RWD in the phase of widespread use and when the technology becomes obsolete (5, 6). Such an approach emphasizes that the generation of clinical evidence must be a continuous and strategically planned process, with early involvement of regulators, HTA agencies, clinicians, patients, and industry (5, 6).

Despite stricter regulations, manufacturers and notified bodies report significant administrative and operational burdens, including duplicate documentation and non-harmonized requirements, and there is a call for simplification and stronger application of a risk-based approach without compromising safety (118). In addition, manufacturers must pay greater attention to generating high-quality, direct, and statistically robust evidence regarding the safety and performance of their devices (60, 65). They face serious challenges in evaluations carried out by notified bodies, as half of MD manufacturers and a third of IVD device manufacturers report that they have at least once experienced a significant challenge to a clinical or performance evaluation, often resulting in delays or adverse certification outcomes, the leading cause of which is the lack of a clear definition of what constitutes a sufficient level of clinical evidence, which is reported for both the IVD sector and the MD sector (119). An additional level of unpredictability is created by the highly inconsistent acceptance of RWE among NBs, with some not accepting RWE as a source of evidence at all. However, the relevant literature highlights the key role of RWE in monitoring the performance and safety of high-risk devices throughout their entire life cycle (6, 9, 119). Also, analyses of market entry of high-risk devices in Europe show that many innovative products receive CE marking based on limited and methodologically weak clinical evidence, often without their own RCTs (60). A similar pattern has been observed in hospital HTA, where most innovative devices considered for introduction into clinical practice are based on low-level evidence studies, with small numbers of patients and insufficiently long follow-up, particularly for implantable products (120).

At the level of evidence generation strategy, an integrated approach in which traditional randomized trials are planned alongside real-world studies, registries, and economic analyses, rather than in a sequential, fragmented manner, is increasingly essential (6, 121). Digital technologies are further transforming evidence generation methodologies: EHRs, mobile apps, wearables, and artificial intelligence enable more efficient study planning, recruitment, remote monitoring, and continuous data collection (122, 123). Clinical and performance evidence planning for high-risk devices is increasingly based on structured, question-based approaches taken from drug development. Starting from a set of key questions about the mechanism of action, safety, target population, and expected outcomes, a Target Product Profile (TPP) is developed to define minimum requirements and measurable objectives and to guide study design and go/no-go decisions (81). Such a framework facilitates the early identification of uncertainties, the design of adjustments before costly testing, and improved communication among manufacturers, regulators, and financiers (6, 81).

Within this context, the EU IVDR reinforces a risk-based approach to evidence generation, requiring higher-risk devices (Classes C and D) to demonstrate both analytical and clinical performance and to undergo more rigorous conformity assessment procedures (2, 7). The level and robustness of evidence required increase with device risk, with particular emphasis on clinical performance supported by appropriate clinical studies, scientific literature, or real-world data. For the highest-risk devices, more robust, methodologically rigorous evidence, often derived from prospective or well-designed retrospective studies, is expected, alongside enhanced post-market obligations, such as periodic safety reporting (2, 7). These requirements reflect a shift towards more stringent, continuous evaluation of IVD performance throughout the device lifecycle.

All of the above indicate that Europe is undergoing a transitional period in which the regulatory system is modernizing and aligning with the highest international standards. At the same time, it is accompanied by significant challenges, including administrative complexity, inconsistencies among notified bodies, a lack of transparency, and uncertainty regarding the interpretation of clinical evidence requirements (60, 65, 118).

As this was a scoping review, no formal assessment of methodological quality or risk of bias was performed. Consequently, conclusions regarding the strength of the evidence should be interpreted with caution. In addition, a substantial proportion of the included literature consisted of non-empirical and guidance-oriented sources, reflecting the evolving nature of the field. The included literature is highly heterogeneous in study design, data sources, and regulatory contexts, limiting comparability and precluding quantitative synthesis. Differences in regulatory frameworks may limit the generalisability of the findings to the European context, while publication and selection biases may have influenced the included evidence. In addition, many identified evidence sources, particularly RWD, are subject to limitations related to data quality and standardization. Finally, the rapidly evolving nature of medical technologies and regulatory frameworks means that some findings may become outdated.

## 5 Conclusions

Evidence generation for high-risk and innovative MDs and IVDs requires a flexible, lifecycle-based approach that combines multiple study designs. While RCTs remain important, observational studies, RWE, and PMS are essential for capturing long-term safety and effectiveness. Strengthening methodological standards, improving the quality of RWD, and enhancing regulatory harmonization will be important for supporting robust evaluation of innovative medical technologies.

## Data Availability

The original contributions presented in the study are included in the article/Supplementary Material, further inquiries can be directed to the corresponding author.
