# Supplementary material for: Methodologies for generating and evaluating clinical and performance evidence for high-risk and innovative medical devices and in vitro diagnostics: a scoping review
Source: Front Med Technol. 2026 Jun 24;8:1857401. doi: 10.3389/fmedt.2026.1857401 (PMC13341693; doi:10.3389/fmedt.2026.1857401)
Supplement: Supplementary file 1 [file Datasheet1.pdf]

Methodologies for Generating and Evaluating Clinical and Performance Evidence for High-Risk and Innovative Medical Devices and In Vitro Diagnostics: A Scoping Review

Search strategies

I. Web of Science (WOS)*:

1 title search

```
ti=(
 regulatory or
   proposal or
CE-mark* or
   regulations or
   market or
   evidence or
clinical investigat* or
clinical trial* or
   ((Quality or transparency or gap* or strength or real-world or life-cycle or generating or long-term)
near/2 (evidence or data)) or
   registr* or
   Surveillance or
   premarket or
   pre-market or
   postmarket or
PMS
   post-market or
"post market" or
   FDA or
   "US Food and Drug Administration" or
   approval* or
   Approved or
   classification or
certific* or
   recommendations or
   guidelines or
   "European Union Medical Devices" or
   "EU  Medical Device Regulation" or
MDR or
IVDR or
"EU In Vitro Diagnostic Medical Device Regulation" or
   "ISO standards" or
   "life-cycle" or
   "health technology assessment" or
HTA or
   framework or
   landscape or
   statements or
   Opportunities or
   Threats or
```

method* or
Methodologies or
testing or
report* or
benefit* or
cost* or
economic or
"benefit-risk" or
studies or
safety or
quality or
measures or
efficacy or
legislat* or
standards or
approval or
Effectiveness or
safety or
authorisation or
panel or
meta-analysis or
review or
"combined studies" or
"bridging studies"
cross-sectional or
retrospective or
comment
)

2 Field search abstract (high-risk)

(ab=(
(("medical device*" or "medical product*" or "healthcare device*" or "health care device*" or "therapeutic device*" or "diagnostic device*" or "medical equipment*" or "medical technolog*" or "medical apparatus*" or "clinical device*" or "biomedical device*")
near/3
(high-risk or "high risk" or "highest risk" or "class III" or "class 3" or "class D" or "risk class III" or "critical" or "life-support*" or "life support*" or "life sustain*" or "life-sustain*" or "safety critical" or "safety-critical" or "high hazard" or "high-hazard" or "high concern" or "high-concern" or "significant risk" or implantable or invasive or "life threatening" or "life-threatening" or "critical care" or "major surgical" or "high complexity" or "high-complexity" or "substantial risk" or "serious risk" or "serious adverse" or "severe risk" or "maximum risk" or "elevated risk" or "priority device*"))
or
("implantable device*" or "class IIb" or "active implant*" or "implantable medical*" or "class III device*" or "class 3 device*" or "class D device*" or "critical medical device*" or "critical care device*" or "life support device*" or "life-sustaining device*" or "high risk implant*" or "high-risk medical*" or "highest risk device*")
or
(("in vitro" or "in-vitro" or IVD) near/1 (device* or diagnostic* or instrument* or equipment* or kit* or reagent* or calibrator* or instrument* or apparat* or software or system))
or

(("laboratory diagnostic*" or "clinical diagnostic*") near/2 (device* or diagnostic* or instrument* or equipment*) near/3 (high-risk or "high risk" or "class D" or "highest risk" or critical or "serious public health threat" or "significant public health threat"))
or
(cardiovascular or radiation or neurological or neurosurgical or orthopedic or cardiac or brain or spinal or neural or "drug delivery" or "life support") near/3 (implant* or device* or equipment*) near/3 (high-risk or "high risk" or "class III" or critical)
))

#3 Field search abstract (innovative)

(ab=(
(("innovative" or "novel" or "emerging" or "advanced" or "next generation")
near/3
("medical device*" or "healthcare device*" or "health care device*" or "therapeutic device*" or "diagnostic device*" or "medical equipment*" or "medical technolog*" or "clinical device*" or "biomedical device*" or "medical product*" or "medical instrument*" or "health monitoring device*" or "patient monitoring device*"))
or
("digital health device*" or "digital health tool*" "digital healthcare device*" or "digital healthcare tool*" or "connected medical device*" or "wireless medical device*" or "wearable medical device*" or "point of care device*" or "remote patient monitoring device*" or "medical sensor device*" or "medical robot*" or "surgical robot*" or "medical AI device*" or "smart medical device*" or "portable medical diagnostic*" or "AI-enabled medical device*" or "telehealth device*" or "telemedicine device*" or telerehabilitation or SaMD)
or
("medical organ-on-chip" or "medical lab-on-chip" or "medical 3D print*" or "medical bioprint*" or "medical additive manufactur*" or "medical nanodevice*" or "medical nanoscale device*" or "regenerative medicine device*" or "tissue engineering device*")
or
(("minimally invasive" or "robotic assisted") near/3 ("medical device*" or "surgical device*" or "diagnostic device*" or "therapeutic device*"))
Or
(medical near/2 software) or (AI near/2 software near/2 medical)
))

#1 AND (#2 OR #3)
*NOT Derwent Innovations Index

II. PubMed:

((((("Software"[MeSH Terms] OR "Mobile Applications"[MeSH Terms] OR "telerehabilitation"[MeSH Terms] OR "Medical Informatics"[MeSH Terms] OR "Medical Informatics Applications"[MeSH Terms] OR "decision support systems, clinical"[MeSH Terms] OR "Information Technology"[MeSH Terms] OR "Telemedicine"[MeSH Terms] OR "Remote Sensing Technology"[MeSH Terms] OR "Artificial Intelligence"[MeSH Terms] OR "Machine Learning"[MeSH Terms] OR "Deep Learning"[MeSH Terms] OR "Natural Language Processing"[MeSH Terms] OR "image processing, computer assisted"[MeSH Terms] OR "Electronic Health Records"[MeSH Terms] OR "Mobile Applications"[MeSH Terms] OR "Wearable Electronic Devices"[MeSH Terms] OR "Internet of Things"[MeSH Terms] OR "Virtual Reality"[MeSH Terms] OR "Augmented Reality"[MeSH Terms] OR "Digital Technology"[All Fields] OR "mHealth"[All Fields] OR "Digital Health"[All Fields] OR "Digital Therapeutics"[All Fields] OR "Software as a Medical Device"[All Fields] OR "SaMD"[All Fields]) AND ("Technology"[MeSH Terms] OR

"innovative"[Title/Abstract] OR "innovation"[Title/Abstract] OR "novel"[Title/Abstract] OR "emerging technology"[Title/Abstract] OR "new technology"[Title/Abstract] OR "breakthrough"[Title/Abstract] OR "cutting edge"[Title/Abstract] OR "next generation"[Title/Abstract] OR "advanced technology"[Title/Abstract])) OR ("Heart Valve Prosthesis"[MeSH Terms] OR "defibrillators, implantable"[MeSH Terms] OR "Cardiac Resynchronization Therapy Devices"[MeSH Terms] OR "pacemaker, artificial"[MeSH Terms] OR "Heart-Assist Devices"[MeSH Terms] OR "Deep Brain Stimulation"[MeSH Terms] OR "Vagus Nerve Stimulation"[MeSH Terms] OR "Neural Prostheses"[MeSH Terms] OR "Spinal Cord Stimulation"[MeSH Terms] OR "Hip Prosthesis"[MeSH Terms] OR "Knee Prosthesis"[MeSH Terms] OR "Spinal Fusion"[MeSH Terms] OR "Bone Plates"[MeSH Terms] OR "Joint Prosthesis"[MeSH Terms] OR "ventilators, mechanical"[MeSH Terms] OR "Heart-Lung Machine"[MeSH Terms] OR "Extracorporeal Membrane Oxygenation"[MeSH Terms] OR "Renal Replacement Therapy"[MeSH Terms] OR "Dialysis"[MeSH Terms] OR "surgery, computer assisted"[MeSH Terms] OR "Robotic Surgical Procedures"[MeSH Terms] OR "Laser Therapy"[MeSH Terms] OR "Catheter Ablation"[MeSH Terms] OR "Surgical Staplers"[MeSH Terms] OR "Magnetic Resonance Imaging"[MeSH Terms] OR "tomography, x ray computed"[MeSH Terms] OR "Nuclear Medicine"[MeSH Terms] OR "Positron-Emission Tomography"[MeSH Terms] OR "tomography, emission computed, single photon"[MeSH Terms] OR "Radiotherapy"[MeSH Terms] OR "Brachytherapy"[MeSH Terms] OR "Insulin Infusion Systems"[MeSH Terms] OR "Drug-Eluting Stents"[MeSH Terms] OR "Electric Stimulation Therapy"[MeSH Terms] OR "Pain Management"[MeSH Terms] OR "Cochlear Implants"[MeSH Terms])) AND ("regulatory"[Title] OR "approv*"[Title] OR "certific*"[Title] OR "market"[Title] or "CE-mark*"[Title]) AND 2017/01/01:2025/12/31[Date - Publication]) OR ("device approval"[MeSH Major Topic] AND 2017/01/01:2025/12/31[Date - Publication]) OR ("technology assessment, biomedical"[MeSH Major Topic] AND 2017/01/01:2025/12/31[Date - Publication])) AND (2017:2025[pdat])

III. Scopus:

((ABS ((("innovative" OR "novel" OR "emerging" OR "advanced" OR "next generation") W/3 ("medical device*" OR "healthcare device*" OR "health care device*" OR "therapeutic device*" OR "diagnostic device*" OR "medical equipment*" OR "medical technolog*" OR "clinical device*" OR "biomedical device*" OR "medical product*" OR "medical instrument*" OR "health monitoring device*" OR "patient monitoring device*")) OR ("digital health device*" OR "digital health tool*" OR "digital healthcare device*" OR "digital healthcare tool*" OR "connected medical device*" OR "wireless medical device*" OR "wearable medical device*" OR "point of care device*" OR "remote patient monitoring device*" OR "medical sensor device*" OR "medical robot*" OR "surgical robot*" OR "medical AI device*" OR "smart medical device*" OR "portable medical diagnostic*" OR "AI-enabled medical device*" OR "telehealth device*" OR "telemedicine device*" OR "telerehabilitat*" OR "SaMD") OR ("medical organ-on-chip" OR "medical lab-on-chip" OR "medical 3D print*" OR "medical bioprint*" OR "medical additive manufactur*" OR "medical nanodevice*" OR "medical nanoscale device*" OR "regenerative medicine device*" OR "tissue engineering device*") OR (("minimally invasive" OR "robotic assisted") W/3 ("medical device*" OR "surgical device*" OR "diagnostic device*" OR "therapeutic device*")) OR (medical W/2 software) OR (ai W/2 software W/2 medical))) OR (ABS ((("medical device*" OR "medical product*" OR "healthcare device*" OR "health care device*" OR "therapeutic device*" OR "diagnostic device*" OR "medical equipment*" OR "medical technolog*" OR "medical apparatus*" OR "clinical device*" OR "biomedical device*") W/3 (high-risk OR "high risk" OR "highest risk" OR "class III" OR "class 3" OR "class D" OR "risk class III" OR "critical" OR "life-support*" OR "life support*" OR "life sustain*" OR "life-sustain*" OR "safety critical" OR "safety-critical" OR "high hazard" OR "high-hazard" OR "high concern" OR "high-concern" OR "significant risk" OR implantable OR invasive OR "life threatening" OR "life-threatening" OR "critical care" OR "major surgical" OR "high complexity" OR "high-complexity" OR "substantial risk" OR "serious risk" OR "serious adverse" OR "severe risk" OR "maximum risk" OR "elevated risk" OR "priority device*")) OR (

"implantable device*" OR "class IIb" OR "active implant*" OR "implantable medical*" OR "class III device*" OR "class 3 device*" OR "class D device*" OR "critical medical device*" OR "critical care device*" OR "life support device*" OR "life-sustaining device*" OR "high risk implant*" OR "high-risk medical*" OR "highest risk device*") OR (("in vitro" OR "in-vitro" OR ivd) W/1 (device* OR diagnostic* OR instrument* OR equipment* OR kit* OR reagent* OR calibrator* OR instrument* OR apparat* OR software OR system)) OR (("laboratory diagnostic*" OR "clinical diagnostic*") W/2 (device* OR diagnostic* OR instrument* OR equipment*) W/3 (high-risk OR "high risk" OR "class D" OR "highest risk" OR critical OR "serious public health threat" OR "significant public health threat")) OR ((cardiovascular OR radiation OR neurological OR neurosurgical OR orthopedic OR cardiac OR brain OR spinal OR neural OR "drug delivery" OR "life support") W/3 (implant* OR device* OR equipment*) W/3 (high-risk OR "high risk" OR "class III" OR critical))))) AND (TITLE (regulatory OR proposal OR regulations OR market OR evidence OR "clinical investigat*" OR "clinical trial*" OR ((quality OR transparency OR gap* OR strength OR "real-world" OR "life-cycle" OR generating OR "long-term") W/2 (evidence OR data)) OR registr* OR surveillance OR premarket OR "pre-market" OR postmarket OR pms OR "post-market" OR "post market" OR fda OR "US Food and Drug Administration" OR approval* OR "CE-mark*" OR approved OR classification OR certific* OR recommendations OR guidelines OR "European Union Medical Devices" OR "EU Medical Device Regulation" OR mdr OR ivdr OR "EU In Vitro Diagnostic Medical Device Regulation" OR "ISO standards" OR "life-cycle" OR "health technology assessment" OR hta OR framework OR landscape OR statements OR opportunities OR threats OR methodologies OR testing OR report* OR benefit* OR cost* OR "benefit-risk" OR studies OR safety OR quality OR measures OR efficacy OR legislat* OR standards OR approval OR effectiveness OR safety OR authorisation OR panel OR "meta-analysis" OR review OR "combined studies" OR "bridging studies" OR "cross-sectional" OR retrospective OR comment)) AND PUBYEAR > 2016 AND PUBYEAR < 2026

IV. CORDIS:

EU projects:

Search terms: 'medical' 'device' 'regulation' 'digital health'
Filters:
- Collection: Projects
- Framework Programme: Horizon Europe
- Framework Programme: Horizon 2020
- Language: English

Query: contenttype='project' AND frameworkProgramme='HORIZON','H2020' AND language='en' AND ('medical' AND 'device' AND 'regulation')

EU projects results:

Search terms: 'medical' 'device' 'regulation' 'digital health'
Filters:
- Collection: Results Packs
- Framework Programme: Horizon Europe
- Framework Programme: Horizon 2020
- Language: English

Query: /article/relations/categories/collection/code='resultsPack' AND frameworkProgramme='HORIZON','H2020' AND language='en' AND ('medical' AND 'device' AND 'regulation')

Targeted journals with a focus on Health Technology Assessment.

1. International Journal of Technology Assessment in Health Care
2. Expert Review of Medical Devices
3. Medical Devices: Evidence and Research
4. Therapeutic Innovation & Regulatory Science
5. Expert Review of Pharmacoeconomics & Outcomes Research
6. Journal of Medical Devices-Transactions of the ASME
7. Frontiers in Medical Technology
8. HEALTH TECHNOLOGY ASSESSMENT
9. Global & Regional Health Technology Assessment
10. Value in Health
