# Supplementary material for: Methodologies for generating and evaluating clinical and performance evidence for high-risk and innovative medical devices and in vitro diagnostics: a scoping review
Source: Front Med Technol. 2026 Jun 24;8:1857401. doi: 10.3389/fmedt.2026.1857401 (PMC13341693; doi:10.3389/fmedt.2026.1857401)
Supplement: Supplementary file 2 [file Datasheet2.pdf]

Methodologies for Generating and Evaluating Clinical and Performance Evidence for High-Risk and Innovative Medical Devices and In Vitro Diagnostics: A Scoping Review

Prompts used in pilot testing

Study designs

"Please review the following scientific article and provide a summary focusing strictly on what the article states about study designs used or recommended for the generation and evaluation of clinical and performance evidence for high-risk or innovative medical devices (MDs) and in vitro diagnostic devices (IVDs). The summary must be based solely on the content explicitly presented in the article and should not include any interpretations, assumptions, or conclusions beyond what is directly stated. Include only information that directly discusses types of study designs, their methodological characteristics, intended use, and any advantages, limitations, or regulatory relevance highlighted in the article. Be precise, concise, and stay strictly within the scope of study design methodology as described in the article. Write in a connected narrative format, not as bullet points."

Use of real-world evidence

"Review the following scientific article and summarise what it explicitly states about the use of real-world evidence (RWE) for the generation and evaluation of clinical and performance evidence to support regulatory requirements for high-risk or innovative medical devices (MDs) and in vitro diagnostic devices (IVDs). Focus on how RWE is used or recommended for demonstrating safety, performance, or effectiveness, either as a complement to or substitute for traditional clinical trials. Describe data sources (e.g., electronic health records, claims data, registries, patient-reported outcomes) and methodologies (e.g., pragmatic trials, observational studies, data linkage techniques) discussed in the article. Mention regulatory perspectives or acceptance of RWE, as well as any limitations, biases, or quality concerns that are raised. Do not include any information that is not explicitly about real-world evidence. The summary must be based only on what is clearly stated in the article, without interpretation or expansion. Write clearly, concisely, and with a focus on how RWE contributes to regulatory decision-making and evidence generation strategies. Write in a connected narrative format, not as bullet points."

Use of registries

"Review the following scientific article and summarise what it explicitly states about the use of registries for the generation and evaluation of clinical and performance evidence to meet regulatory requirements for high-risk or innovative medical devices (MDs) and in vitro diagnostic devices (IVDs). Focus on the types of registries discussed (e.g., national, disease-specific, product-specific), their role in supporting pre-market or post-market evidence, and how data from registries is used for regulatory purposes. Describe any methodologies or practices recommended for ensuring data quality, completeness, and regulatory relevance. If relevant, include mention of interoperability, linkage with other data sources, long-term follow-up, or patient outcomes. Highlight the benefits and limitations of using registries in this context, especially regarding regulatory acceptance. Do not include any information that is not explicitly about registries. Write clearly, concisely, and with a focus on how

registries contribute to regulatory decision-making and evidence generation strategies. Write in a connected narrative format, not as bullet points."

Post-market surveillance

"Review the following scientific article and summarise what it explicitly states about post-market surveillance (PMS) strategies and methodologies for the generation and evaluation of clinical and performance evidence to fulfil regulatory requirements for high-risk or innovative medical devices (MDs) and in vitro diagnostic devices (IVDs). Focus on types of PMS activities discussed (e.g., post-market clinical follow-up studies, active surveillance, registry-based monitoring, user feedback systems), their methodological characteristics, and their role in detecting safety or performance issues after market entry. Highlight any recommendations for best practices, risk-based approaches, or lifecycle integration of PMS. Mention challenges such as data quality, underreporting, or integration with other regulatory processes. If applicable, note how PMS findings are used to update risk-benefit assessments or inform regulatory decisions. Do not include any information that is not explicitly about PMS. Write clearly, concisely, and with a focus on how PMS contribute to regulatory decision-making and evidence generation strategies. Write in a connected narrative format, not as bullet points."

Life-cycle assessment

"Review the following scientific article and summarise what it explicitly states about life-cycle assessment (LCA) approaches in the context of clinical and performance evidence generation and evaluation for regulatory purposes related to high-risk or innovative medical devices (MDs) and in vitro diagnostic devices (IVDs). Focus on how the article discusses the integration of evidence across the product lifecycle, from design and development, through clinical investigation and market approval, to post-market surveillance. Describe any frameworks, models, or strategies mentioned for ensuring continuity and consistency of evidence generation throughout the lifecycle. Highlight how LCA is used to inform benefit-risk assessment, regulatory decision-making, or continuous improvement. If relevant, mention the role of adaptive regulatory pathways, iterative evidence development, or dynamic assessment models. Do not include any information that is not explicitly about LCA. Write clearly, concisely, and with a focus on how life-cycle assessment contributes to regulatory decision-making and evidence generation strategies across the product lifespan. Write in a connected narrative format, not as bullet points."

OTHER CATEGORIES:

1) *Synthetic data*

"Review the following scientific article and summarise what it explicitly states about the use of synthetic data in the context of generating or evaluating clinical and performance evidence for high-risk or innovative medical devices (MDs) or in vitro diagnostic devices (IVDs). Focus on how synthetic data are described, proposed, or used to support regulatory requirements, including their methodological roles, intended applications, and any stated advantages, limitations, or regulatory considerations. Do not include any information not directly related to synthetic data. The summary must be based solely on what is stated in the article, without interpretation or extrapolation. Write clearly, concisely, and with a focus on how synthetic data contribute to regulatory decision-making

and strategies for generating and evaluating clinical and performance evidence. Write in a connected narrative format, not as bullet points."

Modelling

"Review the following scientific article and summarise what it explicitly states about the use of modelling in the context of generating or evaluating clinical and performance evidence for high-risk or innovative medical devices (MDs) or in vitro diagnostic devices (IVDs). Focus on how modelling approaches are used or recommended to support regulatory requirements. Describe the type of models discussed, their intended purpose, methodological features, and any limitations or regulatory considerations mentioned in the article. The summary must be based solely on what is stated in the article, without interpretation or extrapolation. Do not include any information that is not directly related to modelling. Write clearly, concisely, and with a focus on how modelling contributes to regulatory decision-making and evidence generation strategies. Write in a connected narrative format, not as bullet points."

Specific methods for evidence generation

"Review the following scientific article and summarise what it explicitly states about specific methods used for generating clinical and/or performance evidence to meet regulatory requirements for high-risk or innovative medical devices (MDs) or in vitro diagnostic devices (IVDs). Focus on methodological techniques, strategies, or structured approaches. Describe their purpose, application, and any stated advantages, limitations, or regulatory implications. The summary must be based solely on what is stated in the article, without interpretation or extrapolation. Do not include any information that is not directly related to specific methods for generating clinical or performance evidence. Write clearly, concisely, and with a focus on how specific evidence generation methods contribute to regulatory decision-making. Write in a connected narrative format, not as bullet points."

Simulation studies

"Review the following scientific article and summarise what it explicitly states about the use of simulation studies in the context of generating or evaluating clinical and/or performance evidence for high-risk or innovative medical devices (MDs) or in vitro diagnostic devices (IVDs). Focus on how simulation studies are used or recommended to support regulatory requirements. Describe the purpose, structure, methodological characteristics, and any stated advantages, limitations, or regulatory considerations related to simulation-based approaches. The summary must be based solely on what is stated in the article, without interpretation or extrapolation. Do not include any information that is not directly related to simulation studies. Write clearly, concisely, and with a focus on how simulation studies contribute to regulatory decision-making and evidence generation strategies. Write in a connected narrative format, not as bullet points."

Methods for AI

"Review the following scientific article and summarise what it explicitly states about methods for artificial intelligence (AI) in the context of generating or evaluating clinical and/or performance evidence for high-risk or innovative medical devices (MDs) or in vitro diagnostic devices (IVDs). Focus on methodological aspects specific to AI. Describe how these methods are applied or recommended to support regulatory compliance and decision-making. Describe the purpose, structure, methodological characteristics, and any stated advantages, limitations, or regulatory considerations related to methods for AI. The summary must be based solely on what is stated in the article, without interpretation or extrapolation. Do not include any information that is not directly related to AI-specific

methods for evidence generation or evaluation. Write clearly, concisely, and with a focus on how AI methods contribute to regulatory strategies and clinical or performance evidence generation. Write in a connected narrative format, not as bullet points."

Mapping and synthesis prompts

Study designs

"You are given a collection of excerpted texts from multiple scientific studies, each labelled by Study ID (such as "Study ID #4"). All excerpts relate specifically to study designs used or recommended for the generation and evaluation of clinical and/or performance evidence in the context of fulfilling regulatory requirements for high-risk or innovative medical devices (MDs) and in vitro diagnostic devices (IVDs).

Your task is to synthesise this material into a comprehensive, structured, and analytical report addressing the following:

Overview of study designs: Identify and describe the types of study designs discussed across the studies. Clarify their methodological characteristics and in what contexts or stages of the device lifecycle they are used or recommended.

Purpose and application in regulatory context: Explain the intended role of these study designs in generating evidence required for regulatory submissions or compliance. Describe how they are used to demonstrate safety, performance, effectiveness, or benefit-risk profiles.

Strengths, limitations, and challenges: Summarise any advantages, constraints, or methodological challenges associated with different study designs, as reported in the included texts.

Regulatory considerations: Present any explicit discussion of how study designs are addressed by regulators, including references to MDR or IVDR requirements, and any expectations, flexibilities, or gaps noted in relation to study design selection or implementation.

Variability and gaps across the literature: Identify any inconsistencies, variation in terminology or application, conflicting perspectives, or underexplored areas related to study designs across the included texts.

☑ *Write in a clear academic narrative, using appropriate headings and subheadings.*

🚫 *Do not summarise each study separately. Focus on synthesising content thematically.*

🚫 *Do not include any information that is not explicitly present in the source texts."*

Use of real-world evidence

"You are given a collection of excerpted texts from multiple scientific studies, each labelled by Study ID (such as "Study ID #4"). All excerpts relate specifically to the use of real-world evidence (RWE) for the generation and evaluation of clinical and/or performance evidence in the context of fulfilling regulatory requirements for high-risk or innovative medical devices (MDs) and in vitro diagnostic devices (IVDs).

Your task is to synthesise this material into a comprehensive, structured, and analytical report addressing the following:

Overview of real-world evidence use: Identify and describe how real-world evidence is used or recommended across the studies. Explain the types of RWE sources mentioned (such as registries, electronic health records, claims data, patient-reported outcomes) and how they contribute to evidence generation.

Purpose and application in regulatory context: Explain the role of RWE in demonstrating safety, performance, effectiveness, or benefit-risk profiles. Describe how it is applied to support regulatory submissions or compliance, including any mention of when it is used as a complement or substitute for traditional study designs.

Strengths, limitations, and challenges: Summarise any advantages, limitations, or concerns reported in relation to the use of RWE, such as issues of data quality, bias, generalizability, or completeness.

Regulatory considerations: Present any explicit statements regarding the acceptance, role, or limitations of RWE within regulatory frameworks, including references to MDR or IVDR, and any guidance or expectations noted.

Variability and gaps across the literature: Identify any inconsistencies, conflicting interpretations, or underexplored issues regarding the use of RWE in the studies.

☑ *Write in a clear academic narrative, using appropriate headings and subheadings.*

🚫 *Do not summarise each study separately. Focus on synthesising content thematically.*

🚫 *Do not include any information that is not explicitly present in the source texts."*

Use of registries

"You are given a collection of excerpted texts from multiple scientific studies, each labelled by Study ID (such as "Study ID #4"). All excerpts relate specifically to the use of registries in the generation and evaluation of clinical and/or performance evidence in the context of fulfilling regulatory requirements for high-risk or innovative medical devices (MDs) and in vitro diagnostic devices (IVDs).

Your task is to synthesise this material into a comprehensive, structured, and analytical report addressing the following:

Overview of registry use: Identify and describe how registries are used or recommended across the studies. Include the types of registries mentioned (clinical, disease-specific, product-specific, national, European, etc.) and the role they play in evidence generation and evaluation.

Purpose and application in regulatory context: Explain how registry-based data is used to support regulatory submissions, including its role in demonstrating safety, performance, long-term effectiveness, or benefit-risk profiles.

Strengths, limitations, and challenges: Summarise the reported benefits and challenges of using registries, such as strengths in longitudinal follow-up, real-world representativeness, or regulatory relevance, and limitations related to data quality, completeness, standardisation, or access.

Regulatory considerations: Present any references to regulatory expectations, guidance, or frameworks that support or limit the use of registries in compliance with MDR or IVDR.

Variability and gaps across the literature: Identify any inconsistencies, conflicting positions, or underexplored areas related to the use of registries.

☑ *Write in a clear academic narrative, using appropriate headings and subheadings.*

🚫 *Do not summarise each study separately. Focus on synthesising content thematically.*

🚫 *Do not include any information that is not explicitly present in the source texts."*

Post-market surveillance

"You are given a collection of excerpted texts from multiple scientific studies, each labelled by Study ID (such as "Study ID #4"). All excerpts relate specifically to post-market surveillance (PMS) activities in the context of generating or evaluating clinical and/or performance evidence for high-risk or innovative medical devices (MDs) and in vitro diagnostic devices (IVDs), as part of fulfilling regulatory requirements.

Your task is to synthesise this material into a comprehensive, structured, and analytical report addressing the following:

Overview of post-market surveillance approaches: Identify and describe the various post-market surveillance methods or systems discussed across the studies. Include any references to active or passive surveillance, structured follow-up studies, registries, real-world monitoring systems, or manufacturer-led initiatives.

Purpose and application in regulatory context: Explain the role of post-market surveillance in generating evidence on long-term safety, performance, effectiveness, or emerging risks. Describe how these approaches are applied to fulfil MDR or IVDR obligations or to inform benefit-risk reassessment.

Strengths, limitations, and challenges: Summarise any benefits, limitations, or methodological concerns related to PMS, such as timeliness, data completeness, underreporting, integration with pre-market data, or coordination across jurisdictions.

Regulatory considerations: Present any statements regarding the regulatory expectations or guidance concerning PMS, including requirements under MDR or IVDR and how these shape methodological choices.

Variability and gaps across the literature: Identify any inconsistencies, differences in reporting depth, or missing perspectives across the included texts related to PMS practices or requirements.

☑ *Write in a clear academic narrative, using appropriate headings and subheadings.*

🚫 *Do not summarise each study separately. Focus on synthesising content thematically.*

🚫 *Do not include any information that is not explicitly present in the source texts."*

Life-cycle assessment

"You are given a collection of excerpted texts from multiple scientific studies, each labelled by Study ID (such as "Study ID #4"). All excerpts relate specifically to life-cycle assessment (LCA) or life-cycle-based approaches to the generation or evaluation of clinical and/or performance evidence for high-risk or

innovative medical devices (MDs) and in vitro diagnostic devices (IVDs), in the context of fulfilling regulatory requirements.

Your task is to synthesise this material into a comprehensive, structured, and analytical report addressing the following:

Overview of life-cycle approaches: Identify and describe how life-cycle thinking or life-cycle assessment is defined and applied across the studies. Include whether LCA refers to clinical development stages (pre-market, market access, post-market), environmental impact assessments, or evidence planning across the product lifespan.

Purpose and application in regulatory context: Explain the role of life-cycle-based approaches in guiding evidence generation, long-term planning, or regulatory compliance. Describe how life-cycle thinking supports regulatory decisions, post-market obligations, or adaptive evidence strategies.

Strengths, limitations, and challenges: Summarise any advantages or limitations associated with applying life-cycle concepts to medical device or IVD evidence development, such as complexity of coordination, long-term data requirements, integration across data sources, or regulatory feasibility.

Regulatory considerations: Present any references to MDR or IVDR requirements, expectations, or guidance that relate to life-cycle-based evidence generation or structured approaches to benefit-risk reassessment over time.

Variability and gaps across the literature: Identify any inconsistencies, differences in terminology, underdeveloped areas, or gaps in the practical application of life-cycle thinking across the included studies.

☑ *Write in a clear academic narrative, using appropriate headings and subheadings.*

🚫 *Do not summarise each study separately. Focus on synthesising content thematically.*

🚫 *Do not include any information that is not explicitly present in the source texts."*

Other topics

"You are given a collection of excerpted texts from multiple scientific studies, each labelled by Study ID (such as "Study ID #4"). These excerpts address methodological elements or approaches related to the generation and evaluation of clinical and/or performance evidence for high-risk or innovative medical devices (MDs) and in vitro diagnostic devices (IVDs).

Your task is to synthesise this material into a comprehensive, structured, and analytical report addressing the following:

Overview of methods and themes: Identify and describe the types of methods, strategies, or concepts discussed in these studies that relate to evidence generation or evaluation, but which are not classified under the main categories (study design, evidence comparison, integration, real-world data, registries, post-market surveillance, or life-cycle assessment). These may include novel methodological proposals, specific tools, validation frameworks, expert consensus strategies, data quality assurance mechanisms, or any other relevant approaches.

Purpose and application in regulatory context: Clarify the intended role or value of these methods in the context of regulatory requirements. Describe how they contribute to demonstrating safety, performance, effectiveness, or benefit-risk balance of MDs or IVDs.

Strengths, limitations, and challenges: Summarise the advantages, limitations, or open questions related to these methods as discussed in the texts.

Regulatory considerations: Include any references to how these approaches are viewed by regulators or situated within the regulatory frameworks, such as MDR or IVDR.

Variability and gaps across the literature: Identify any inconsistencies, emerging trends, or underexplored areas found in this material that suggest directions for further research or clarification.

☑ *Write in a clear academic narrative, using appropriate headings and subheadings.*

🚫 *Do not summarise each study separately. Focus on synthesising content thematically.*

🚫 *Do not include any information that is not explicitly present in the source texts."*
