# Supplementary material for: Methodologies for generating and evaluating clinical and performance evidence for high-risk and innovative medical devices and in vitro diagnostics: a scoping review
Source: Front Med Technol. 2026 Jun 24;8:1857401. doi: 10.3389/fmedt.2026.1857401 (PMC13341693; doi:10.3389/fmedt.2026.1857401)
Supplement: Supplementary file 3 [file Table1.docx]

**Supplementary Table 1** Characteristics of studies included in the scoping review

|  | **Author(s) and Year** | **Title** | **DOI** | **Key Findings** |
| --- | --- | --- | --- | --- |
| 1 | Alloza C et al., 2023 | A Case for Synthetic Data in Regulatory Decision-Making in Europe. | 10.1002/cpt.3001 | Synthetic data can address key limitations of RWD in Europe, particularly issues related to privacy, restricted access, fragmentation, and delays in data sharing. It enables secure cross-border data exchange and can increase sample sizes, including representation of rare diseases and underrepresented populations, thereby improving the robustness of analyses. Evidence shows that synthetic data can achieve high validity and reproduce analytical results comparable to original datasets when appropriate generation and validation methods are applied. However, challenges remain regarding the need for high-quality source data, computational resources, and the absence of standardized validation metrics and methodological frameworks. The lack of clear regulatory guidelines and consensus on acceptable use currently limits its integration into regulatory decision-making processes. |
| 2 | Baumfeld Andre E et al., 2022 | The Current Landscape and Emerging Applications for RWD in Diagnostics and Clinical Decision Support and its Impact on Regulatory Decision Making | 10.1002/cpt.2565 | RWD are increasingly used to support regulatory decision-making across the lifecycle of medical products, including IVDs and CDSS. RWD can provide evidence on test performance, clinical outcomes, and benefit–risk profiles, and has already been used in regulatory submissions through sources such as electronic health records, registries, and retrospective analyses. In diagnostics, RWD can supplement or replace traditional clinical validation approaches, particularly when randomized trials are not feasible, while in CDSS it supports evaluation of safety, effectiveness, and real-world performance. However, the utility of RWD is limited by issues such as missing data, lack of standardization, variable data quality, and insufficient patient follow-up, which can affect reliability and regulatory acceptance. Additional barriers include restricted data access, privacy regulations, and poor interoperability across systems, highlighting the need for harmonized frameworks, improved data governance, and standardized methodologies to enable consistent use of RWD in regulatory contexts. |
| 3 | Baumfeld Andre E et al., 2024 | The Open Hand Initiative: Facilitating the Use of RWE in Regulatory Submissions Through Collaboration and Transparency | 10.1002/cpt.3539 | The Open Hand Initiative introduces a collaborative and transparent approach between regulators and device manufacturers to improve the use of RWD in regulatory submissions for in vitro diagnostics. This approach enables early dialogue on data collection, study design, and acceptance criteria, helping ensure that RWD is fit-for-purpose and can generate valid RWE. The pilot demonstrated that transparent interactions facilitate identification of key challenges, including inconsistent data quality, limited access to complete patient data, lack of standardized data elements, and difficulties in defining appropriate comparator tests. Rapid changes during the COVID-19 pandemic further affected RWD availability, study populations, and regulatory expectations, complicating evidence generation. Clear alignment with regulatory requirements, predefined data collection strategies, and early engagement with regulators were identified as critical factors for successfully using RWD to support regulatory decision-making. |
| 4 | Bischoff JE et al., 2023 | A risk and credibility framework for in silico clinical trials of MDs | 10.1016/j.cmpb.2023.107813 | ISCTs are emerging as a method to generate clinically relevant data through computational modeling, addressing limitations of traditional clinical studies such as restricted sample sizes and the inability to capture rare scenarios. A risk-based credibility framework is proposed to evaluate ISCT applications using three key factors—scope, coverage, and severity—to determine model risk and required validation rigor. The framework emphasizes the need for both verification and validation, including clinical validation activities that demonstrate alignment between model predictions and real clinical outcomes. Credibility depends on factors such as the quality and representativeness of clinical comparator data, the ability of the model to capture clinical variability, and the level of agreement between simulated and observed outcomes. The approach supports structured assessment of model reliability and is intended to reduce uncertainty in regulatory evaluation, facilitating the integration of ISCTs into MD decision-making. |
| 5 | Blüher M et al., 2019 | Critical Review of European Health-Economic Guidelines for the Health Technology Assessment of MDs | 10.3389/fmed.2019.00278 | European HTA guidelines show substantial variability and limited consensus regarding the evaluation of MDs, particularly in the context of health-economic analysis. Most existing guidelines are primarily designed for pharmaceuticals and often lack device-specific recommendations, with many countries providing only general or minimal guidance. Key challenges identified include weak evidence bases, learning-curve effects, organizational impact, incremental innovation, device diversity, dynamic pricing, and limited transferability of results across settings. Current approaches to economic evaluation vary widely, with cost-effectiveness and cost-utility analyses most commonly recommended, although these may not fully capture the value of MDs. The findings highlight the need for more tailored, flexible, and standardized frameworks that address the specific characteristics of MDs to improve consistency and decision-making in European HTA processes. |
| 6 | Brass I et al, 2022 | Risk Assessment and Classification of MD Software for the Internet of Medical Things Challenges arising from connected, intelligent MDs | 10.1145/3567445.3571104 | CIMDs introduce significant challenges to existing regulatory frameworks due to their dynamic and evolving risk profiles throughout the device lifecycle. Software updates, adaptive algorithms, and cybersecurity vulnerabilities can alter device behavior over time, often without clear visibility for manufacturers, regulators, or healthcare professionals. These changes complicate traditional risk assessment and classification approaches, which are typically based on static, pre-market evaluations and may not reflect real-world performance. Post-market surveillance is also challenged by the difficulty of detecting performance degradation, attributing causes of failure, and monitoring continuous algorithmic changes in complex, interconnected systems. Additional concerns include limited transparency, difficulties in reporting compromised devices, and insufficient regulatory guidance on managing software modifications and AI-driven functionalities, highlighting the need for more adaptive and continuous regulatory approaches. |
| 7 | Brönneke JB et al., 2023 | Dynamic HTA for digital health solutions: opportunities and challenges for patient-centered evaluation | 10.1017/S0266462323002726 | Dynamic HTA is proposed as a more suitable approach for evaluating digital health applications due to their iterative development and continuous updates. Traditional HTA methods based on static evidence are insufficient, as digital products can evolve after market entry and generate new data over time. The German DiGA framework illustrates how reimbursement can occur before full clinical evidence is available, with additional data generated during real-world use to support ongoing evaluation. Flexible study designs and the use of RWD and RWE enable continuous assessment of effectiveness, safety, and value in practice. This approach also allows inclusion of broader patient-centered outcomes, such as improvements in care processes and health literacy, supporting a more comprehensive evaluation of digital health technologies. |
| 8 | Buccheri S et al., 2024 | Proposals for a hierarchy of clinical study designs | N/A | A hierarchy of clinical study designs is proposed to support the evaluation of high-risk MDs, based on the concept of the “pyramid of evidence,” where study designs are ranked according to their risk of bias and validity. RCTs are identified as the highest level of evidence, while observational studies such as cohort and case-control studies provide supportive but less robust evidence. Clinical evaluation is described as a progressive, lifecycle-based process, starting from preclinical studies and early feasibility studies, followed by larger observational studies and ultimately confirmatory RCTs. The selection of study design should depend on the stage of development and the type of device, with more rigorous designs required as the device matures and is compared to existing alternatives. Continuous post-market surveillance, including the use of registries, is essential to monitor long-term safety, performance, and real-world outcomes after market approval. |
| 9 | Burcu M et al., 2021 | A Framework for Extension Studies Using RWD to Examine Long-Term Safety and Effectiveness. | 10.1007/s43441-021-00322-8 | Extension studies using RWD provide a practical approach to evaluate long-term safety, effectiveness, and durability of treatments beyond the duration of traditional clinical trials. These studies allow patients from parent trials to be followed over extended periods through observational designs, often using linked data sources such as registries, electronic health records, or claims databases. RWD-based approaches can reduce costs, minimize patient and site burden, and enable large-scale, long-term follow-up that would be difficult to achieve with conventional trial infrastructure. However, methodological challenges include selection bias, loss to follow-up, variability in outcome measurement, and the need for appropriate comparators and robust analytical strategies. Careful study design, including clear definition of time points, endpoint selection, data linkage, and bias mitigation methods, is essential to ensure valid and reliable long-term evidence generation. |
| 10 | Cafri G et al., 2018 | A review and empirical comparison of causal inference methods for clustered observational data with application to the evaluation of the effectiveness of MDs | 10.1177/0962280218799540 | Observational studies of MDs frequently involve clustered data structures, such as patients nested within surgeons or hospitals, which introduce additional sources of bias beyond standard confounding. Both patient-level and cluster-level confounders, including unmeasured characteristics such as provider experience, can affect treatment effect estimates and must be addressed in study design and analysis. Methods based on propensity scores, including matching and weighting, are commonly used to reduce confounding and estimate causal treatment effects, with marginal models preferred for comparability to randomized studies. Incorporating clustering into both the estimation of treatment effects and variance calculation improves accuracy and reduces bias compared to approaches that ignore clustering. Overall, appropriate handling of clustered observational data requires combining robust causal inference methods with strategies that explicitly account for dependency and cluster-level confounding. |
| 11 | Cai L et al., 2023 | Discuss Application of RWD from the Boao Lecheng Pilot Zone to Support Premarket Clinical Evaluation of MD | 10.3969/j.issn.1671-7104.2023.01.015. | RWD from the Boao Lecheng Pilot Zone can support premarket clinical evaluation of MDs by providing additional clinical evidence under real clinical practice conditions. RWD can be used either as supplementary evidence to address differences between a device and its comparator or in combination with foreign clinical trial data to strengthen the overall evidence base for safety and effectiveness. This approach can reduce the need for redundant clinical trials, particularly for innovative or high-risk devices with limited existing evidence. Study design requires careful consideration of research objectives, data relevance, sample size, endpoints, and statistical methods to ensure that generated evidence is fit-for-purpose. Data quality assessment is critical and should evaluate aspects such as relevance, reliability, completeness, and control of bias to ensure that RWD provides credible support for regulatory decision-making. |
| 12 | Callea G et al., 2022 | Recommendations for the design and implementation of an Early Feasibility Studies program for MDs in the European Union. | 10.1080/17434440.2022.2075729 | EFS are small-scale, early-stage clinical investigations conducted to evaluate initial safety, functionality, and proof of concept of MDs before the final design is established. They allow iterative modifications of the device and clinical protocol during the study, enabling rapid optimization based on early clinical feedback. The implementation of an EFS program can improve the timeliness and quality of clinical evidence generation while increasing the attractiveness of a regulatory system for early-stage innovation. In Europe, the absence of a standardized procedural framework and limited regulatory guidance currently restricts the use of EFS, despite its inclusion in recent regulatory frameworks. Successful implementation requires structured processes, dedicated resources, strong regulatory–developer interaction, and careful consideration of ethical issues, particularly related to higher uncertainty and patient risk. |
| 13 | Cao H et al., 2023 | Bayesian approach for design and analysis of MD trials in the era of modern clinical studies | 10.1515/mr-2023-0026 | Bayesian approaches provide a flexible framework for the design and analysis of MD trials by integrating prior information with newly collected data to improve efficiency and decision-making. These methods allow borrowing of historical data or evidence from previous device versions, reducing required sample sizes and shortening study duration while maintaining robust evaluation of safety and effectiveness. Bayesian adaptive designs enable interim analyses and dynamic modifications, such as sample size adjustments or early stopping decisions, based on accumulating data. The approach is particularly suitable for MDs due to their rapid development cycles, iterative improvements, and availability of real-world and historical data sources. Despite these advantages, challenges include assumptions of data exchangeability, handling heterogeneity across data sources, and the need for careful methodological selection to ensure valid and reliable results. |
| 14 | Chiaruttini MV et al., 2024 | Bayesian Dynamic Borrowing in Group-Sequential Design for MD Studies | 10.1186/s12874-025-02520-6 | Bayesian dynamic borrowing combined with group-sequential design enables efficient integration of historical and current data in MD trials while allowing interim decision-making. The approach dynamically adjusts the weight of historical information based on the level of agreement between past and newly collected data, reducing bias when discrepancies are present. Simulation results show that this method can substantially reduce the number of newly enrolled patients while maintaining control over Type I error and statistical power, particularly in scenarios where historical and current data are consistent. Early stopping rules for efficacy and futility further improve efficiency and ethical conduct by minimizing unnecessary patient exposure and shortening trial duration. When historical data are not aligned with current observations, the method appropriately downweights prior information, preserving the validity and robustness of the analysis. |
| 15 | Cipriani A et al., 2020 | Generating comparative evidence on new drugs and devices after approval | 10.1016/S0140-6736(19)33177-0 | Evidence available at the time of approval for drugs and MDs is often incomplete, with persistent uncertainties regarding comparative effectiveness, safety, and long-term outcomes. Post-marketing research is frequently fragmented, delayed, and methodologically limited, often lacking active comparators, relying on observational designs, and focusing on surrogate rather than patient-relevant outcomes. Randomized controlled trials with appropriate comparators and clinically meaningful endpoints are identified as the most reliable approach to generate robust comparative evidence after approval, although they are underutilized. Non-randomized studies can support evidence generation but are limited by confounding and bias, particularly when estimating treatment effects. Improved coordination across the product lifecycle, along with structured post-marketing evidence generation plans and stronger regulatory oversight, is necessary to address evidence gaps and support informed clinical and policy decision-making. |
| 16 | Damkjær M et al., 2025 | Scoping review on regulation, implementation and postmarket surveillance of MDs. | 10.1371/journal.pone.0325250 | The scoping review identified 139 empirical studies on MD regulation, implementation, and post-market surveillance, with most research focusing on regulatory approval and predominantly using FDA data, while studies from the European context and on implementation processes were limited. Evidence supporting device approval was often inadequate, with frequent use of weak study designs, lack of control groups, short follow-up periods, and reliance on surrogate endpoints, making assessment of safety and effectiveness difficult. Implementation decisions were influenced not only by clinical evidence but also by clinician experience, cost considerations, and interactions with industry, with limited transparency and research on how devices are selected in practice. Post-market surveillance relied heavily on existing databases, which were affected by underreporting, missing data, and variable data quality, limiting their ability to reliably detect safety issues. Overall, the findings indicate a need for stronger evidence requirements, improved data accessibility, and development of high-quality registries to support more reliable evaluation and decision-making across the MD lifecycle. |
| 17 | Daubner-Bendes R et al., 2021 | Quo Vadis HTA for MDs in Central and Eastern Europe? Recommendations to Address Methodological Challenges | 10.3389/fpubh.2020.612410 | HTA of MDs in Central and Eastern Europe is challenged by limited resources, weaker evidence bases, and difficulties in transferring data from other settings. Evidence for MDs is often less robust than for pharmaceuticals, frequently relying on surrogate outcomes, small sample sizes, and limited randomized clinical trials, which increases uncertainty in decision-making. RWE plays a more prominent role but has limited transferability due to differences in patient populations, healthcare systems, and clinical practice, requiring careful adaptation and validation. Additional factors such as learning curves, centre effects, and frequent product modifications further complicate the assessment of clinical and economic value. The study proposes structured approaches, including reuse of international evidence, reliance on RWD, and adaptation of economic models, to improve HTA processes and support evidence-based decision-making in resource-constrained settings. |
| 18 | Davis SE et al., 2023 | Simulating complex patient populations with hierarchical learning effects to support methods development for post-market surveillance | 10.1186/s12874-023-01913-9 | Synthetic data generation provides a controlled environment for developing and validating analytical methods when RWD lack known ground truth, particularly in complex clinical settings. The proposed framework generates realistic synthetic patient populations with correlated features, treatment assignments, and outcomes, while incorporating hierarchical learning effects at both provider and institutional levels. This approach allows simulation of how adverse event risks change over time as experience with new treatments or devices increases, reflecting real-world learning curves and variability. Results demonstrate that simulated datasets closely match specified parameters and reproduce complex patterns such as changing risk over case sequences, although smaller sample sizes introduce greater variability. The framework enables more accurate evaluation of methods for post-market surveillance by distinguishing intrinsic treatment risks from learning-related effects, supporting improved safety monitoring and decision-making. |
| 19 | de Pauvourville G et al., 2022 | RWD and evidence in health technology assessment: When are they complementary, substitutes, or the only sources of data compared to clinical trials? | 10.1016/j.therap.2022.11.001 | RWD plays a variable role in health technology assessment depending on the evaluation objective, product lifecycle stage, and type of technology. RWD complements clinical trials by providing insights into real-world effectiveness, safety, patient pathways, and organizational and economic impacts that cannot be fully captured in controlled trial settings. In certain contexts, such as MDs, rare diseases, or when randomized trials are not feasible, RWD can serve as a substitute source of evidence for assessing benefit–risk profiles. RWD may also represent the only available data source for evaluating contextual factors, healthcare system impact, and real-world utilization patterns across the technology lifecycle. However, their use requires robust methodological approaches to address biases and ensure sufficient validity and acceptability in decision-making processes. |
| 20 | De A and Lohani A, 2025 | Regulatory Adoption of AI, ML, Computational Modeling & Simulation in In-Silico Clinical Trials for MDs: A Systematic Review | 10.1007/s43441-025-00871-2 | ISCTs use computational modelling and simulation, combined with artificial intelligence and machine learning, to evaluate MDs by replicating human physiology and device interactions in virtual environments. These approaches enable the creation of virtual patient cohorts, including synthetic and patient-specific models, allowing assessment of safety, performance, and variability across diverse populations without extensive reliance on human or animal testing. Techniques such as finite element analysis, computational fluid dynamics, and agent-based modelling provide detailed insights into device behaviour, while AI and machine learning improve predictive accuracy, optimize trial design, and support risk identification. Regulatory agencies are increasingly incorporating these methods into evaluation processes through frameworks focused on model credibility, validation, and risk-based assessment, although acceptance is still developing. Despite clear advantages in efficiency, cost reduction, and ethical considerations, adoption is limited by challenges related to data availability, lack of standardization, computational demands, and concerns about transparency and bias, requiring further development of harmonized guidelines and robust validation approaches. |
| 21 | Edelson JB and Rossano JW, 2022 | Pediatric Device Trials are the Ideal way to Bring Devices to Market | 10.1177/21501351221075572 | RCTs are the most reliable method for evaluating the safety and effectiveness of paediatric MDs, as they enable balanced comparison groups and more accurate estimation of treatment effects. Reliance on observational data or clinical experience alone can lead to misleading conclusions, as demonstrated by historical examples where widely accepted practices were later shown to be harmful when tested in trials. Registries provide valuable information on patient characteristics, outcomes, and risk factors, but are limited by missing data, lack of control groups, and inability to support causal inference. Conducting paediatric device trials is challenging due to small populations, ethical constraints, and logistical barriers, but innovative designs such as adaptive trials and composite endpoints can improve feasibility and efficiency. Well-designed prospective trials remain essential for generating robust evidence and supporting evidence-based clinical practice in paediatric populations. |
| 22 | Faris O and Shuren J, 2017 | An FDA Viewpoint on Unique Considerations for Medical-Device Clinical Trials | 10.1056/NEJMra1512592 | MD clinical trials differ substantially from pharmaceutical trials due to the diversity of devices, iterative development processes, and varying risk profiles that influence evidence requirements. High-risk and innovative devices typically require clinical data to demonstrate a favourable benefit–risk balance, while lower-risk devices may rely primarily on bench testing or demonstration of equivalence to existing products. Practical and ethical constraints often limit the feasibility of large, blinded, randomized trials, leading to the use of alternative designs such as single-arm studies, adaptive trials, or reliance on objective performance criteria. Nonclinical data, including engineering tests, modelling, and simulations, can play a central role and may reduce the need for extensive premarket clinical trials, particularly when supported by confirmatory clinical evidence. RWD sources such as registries and post-market studies are essential for ongoing evaluation, supporting label expansions, and addressing residual uncertainty throughout the device lifecycle. |
| 23 | Fleetcroft C et al., 2020 | IDEAL as a guide to designing clinical device studies consistent with the new European MD Regulation | 10.1136/bmjsit-2020-000066 | The EU MDR introduces stricter requirements for clinical evidence, particularly for high-risk and implantable devices, and shifts evaluation toward a lifecycle approach with continuous evidence generation. The regulation requires at least one well-designed clinical study for most devices but does not specify study designs, leaving manufacturers responsible for justifying their methodological choices. The IDEAL framework provides a structured pathway for evidence generation across stages of device development, from early feasibility to long-term surveillance, aligning closely with EU MDR principles. Early-stage studies focus on feasibility, safety, and iterative development, while later stages incorporate comparative studies and long-term RWD collection, often through registries. This alignment suggests that using IDEAL-D can support systematic planning of clinical evidence generation and improve consistency, efficiency, and regulatory compliance throughout the device lifecycle. |
| 24 | Fleurence et al., 2019 | The Future of Registries in the Era of RWE for MDs | 10.1001/jamacardio.2018.4933 | Device registries have historically been a key source of clinical evidence for MDs, supporting research, safety surveillance, quality improvement, and regulatory decision-making through the structured collection of patient-level data. Advances in digitization and standardization of health data have enabled the expansion of RWD use, allowing integration of electronic health records, claims data, and patient-generated data to enhance evidence generation. Centralized registries provide high-quality, detailed data and established methodologies, but face challenges related to sustainability, data completeness, and governance of data use. Decentralized network models, such as PCORnet and NEST, offer broader data coverage and larger sample sizes by linking multiple health systems, enabling studies of rare events and diverse populations, although they often lack data depth and require additional data harmonization. Combining registry-based approaches with network models and embedding randomized studies within these systems can improve efficiency, scalability, and timeliness of RWE generation for MDs. |
| 25 | Fluerence RL and Shuren J, 2018 | Advances in the Use of RWE for MDs: An Update From the National Evaluation System for Health Technology | 10.1002/cpt.1380 | RWE is increasingly used to support regulatory decision-making for MDs across the entire product lifecycle, including premarket evaluation, post-market surveillance, and label expansion. NEST was established as a multistakeholder initiative to coordinate the use of RWD from sources such as electronic health records, claims data, and registries to generate robust clinical evidence. RWE offers opportunities to address limitations of traditional trials, particularly in the context of rapidly evolving devices, operator-dependent outcomes, and challenges with blinding and randomization. NEST demonstrates the feasibility of using both retrospective and prospective data to support regulatory submissions and active safety surveillance, including the detection of safety signals using large, linked datasets. However, challenges remain related to data quality, standardization, methodological rigor, and integration of heterogeneous data sources, requiring the development of frameworks and expert oversight to ensure reliability and regulatory acceptance. |
| 26 | Fraser AG et al., 2021 | Improved clinical investigation and evaluation of high-risk MDs: the rationale and objectives of CORE-MD (Coordinating Research and Evidence for MDs) | 10.1093/ehjqcco/qcab059 | The European regulatory system for high-risk MDs faces significant challenges due to limited methodological guidance and variability in the clinical evidence used for market approval. The EU MDR strengthens requirements for clinical data but provides only general principles, leading to inconsistencies in study design, evidence quality, and interpretation across notified bodies. The CORE–MD project aims to address these gaps by developing standardized methodologies for clinical investigations, including improved study designs, statistical approaches, and integration of patient-reported outcomes. Particular challenges in device evaluation include iterative development, learning curves, difficulties with randomization and blinding, and reliance on both pre- and post-market data sources such as registries and RWE. The project emphasizes the need for transparent, rigorous, and harmonized approaches to evidence generation, including the use of registries, pragmatic trials, and novel methodologies, to improve safety, effectiveness assessment, and regulatory decision-making for high-risk devices. |
| 27 | Fraser AG et al., 2024 | Recommendations for a hierarchy of clinical evidence for high-risk MDs | N/A | A hierarchy of clinical evidence for high-risk MDs is proposed, but its application depends on the stage of device development and clinical context rather than a fixed ranking of study designs. Evidence generation follows a lifecycle approach with four stages—initial, early, definitive, and long-term—each requiring different study designs, ranging from small feasibility studies and case series to RCTs and post-market registry-based studies. RCTs are identified as the optimal design for confirming safety and effectiveness, but are underutilized, with most devices entering the market based on limited or non-randomized evidence. High-quality study design is defined by factors such as sufficient statistical power, minimization of bias, appropriate comparators, complete follow-up, and transparent reporting, while well-designed observational or registry studies may provide valuable evidence when RCTs are not feasible. For innovative or orphan devices, earlier approval based on limited evidence may be acceptable if complemented by mandatory post-market studies, ensuring continuous evidence generation and long-term monitoring of safety and performance. |
| 28 | Fraser AG et al., 2025 | Recommended methodologies for clinical investigations of high-risk MDs—Conclusions from the European Union CORE–MD Project | 10.1016/j.lanepe.2025.101460 | The paper provides consensus recommendations from the CORE–MD project on appropriate methodologies for clinical investigations of high-risk MDs, highlighting the need for stronger and more consistent evidence before approval. Clinical evaluation should follow a structured four-stage approach—initial, early, confirmatory, and long-term studies—covering the full device lifecycle from first-in-human use to post-market follow-up. RCTs are recommended as the preferred design for confirmatory studies, ideally comparing devices with the best available treatment or, when appropriate, sham interventions, while well-designed observational studies can provide supportive evidence when randomization is not feasible. The framework emphasizes the importance of transparency, predefined protocols, adequate statistical power, patient-relevant outcomes, and long-term follow-up through registries and RWD to assess safety and effectiveness over time. Flexible regulatory pathways may allow earlier approval for breakthrough or orphan devices with limited evidence, but only with mandatory post-market studies to confirm clinical benefit and ensure ongoing monitoring of risks. |
| 29 | Freyer O et al., 2024 | Methodologies for the benefit–risk analysis of MDs: A systematic review | 10.21203/rs.3.rs-4832842/v1 | Benefit–risk analysis is a mandatory component of MD evaluation, required by regulatory frameworks such as the EU MDR and US FDA, but there is no standardized method for how it should be conducted. Multiple methodologies exist, including both qualitative and quantitative approaches such as multicriteria decision analysis, net benefit scoring, quantitative modelling, and the FDA’s benefit–risk framework, each differing in structure, objectivity, and data requirements. Quantitative methods offer more structured and transparent assessment by numerically comparing benefits and risks, but still involve subjectivity in selecting endpoints and assigning weights, while qualitative methods remain widely used despite concerns about bias and lack of consistency. MDs present specific challenges for BRA due to complex use contexts, heterogeneous patient populations, learning effects, and difficulties in quantifying certain risks, such as those related to software, AI, or usability. Combining qualitative and quantitative elements is suggested as a more comprehensive approach to capture both measurable outcomes and context-specific factors in regulatory decision-making. |
| 30 | Freyer O et al., 2025 | Methodologies for the benefit–risk analysis of MDs: a systematic review | 10.1080/17434440.2025.2517168 | The systematic review identified 16 different methodologies for benefit–risk analysis of MDs, highlighting substantial variability in approaches and a lack of a universally accepted standard. Methods range from qualitative frameworks, such as regulatory guidance-based approaches, to quantitative models that calculate benefit–risk ratios or aggregate scores using predefined criteria and weights. Quantitative approaches are often considered more structured and transparent, but they still rely on subjective decisions when defining endpoints and assigning importance to benefits and risks, limiting their objectivity. Many methods are adapted from the pharmaceutical field and may not fully capture the complexity of MDs, including diverse use cases, multiple stakeholders, and challenges in quantifying certain risks such as usability or emerging technological features. Overall, existing methodologies are often insufficient to fully support consistent and reliable decision-making, indicating the need for more standardized, flexible, and device-specific approaches to benefit–risk evaluation. |
| 31 | Gomes M et al., 2024 | Acceptability of Using RWD to Estimate Relative Treatment Effects in Health Technology Assessments: Barriers and Future Steps | 10.1016/j.jval.2024.01.020 | Nonrandomized studies using RWD are increasingly used in HTA to estimate comparative treatment effects when RCTs are not feasible or provide limited evidence. These studies can complement trial data by capturing long-term outcomes, real-world effectiveness, and effects in broader patient populations, but their acceptance remains limited due to concerns about bias, data quality, and methodological rigor. Key barriers include residual confounding, inconsistent or incomplete methodological guidelines, limited access to high-quality and timely data, and variability in how best practices are implemented by evidence generators. Even when high-quality nonrandomized studies are conducted, acceptance is further constrained by uncertainty related to unmeasured biases and limited expertise or institutional capacity within HTA bodies to evaluate such evidence. Improving acceptability requires more detailed and harmonized guidelines, better data infrastructure and access, wider use of advanced causal inference methods, and demonstration projects that validate nonrandomized studies against randomized evidence and support decision-making under uncertainty. |
| 32 | Goswami S et al., 2025 | The evolving role of RWE in MD approvals in the United States | 10.1080/17434440.2025.2498458 | The use of RWE in MD approvals in the United States is increasing, expanding beyond its traditional role in post-market surveillance to support premarket approval applications and regulatory decision-making. RWE, derived from sources such as electronic health records, claims data, registries, and wearable technologies, complements clinical trials by providing insights into long-term safety, effectiveness, and performance across broader and more diverse patient populations. However, its integration into regulatory processes is challenged by issues related to data quality, inconsistency, missing data, and variability across sources, as well as the need for rigorous validation, standardized data collection, and robust study design to ensure reliability and generalizability. Additional concerns include bias and confounding inherent in observational data, lack of clear methodological guidance, and the need to balance data access with privacy and security requirements. Despite these limitations, increasing regulatory support and stakeholder collaboration, along with the development of clearer guidelines and hybrid approaches combining RWE with randomized clinical trials, are expected to strengthen its role in MD evaluation and approval processes. |
| 33 | Graili P et al., 2023 | Integration of RWE from different data sources in health technology assessment | 10.3389/jpps.2023.11460 | RWE derived from multiple RWD sources is increasingly recognized as a valuable complement to clinical trials in HTA, particularly for addressing evidence gaps and reducing uncertainty in decision-making. Different RWD sources, including registries, clinical records, and unsupervised data, vary in quality and structure, requiring careful assessment of data provenance, transparency, and methodological rigor before integration. RWE can support HTA across the lifecycle by informing initial assessments, confirming or supplementing trial evidence, evaluating long-term outcomes, and assessing effectiveness in broader patient populations not represented in randomized trials. Integration of multiple data sources can enhance analytical value, but requires data linkage, interoperability, and standardized data models to ensure consistency and usability. Key challenges include data heterogeneity, variable quality, bias, privacy concerns, and lack of standardized frameworks, highlighting the need for robust methodologies, clear reporting standards, and coordinated efforts to enable reliable use of RWE in HTA processes. |
| 34 | Guerlich K et al., 2023 | European expert recommendations on clinical investigation and evaluation of high-risk MDs for children | 10.1111/apa.16919 | Stricter requirements introduced by the EU MDR have increased barriers to market access for high-risk paediatric MDs, leading to reduced availability of essential products due to high costs, long certification timelines, and limited commercial incentives. Clinical evidence generation in children is particularly challenging because of small and heterogeneous patient populations, ethical constraints, and difficulties in conducting adequately powered randomized controlled trials. Evidence requirements should follow a hierarchical approach, with randomized trials preferred when feasible, but alternative designs such as observational studies, case series, and registry-based data accepted when trials are not practical. Early involvement of paediatric experts, flexible regulatory pathways, and case-by-case assessment of evidence requirements are essential to ensure both safety and continued access to needed devices. Post-market surveillance through registries and long-term follow-up is critical to complement limited premarket evidence and monitor safety and effectiveness in real-world paediatric populations. |
| 35 | Hanna MG et al., 2024 | Recommendations for Performance Evaluation of Machine Learning in Pathology | 10.5858/arpa.2023-0042-CP | ML systems in pathology require structured performance evaluation due to the absence of established clinical guidelines and the potential impact on patient care. Evaluation must include verification and validation processes using RWD, ensuring that models are fit for their intended clinical use and can generalize to local deployment settings. A risk-based approach is essential, considering factors such as clinical impact, potential errors, workflow integration, and consequences of incorrect predictions. Key components of evaluation include data quality and representativeness, model training and testing characteristics, performance metrics, and compatibility between training data and real-world clinical data. Continuous monitoring, change management, and integration with human oversight are necessary to maintain safety, reliability, and effective use of ML-based decision support systems in clinical pathology practice. |
| 36 | Holmes DR et al., 2022 | Regulatory strategies for early device development and approval | 10.1002/ccd.30151 | Early MD development follows a structured but iterative process that begins with identifying unmet clinical needs and progresses through design, prototyping, and preclinical testing under controlled conditions. Regulatory pathways are initiated through IDE studies, which enable the collection of clinical safety and effectiveness data with close interaction between sponsors, investigators, and regulatory authorities. EFS allows initial clinical evaluation with small patient cohorts and flexible study designs, supporting iterative modifications of device design based on emerging clinical evidence. Close collaboration with regulators, use of staged evidence generation, and adaptive study approaches facilitate more efficient development while maintaining safety oversight. Additional regulatory pathways, such as breakthrough device designation, provide accelerated evaluation for technologies addressing serious conditions with limited alternatives, supporting timely innovation and approval. |
| 37 | Holmes DR et al., 2024 | The MD development ecosystem: Current regulatory state and challenges for future development: A review | 10.1016/j.carrev.2023.09.005 | MD development involves a complex ecosystem that spans from early innovation and design through clinical testing, regulatory approval, and integration into clinical practice. A risk-based regulatory framework is applied, with increasing evidence requirements from low-risk to high-risk devices, and clinical trials must be carefully designed to minimize bias and ensure clinically meaningful outcomes despite practical constraints such as limited blinding and smaller sample sizes. Programs such as Early Feasibility Studies and the Breakthrough Devices Program support earlier access to innovation by enabling flexible study designs, iterative development, and closer collaboration between sponsors and regulators. The total product life cycle approach emphasizes continuous evidence generation, combining premarket data with post-market surveillance using registries and RWD to monitor safety, effectiveness, and device performance over time. Key challenges include delays in study initiation, variability in data quality, limited diversity in clinical trials, and the need for better integration of RWE, requiring coordinated efforts, improved infrastructure, and stronger stakeholder collaboration. |
| 38 | Hu K et al., 2019 | Study on the Clinical Evaluation of Image-based Artificial Intelligence Aided Diagnosis Software Approved in the United States | 10.3969/j.issn.1671-7104.2019.05.019 | Image-based AI diagnostic software approved in the United States is primarily regulated through 510(k) and De Novo pathways, with clinical evaluation approaches tailored to device novelty, risk level, and intended use. Most products function as decision-support tools rather than autonomous systems, providing outputs such as alerts, classifications, or scores that assist clinicians rather than replace them. Clinical evaluation commonly relies on retrospective or prospective datasets annotated by multiple experts, with performance assessed using key metrics such as sensitivity, specificity, and area under the curve, often supported by confusion matrix analysis. Study designs incorporate additional considerations such as case balance, blinding, worst-case scenario analysis, and use of RWD to ensure robustness and clinical relevance of results. Secondary outcomes, including repeatability, localization accuracy, and workflow efficiency, are also used to demonstrate clinical utility and practical benefits in real-world settings. |
| 39 | Hulstaert F et al., 2023 | Gaps in the evidence underpinning high-risk MDs in Europe at market entry, and potential solutions | 10.1186/s13023-023-02801-7 | Clinical evidence supporting high-risk MDs at market entry in Europe is often limited, heterogeneous, and insufficient for robust assessment of clinical effectiveness. A minority of devices are supported by randomized controlled trials, while many rely on observational studies, surrogate outcomes, or evidence derived from equivalent devices, resulting in substantial uncertainty regarding real clinical benefit. Key evidence gaps include poorly defined target populations, lack of appropriate comparators, short follow-up periods, and limited reporting of patient-relevant outcomes such as quality of life and overall survival. Regulatory approval through CE marking focuses on safety and performance rather than demonstrated effectiveness, and clinical data submitted for approval are largely confidential, restricting transparency and independent evaluation. These limitations hinder evidence-based decision-making by healthcare payers and highlight the need for stronger requirements for comparative studies, improved data transparency, and alignment between regulatory and HTA evidence standards. |
| 40 | Jang KJ, 2021 | Computer-Aided Clinical Trials for MDs | N/A | RCTs remain the standard method for evaluating MDs, but they are resource-intensive, time-consuming, and often fail to provide sufficient or generalizable evidence, particularly for complex and heterogeneous patient populations. Computer-aided clinical trials introduce a framework that combines physiological modeling, simulation, and RWD to generate virtual patient cohorts and predict clinical outcomes before or alongside traditional trials. This approach can improve statistical power and reduce required sample sizes by incorporating simulated data while quantifying uncertainty associated with model assumptions and variability. The methodology enables early evaluation of device performance, supports optimization of device parameters, and helps identify potential risks or failure scenarios prior to large-scale clinical deployment. Integration of in-silico methods with conventional clinical trials offers a scalable, data-driven strategy to enhance efficiency, robustness, and decision-making in MD evaluation. |
| 41 | Jørgensen JT, 2019 | Regulatory requirements for companion diagnostics and drug-diagnostic codevelopment in the United States | 10.1016/B978-0-12-813539-6.00016-X | CDx are in vitro diagnostic devices that provide essential information for the safe and effective use of corresponding therapeutic products, including identifying suitable patients, predicting treatment response, and monitoring therapy. Due to their critical role in treatment decisions, most CDx assays are classified as high-risk (Class III) devices and require rigorous regulatory evaluation through premarket approval, including analytical and clinical validation. Drug–diagnostic codevelopment is a coordinated process in which the diagnostic test and therapeutic product are developed together, ensuring that the diagnostic is validated and available at the time of drug approval. Clinical trials for these products often rely on validated assays to select or stratify patients, and when final assays are not available, bridging studies are required to demonstrate equivalence between prototype and market-ready tests. Despite structured regulatory pathways, challenges remain in ensuring timely coordination, robust validation, and adaptation to increasingly complex biomarker-based and multi-parameter diagnostic technologies. |
| 42 | Kardjadj M, 2025 | Regulatory Approved Point-of-Care Diagnostics (FDA & Health Canada): A Comprehensive Framework for Analytical Validity, Clinical Validity, and Clinical Utility in MDs. | 10.1093/jalm/jfaf106 | The paper presents a comprehensive framework for the validation and regulatory approval of point-of-care diagnostic devices, structured around four key pillars: analytical validity, clinical validity, clinical utility, and regulatory alignment. Analytical validity focuses on ensuring accuracy, reproducibility, and reliability through metrics such as sensitivity, specificity, limits of detection, and agreement analyses, supported by standardized statistical methods. Clinical validity requires demonstration of performance in real-world settings through prospective studies, comparison with reference standards, and assessment across diverse populations and use environments. Clinical utility emphasizes the impact on patient outcomes and healthcare systems, including improvements in time to treatment, reductions in hospital stay, and cost-effectiveness supported by health economic analyses and patient-reported outcomes. The framework also highlights the importance of harmonizing regulatory requirements across jurisdictions, integrating post-market surveillance and RWE, and adapting validation approaches to emerging technologies such as AI, microfluidics, and connected devices to ensure safe, effective, and value-based implementation. |
| 43 | Kent S et al., 2021 | The use of nonrandomized evidence to estimate treatment effects in health technology assessment. | 10.2217/cer-2021-0108 | Nonrandomized studies are increasingly used in HTA to estimate treatment effects when RCTs are unavailable, infeasible, or insufficient, particularly for MDs, rare diseases, and expedited approvals. These studies can provide valuable insights into real-world effectiveness and broader patient populations, but they are inherently more susceptible to bias due to a lack of randomization, confounding, and variability in data quality. Methodological rigor is essential, including prospective study planning, appropriate selection of comparators, use of advanced analytical techniques to address confounding, and extensive sensitivity analyses to assess robustness of results. Transparent reporting, protocol registration, and formal assessment of risk of bias are critical to ensure credibility and reproducibility of findings. Despite improvements in methods and data availability, substantial uncertainty remains, requiring HTA bodies to critically appraise such evidence and implement strategies to manage uncertainty in decision-making. |
| 44 | Kesselmeier M et al., 2020 | Effect size estimates from umbrella designs: Handling patients with a positive test result for multiple biomarkers using random or pragmatic subtrial allocation | 10.1371/journal.pone.0237441 | Umbrella trial designs use a single screening platform to allocate patients to multiple biomarker-defined subtrials, improving efficiency compared to independent parallel trials by reducing the number of screened but unused patients. Patients who test positive for multiple biomarkers create allocation challenges, as they are eligible for more than one subtrial but can only be assigned to one, leading to their underrepresentation across subtrials. The study shows that umbrella designs can alter the distribution of biomarker-defined subgroups within subtrials, particularly reducing the proportion of patients with multiple positive biomarkers compared to traditional trial designs. These shifts in patient composition can influence estimated treatment effects when there is interaction between biomarker status and treatment response, potentially leading to biased or non-comparable results. Statistical approaches such as weighted regression combined with random allocation can partially adjust for these differences, but careful interpretation is required when using effect estimates from umbrella trials for decision-making or future research. |
| 45 | Kim DS and Song I, 2023 | A Review on the Post-Market Surveillance of MDs in the United States and its Implication: A Focus on RWD Using Unique Device Identification of MDs | 10.52937/hira.23.3.1.22 | PMS of MDs in the United States includes both passive systems, based on voluntary reporting of adverse events, and active systems, such as prospective studies and registries that systematically collect safety data. RWD play a central role in modern PMS, particularly through initiatives like the NEST, which integrates data from registries, electronic health records, and claims databases to detect safety signals. The introduction of the UDI system enables precise tracking of devices across their lifecycle, supporting linkage of device use with clinical outcomes and adverse events when integrated into health information systems. Despite these advances, challenges remain, including underreporting in passive systems, limited integration of UDI into routine clinical data, and difficulties in linking device data across systems. Strengthening PMS requires improved data integration, broader adoption of UDI in RWD sources, and coordinated systems that enable long-term monitoring of device safety and performance. |
| 46 | Laka M et al., 2024 | Evaluating clinical decision support software (CDSS): challenges for robust evidence generation. | 10.1017/S0266462324000059 | CDSS presents unique challenges for evidence generation because traditional MD evaluation frameworks do not adequately capture its dynamic, evolving nature and interaction with clinical environments. Current evaluation approaches often focus narrowly on technical performance or usability, failing to assess broader impacts on clinical workflows, user behaviour, and healthcare systems. Rapid software updates and evolving functionalities can change system performance and risk profiles over time, making one-time or static evaluations insufficient for ensuring safety and effectiveness. Evidence generation is further complicated by misalignment between fast-paced technological development and slower, resource-intensive evaluation methods such as randomized trials, which may become outdated before completion. Continuous, lifecycle-based evaluation using RWE and a socio-technical perspective is necessary to capture the full impact of CDSS and ensure safe and effective implementation in clinical practice. |
| 47 | Li H and Yue LQ, 2018 | Practical considerations in clinical strategy to support the development of injectable drug-device combination products for biologics | 10.1080/19420862.2017.1392424 | Development of injectable drug–device combination products for biologics is a complex, iterative process that integrates drug formulation, manufacturing, and delivery device design across the entire clinical development lifecycle. Manufacturing processes for biologics are highly sensitive, and even minor changes can affect product quality, safety, or efficacy, often requiring comparability assessments and, in some cases, additional clinical studies. A risk-based hierarchical approach is used to determine the extent of evidence needed, combining analytical, nonclinical, and clinical data depending on the nature and timing of changes. Clinical bridging studies, particularly pharmacokinetic/pharmacodynamic comparisons or efficacy trials, are typically required when significant changes occur at later stages of development. Patient-centric considerations, including usability, delivery method, and real-world administration, are essential components of development, influencing both regulatory approval and long-term treatment adherence. |
| 48 | Lord SJ, 2025 | Is this test fit-for-purpose? Principles and a checklist for evaluating the clinical performance of a test in the new era of in vitro diagnostic (IVD) regulation | 10.1080/10408363.2025.2453148 | The paper presents a structured checklist and set of principles for evaluating whether an in vitro diagnostic test is fit for its intended clinical purpose, emphasizing that clinical performance must be defined in relation to how the test will be used in practice. Clinical performance is framed as the ability of a test to correctly classify a target condition in a specific population, with key measures including sensitivity, specificity, predictive values, and other discrimination metrics that allow estimation of benefits and harms from correct or incorrect classification. The framework highlights the importance of clearly defining the intended purpose of the test, the target condition, the study population, and the role of the test within the clinical pathway, as these elements directly influence study design and interpretation of results. Different study designs are recommended depending on the test purpose, including cross-sectional studies for diagnosis, cohort studies for prognosis or monitoring, and randomized trials when assessing the prediction of treatment benefit. The checklist is intended to support development, evaluation, and regulatory approval of diagnostic tests by improving consistency, ensuring that evidence is aligned with clinical use, and enabling assessment of whether a test is likely to provide meaningful clinical benefit. |
| 49 | Lu NT et al., 2018 | Incorporating a companion test into the noninferiority design of MD trials | 10.1080/10543406.2018.1489403 | Noninferiority trials for MDs may lead to misleading conclusions when the investigational device appears non-inferior, but the active control performs below clinically acceptable levels. A dual-hypothesis approach is proposed, combining the standard noninferiority test with a companion test that evaluates whether the active control meets a predefined performance threshold. Study success is defined only when both the noninferiority hypothesis and the companion hypothesis are satisfied, ensuring that results reflect meaningful clinical performance. This approach addresses limitations of the constancy assumption, which may be violated due to changes in clinical practice, patient populations, or trial conditions. Incorporating the companion test into the design phase improves transparency, strengthens the validity of conclusions, and supports more reliable regulatory decision-making. |
| 50 | Lu N et al., 2019 | Good statistical practice in utilizing RWD in a comparative study for premarket evaluation of MDs | 10.1080/10543406.2019.1632880 | Observational comparative studies using RWD are increasingly applied in premarket evaluation of MDs when randomized trials are not feasible, but they introduce multiple sources of bias that must be carefully addressed. Bias can arise from differences in patient characteristics, study conduct, data quality, and unmeasured confounders, potentially compromising validity if not mitigated through appropriate design and analysis. Good statistical practice includes the use of propensity score methods to balance baseline covariates, combined with a two-stage design that separates study planning from outcome analysis to reduce bias and prevent data-driven decisions. High-quality RWD must demonstrate relevance, reliability, completeness, and comparability across data sources, with careful selection and validation of registries or datasets used as controls. Additional bias assessment, including evaluation of consistency across multiple data sources and use of independent statisticians, is necessary to ensure robustness, transparency, and regulatory acceptability of study results. |
| 51 | Marang-van de Mheen et al., 2024 | Decision framework to assess the performance of high-risk MDs | N/A | A structured decision framework was developed to assess the performance of high-risk MDs by integrating evidence from registries and other RWD sources across the product lifecycle. Analysis of European cardiovascular and orthopaedic registries revealed substantial heterogeneity and incomplete reporting of key methodological and quality indicators, limiting comparability and regulatory usability of registry data. Performance of the same devices varied across registries, indicating that evidence from a single data source may not be generalizable and that combining multiple registries can provide more robust assessments. A consensus-based minimum dataset was defined, including key elements on data quality, completeness, outcome definitions, and analysis, to support consistent evaluation of registry data in regulatory contexts. The final framework emphasizes relevance and reliability of data, incorporating factors such as data suitability, governance, quality, and analytical methods to guide assessment of safety, performance, and benefit–risk of MDs. |
| 52 | Marcus HJ et al., 2024 | The IDEAL framework for surgical robotics: development, comparative evaluation and long-term monitoring | 10.1038/s41591-023-02732-7 | The IDEAL framework provides a structured, lifecycle-based approach for evaluating surgical robotics, covering stages from preclinical development to long-term post-market monitoring. Early-stage evaluation emphasizes safety, feasibility, and iterative development, requiring transparent documentation of device modifications, use of technical and clinical metrics, and consideration of factors such as autonomy level, AI integration, and human–device interaction. Comparative evaluation in later stages requires well-designed prospective studies, including randomized trials when feasible, with clearly defined clinical, technical, and patient-reported outcomes, while accounting for learning curves, variability in surgical practice, and system-level impacts. The framework highlights the importance of multidisciplinary perspectives, including clinicians, patients, developers, and health systems, to address usability, ethics, cost-effectiveness, and acceptability of robotic technologies. Long-term monitoring relies on RWD and collaborative data collection systems to assess safety, effectiveness, and evolving performance of surgical robots after widespread adoption. |
| 53 | McDermott O and Kearney B, 2024 | A review of the literature on the new European MD Regulations requirements for increased clinical evaluation | 10.1108/IJPHM-07-2023-0060 | The EU MDR significantly strengthens clinical evaluation requirements, requiring more robust, high-quality, and continuously updated clinical evidence to demonstrate device safety, performance, and benefit–risk profile. Manufacturers face major challenges in determining what constitutes sufficient clinical evidence, particularly due to inconsistent expectations from notified bodies and limited guidance, leading to uncertainty and delays in regulatory approval. Stricter rules on demonstrating equivalence reduce reliance on existing devices, often necessitating costly and time-consuming clinical investigations, especially for high-risk and legacy devices. Increased regulatory scrutiny, including expert panel review and ongoing post-market data requirements, places additional burden on manufacturers to maintain comprehensive clinical evaluation reports throughout the device lifecycle. These challenges have led to potential market withdrawal of devices and delayed introduction of new technologies in the EU, raising concerns about reduced availability of MDs and impacts on patient access and outcomes. |
| 54 | McDermott O and Kearney B, 2024 | The value of using RWE as a source of clinical evidence in the European MD regulations: a mixed methods study | 10.1080/17434440.2023.2291454 | RWE is increasingly recognized as a valuable source of clinical evidence under the EU MDR, particularly for bridging gaps in clinical data for legacy devices and supporting post-market obligations. RWE provides a more accurate and representative assessment of device safety and performance by capturing data from real-world clinical use across larger and more heterogeneous patient populations. It can supplement or partially replace traditional clinical investigations, reducing costs and resource requirements while supporting benefit–risk evaluation and regulatory submissions. RWE also enables identification of opportunities for device improvement, expansion of intended use, and generation of long-term performance data through post-market clinical follow-up activities. Despite its potential, challenges remain related to data access, quality, infrastructure, and lack of clear regulatory guidance in the EU, limiting consistent adoption in clinical evaluation processes. |
| 55 | MD Coordination Group | Questions & Answers Regarding Performance Studies of In Vitro Diagnostic MDs under Regulation (EU) 2017/746 | N/A | Performance studies for IVDs under the EU IVDR are defined as investigations conducted to establish or confirm analytical and/or clinical performance, forming a core component of clinical evidence generation. Analytical performance studies assess the technical ability of a device to detect or measure analytes, while clinical performance studies evaluate the relationship between test results and clinical conditions in the intended population. Clinical performance evidence can be derived from multiple sources, including clinical studies, scientific literature, and routine diagnostic data, with studies required unless adequately justified otherwise. All performance studies must comply with general safety and performance requirements, ensure protection of study subjects, and generate reliable and scientifically valid data for regulatory evaluation. Additional regulatory requirements, including application or notification to competent authorities, depend on study characteristics such as invasiveness, use of companion diagnostics, and whether the device is used within or outside its intended purpose. |
| 56 | MedTech Europe and EFPIA | Determining the Path for Assessment of a Companion Diagnostic (CDx) under the In Vitro Diagnostic MDs Regulation | N/A | CDx under the IVDR are classified as high-risk Class C devices and require a conformity assessment process involving both a Notified Body and consultation with medicinal product authorities such as the EMA or national competent authorities. Regulatory pathways for CDx and their associated medicinal products remain largely independent, creating challenges in aligning development timelines, evidence requirements, and approval processes. Effective co-development requires early and coordinated interaction between stakeholders, including joint scientific advice and alignment on clinical development plans, biomarker validation, and evidentiary expectations. Clinical evidence generation for CDx may involve direct inclusion in pivotal drug trials, bridging studies, or comparative analyses between assays, with potential future use of RWD to support additional indications or claims. Key challenges include limited regulatory guidance, a lack of coordination between institutions, resource constraints such as insufficient NBs, and the need for clearer processes for joint review, conflict resolution, and lifecycle management of CDx. |
| 57 | MedTech Europe Clinical Evidence Working Group | Clinical Evidence Requirements under the EU IVDs Regulation | N/A | Clinical evidence under the IVDR is built on three core components: scientific validity, analytical performance, and clinical performance, which together demonstrate that a device is safe and effective for its intended purpose. The intended purpose defined by the manufacturer is central to evidence generation, as it determines the type, level, and scope of performance evaluation, clinical studies, and post-market activities required. Clinical evidence can be generated from multiple sources, including clinical studies, published literature, and routine diagnostic data, provided that data quality, relevance, and scientific rigor are ensured. Performance evaluation is a continuous, lifecycle-based process that integrates pre-market and post-market data, including post-market performance follow-up, to maintain and update evidence over time. The IVDR introduces stricter and more structured requirements compared to the previous directive, emphasizing transparency, methodological rigor, and a systematic approach to benefit–risk assessment and clinical evidence documentation. |
| 58 | Melvin T et al., 2024 | Research and analysis on regulatory framework and institutional and organization characteristics of EU competent authorities | N/A | EFS are small-scale clinical investigations conducted early in device development to evaluate initial safety, performance, and design concepts, typically before finalization of the device. In the EU, EFS are not governed by a dedicated regulatory program but are conducted within the broader framework of clinical investigations under the MD Regulation, resulting in limited specificity and guidance tailored to early-stage studies. Development activities are structured across stages before, during, and after EFS, with requirements to address key questions such as device readiness, study design, and justification based on risk–benefit considerations. The regulatory landscape relies on a combination of MDR requirements, ISO standards, and guidance documents, but these are generally designed for clinical investigations overall rather than EFS-specific needs, leading to gaps in practical implementation. Effective execution of EFS requires early and continuous dialogue with regulators, flexibility for iterative device modifications, and consideration of emerging technologies such as digital health, which introduce additional complexity in study design and evidence generation. |
| 59 | Min Y et al., 2019 | Clinical Trial Assessment Principles of National Class III MDs in China | 10.1111/os.12498 | Class III MDs in China are considered high-risk and therefore require rigorous clinical trials to demonstrate safety and effectiveness before approval. Clinical trial design relies heavily on the selection of appropriate assessment indices, divided into major and minor effectiveness endpoints and safety outcomes, with the primary index directly reflecting the intended function of the device. Most studies adopt randomized non-inferiority designs comparing the investigational device with an approved control, ensuring that performance falls within an acceptable predefined margin based on clinical practice and historical data. Robust statistical planning is essential, including justified sample size estimation, use of meta-analysis to define parameters, and analysis across multiple datasets (full analysis set, per protocol set, and safety set) to ensure reliability and consistency of results. Additional methodological requirements include multicentre study designs, defined inclusion and exclusion criteria, adequate follow-up periods, ethical approval, informed consent, and careful management of bias and missing data to ensure validity of conclusions. |
| 60 | Mooghali M et al., 2025 | Nonconcurrent Control Use in FDA Approval | 10.1001/jamanetworkopen.2025.6230 | More than half of FDA approvals for high-risk therapeutic MDs between 2019 and 2023 relied on analyses using nonconcurrent controls, such as historical controls, objective performance criteria, or performance goals, rather than concurrent comparator groups. Performance goals were the most commonly used approach, accounting for the majority of analyses, despite being based on less robust underlying evidence compared to other control types. Justification for using nonconcurrent controls was rarely reported, and clinically relevant details—such as comparability of patient populations or recency of control data—were often missing, limiting transparency of the evidence base. Available data used for these controls were frequently outdated, sometimes more than a decade old, raising concerns about their relevance to current clinical practice and technological standards. These limitations introduce uncertainty in assessing safety and effectiveness, particularly due to risks of bias, confounding, and inability to establish comparative effectiveness against contemporary standards of care. |
| 61 | Mühlbacher AC and Juhnke C, 2017 | Benefit Assessment for Examination and Treatment Methods with MDs of High Hisk: Trade-off between Patient Benefit, Evidence and Access | 10.1055/s-0043-112742 | Benefit assessment of high-risk MDs is structured as a three-phase process involving measurement of clinical effects, evaluation of benefits and harms, and decision-making on reimbursement based on aggregated overall benefit. Assessment requires comparative evaluation of patient-relevant outcomes, integrating both clinical endpoints and patient-reported outcomes to capture multidimensional patient benefit. RCTs remain the reference standard for demonstrating causal effects, but their application is limited by device-specific challenges such as rapid innovation cycles, learning curve effects, and difficulties in adapting study designs. Adaptive study designs and lifecycle-based evidence generation allow iterative data collection and modification of studies, supporting earlier access while progressively reducing uncertainty about safety and effectiveness. Decision-making incorporates trade-offs between evidence quality, patient benefit, and timely access, often supported by multi-criteria decision analysis and adaptive pathways that enable conditional reimbursement with ongoing evidence generation. |
| 62 | Naci H et al., 2020 | Generating comparative evidence on new drugs and devices before approval | 10.1016/S0140-6736(19)33178-2 | Comparative evidence on new MDs at the time of regulatory approval is limited, with many devices entering the market without robust data comparing them to existing alternatives. Clinical studies supporting approval are often non-randomized, single-group, or placebo-controlled, which restricts the ability to assess relative effectiveness and safety in real clinical settings. Expedited regulatory programs further contribute to this limitation by allowing approvals based on smaller studies, shorter follow-up periods, and surrogate endpoints rather than clinically meaningful outcomes. Post-marketing studies rarely resolve these evidence gaps, as they are frequently delayed, incomplete, or methodologically similar to pre-approval studies, leaving persistent uncertainty about comparative benefit. Strengthening evidence generation requires routine use of randomized trials with active comparators, improved transparency, and alignment of regulatory and reimbursement incentives to ensure that meaningful comparative data are available for decision-making. |
| 63 | O'Brien MT et al., 2022 | Scientific and Regulatory Policy Committee Points to Consider for MD Implant Site Evaluation in Nonclinical Studies | 10.1177/01926233221103202 | Nonclinical implantation studies are a critical component of MD development, focusing on evaluating local tissue responses and overall safety of implanted materials before clinical use. Implant site evaluation requires specialized macroscopic and microscopic pathology methods tailored to the specific device, implantation site, and study objectives, as standardized approaches are often insufficient due to the diversity of devices and biological responses. Study design is highly individualized and should involve multidisciplinary collaboration, including early input from experienced pathologists to ensure appropriate sampling, controls, endpoints, and interpretation of findings. Local tissue responses at the implant site are expected and must be interpreted in context, as they may reflect normal adaptive or functional processes rather than adverse effects, depending on device characteristics and intended use. Lack of standardized terminology and variability in methods and regulatory expectations increase complexity, requiring flexible, case-by-case approaches to ensure accurate, relevant, and scientifically valid safety assessment. |
| 64 | O'Driscoll F et al., 2024 | Clinical Simulation in the Regulation of Software as a MD: An eDelphi Study | 10.2196/56241 | Clinical simulation is proposed as a flexible and cost-effective method for generating evidence on SaMD, addressing limitations of traditional evaluation approaches such as randomized trials in rapidly evolving digital health contexts. An eDelphi study identified 43 consensus-based criteria organized into seven domains, including study design, population, simulation fidelity, software characteristics, and analysis, forming the SIROS framework for regulatory evaluation. High-quality simulation requires detailed reporting of the SaMD purpose, intended users, study design, potential biases, and strategies to mitigate them, along with a clear definition of outcome measures and analytical methods. Fidelity of simulation—such as realistic clinical scenarios, representative synthetic patient data, and alignment with intended use—is essential for generating valid and generalizable evidence. Continuous monitoring of software elements, including machine learning algorithms and updates, as well as transparent assessment of risks, unintended consequences, and robustness of findings, is critical for supporting regulatory decision-making. |
| 65 | O'Neill T et al., 2019 | ISPOR, the FDA, and the Evolving Regulatory Science of MD Products | 10.1016/j.jval.2019.03.020 | RWE is increasingly integrated into regulatory science for MDs, offering opportunities to complement or partially replace traditional clinical trial data across the entire product lifecycle. RWE derived from RWD sources such as electronic health records, registries, and patient-generated data can support premarket development, regulatory submissions, and post-market surveillance while potentially reducing the time and cost of evidence generation. Regulatory frameworks emphasize that such data must be fit-for-purpose, requiring high standards of relevance, reliability, data quality, and appropriate analytical methods to ensure valid conclusions. Challenges remain, including data heterogeneity, missing or poorly structured information, limited standardization, and difficulties in identifying specific devices or diagnostic tests within datasets. Collaboration among stakeholders and the development of standardized frameworks and infrastructure are essential to ensure rigorous, transparent, and effective use of RWE in regulatory decision-making. |
| 66 | Pane J et al., 2018 | EU postmarket surveillance plans for MDs | 10.1002/pds.4859 | The EU MDR introduces a structured PMS plan as a central element for continuous monitoring of device safety, performance, and benefit–risk balance throughout the product lifecycle. The PMS plan requires a proactive and systematic approach to collecting and analyzing data from multiple sources, including adverse events, literature, registries, user feedback, and real-world clinical use, rather than relying solely on passive reporting systems. Manufacturers must define clear processes for signal detection, trend analysis, risk evaluation, and implementation of corrective or preventive actions, supported by predefined indicators and thresholds for reassessment. The PMS plan integrates closely with PMCF, periodic safety update reports, and risk management documentation, ensuring continuous updating of clinical evidence and regulatory compliance. A modular structure, separating general PMS processes from device-specific activities, supports consistent documentation, regular review, and effective lifecycle management of MDs. |
| 67 | Pane J et al., 2020 | Blockchain technology applications to postmarket surveillance of MDs. | 10.1080/17434440.2020.1825073 | The increasing complexity and volume of PMS data for MDs require more advanced and proactive approaches to ensure timely detection of safety issues and efficient data exchange among stakeholders. Blockchain technology offers a decentralized, immutable, and secure framework that can improve PMS by enabling reliable data sharing, enhancing traceability through integration with Unique Device Identification systems, and supporting real-time access to safety information across the device lifecycle. It can address key limitations of current PMS systems, including fragmented data sources, lack of standardization, delayed signal detection, and risks related to data integrity and cybersecurity. Blockchain also facilitates improved monitoring of supply chains, helping to detect counterfeit devices and enabling faster regulatory actions through comprehensive tracking of device distribution and use. Despite these advantages, implementation challenges remain, including data privacy concerns, interoperability with existing systems, scalability, and the need for standardized frameworks and stakeholder collaboration. |
| 68 | Park SH and Han K, 2018 | Methodologic Guide for Evaluating Clinical Performance and Effect of Artificial Intelligence Technology for Medical Diagnosis and Prediction | 10.1148/radiol.2017171920 | Evaluation of AI models for medical diagnosis and prediction requires rigorous methodological approaches that go beyond technical performance and address clinical validity and real-world applicability. Model performance should be assessed through both discrimination (e.g., sensitivity, specificity, and area under the ROC curve) and calibration, as these represent distinct aspects of predictive accuracy and reliability. Use of external validation datasets that reflect the target clinical population is essential to avoid overestimation of performance caused by overfitting and spectrum bias, which commonly arise in high-dimensional AI models. Study design must account for factors such as disease prevalence, patient spectrum, and data collection methods, as these can significantly influence performance estimates and generalizability. Ultimate validation of AI tools requires evidence of impact on patient outcomes through clinical trials or well-designed observational studies, demonstrating clinical utility beyond algorithmic accuracy. |
| 69 | Park SH et al., 2021 | Key Principles of Clinical Validation, Device Approval, and Insurance Coverage Decisions of Artificial Intelligence | 10.3348/kjr.2021.0048 | AI algorithms in healthcare are evaluated using metrics such as sensitivity, specificity, Dice similarity coefficient, and ROC or FROC curves, which quantify discrimination accuracy depending on the task and output format. Calibration accuracy is necessary when algorithms provide probabilistic outputs, as predicted probabilities must correspond to actual disease prevalence. Performance commonly decreases in external datasets due to overfitting and limited generalizability caused by heterogeneity in patients, devices, and clinical settings. External validation using independent datasets, especially through diagnostic cohort studies, provides a more realistic assessment of clinical performance than internal validation or case-control designs. Clinical utility is demonstrated only when the use of AI leads to improved patient outcomes, while regulatory approval is primarily based on technical performance and does not confirm real-world clinical benefit. |
| 70 | Pazart L et al., 2021 | Threats and opportunities for the clinical investigation of high-risk MDs in the context of the new European regulations | 10.5220/0010382902740284 | Clinical investigation of high-risk MDs in Europe is limited by a low number and poor methodological quality of studies, with many approvals based on literature data rather than prospective trials. Evidence levels are often weak, with only a small proportion of studies reaching high-quality standards such as randomized controlled trials, while the majority provide moderate or low-level evidence. Regulatory guidance remains vague and heterogeneous across European countries, leading to inconsistent study designs and uncertainty for manufacturers developing clinical protocols. Conducting randomized trials for MDs is particularly challenging due to factors such as device evolution during studies, lack of blinding, small target populations, and operator dependency. New methodological approaches, including adaptive trial designs, pragmatic trials, in silico modelling, and the use of RWD, are increasingly proposed to improve the feasibility and quality of clinical evaluation under evolving regulatory requirements. |
| 71 | Pearson SD et al., 2023 | Institute for Clinical and Economic Review - Peterson Health Technology Institute value assessment framework for digital health technologies | 10.57264/cer-2023-0154 | Digital health technologies require a distinct value assessment framework due to their heterogeneity, rapid innovation, and evolving regulatory and reimbursement pathways. The proposed framework evaluates value across two primary domains, clinical impact and economic impact, while also incorporating contextual factors such as the technology’s role in care, privacy and security, and developer characteristics. Safety and effectiveness are assessed using a risk-based, tiered model in which evidence requirements vary according to the technology’s function and potential risk, with higher-risk applications generally requiring more robust evidence, including randomized trials or other appropriate study designs. User experience and health equity are included as key components influencing real-world adoption and effectiveness, emphasizing usability, accessibility, and impact across diverse populations. Economic evaluation primarily focuses on budget impact, while also considering broader system effects, with the expectation that digital health technologies demonstrate value through improved outcomes, enhanced access, and/or more efficient use of healthcare resources. |
| 72 | Pongiglione B et al., 2021 | Do existing RWD sources generate suitable evidence for the HTA of MDs in Europe? Mapping and critical appraisal | 10.1017/S0266462321000301 | RWD sources for MDs in Europe are numerous and heterogeneous, with substantial variation in availability, structure, and content across countries and technologies. Most data sources include clinical outcomes such as mortality, readmission, and complications, while economic data are less consistently reported and are often limited to general indicators like length of stay. Registries are the dominant data source for mature technologies, whereas observational studies are more common for newer or complex devices. Key limitations include restricted data access, lack of standardization of outcomes, insufficient detail on costs, and absence of appropriate comparators in many datasets. RWD has strong potential to support health technology assessment, but improvements in data quality, accessibility, standardization, and methodological consistency are required for reliable and comparative evidence generation. |
| 73 | Rademakers FE et al., 2025 | CORE-MD clinical risk score for regulatory evaluation of artificial intelligence-based MD software | 10.1038/s41746-025-01459-8 | The paper proposes a simple risk-based scoring system developed within the CORE–MD project to guide the level of clinical evidence required for regulatory evaluation of artificial intelligence-based MD software across its lifecycle. The score combines three key domains—valid clinical association, technical performance, and clinical performance—each reflecting different aspects of safety, effectiveness, and context of use, and assigns values that together determine the overall risk level of the device. Higher scores indicate greater potential risk to patients and therefore require more extensive pre-market clinical evaluation, while lower scores allow more limited pre-market evidence combined with stronger post-market data collection and monitoring. The framework emphasizes that evaluation should consider not only algorithm accuracy but also transparency, human oversight, clinical context, and potential impact on patient outcomes, including risks related to bias, data quality, and changes in performance over time. By linking risk level to proportionate evidence requirements and integrating both pre- and post-market phases, the approach aims to support consistent, transparent, and flexible regulatory decision-making for AI-based MDs. |
| 74 | Ren Y et al., 2020 | Exploration and practice of RWD studies on innovative medical products in Boao Lecheng: analysis based on Chinese first case of approved MD using domestic RWD | 10.7507/1672-2531.202007133 | The paper describes the development of a RWD research system in the Boao Lecheng pilot zone, based on the first MD in China approved using domestic RWE. It outlines a structured framework in which RWD are generated from routine clinical use of innovative devices across the full patient pathway, including treatment at the pilot site and follow-up in patients’ local healthcare settings, enabling comprehensive longitudinal data collection. The research system relies on key components such as clearly defined research questions, multidisciplinary teams, appropriate study designs (prospective, retrospective, or ambidirectional), and the establishment of high-quality patient registries as the core data infrastructure. Emphasis is placed on data quality, including completeness, traceability, and control of bias and confounding through appropriate statistical methods and standardized data management processes. This approach demonstrates how RWE can be systematically generated and used to support regulatory approval of MDs, while highlighting the importance of integrated data platforms, coordinated governance, and continuous methodological development to improve future applications. |
| 75 | Richards HS et al., 2024 | Examining the application of the IDEAL framework in the reporting and evaluation of innovative invasive procedures: secondary qualitative analysis of a systematic review | 10.1136/bmjopen-2023-079654 | Application of the IDEAL framework in studies of innovative invasive procedures is inconsistent, with most research concentrated in early stages (stage 1 and 2a) and predominantly using case series designs. Types of innovation vary widely, including entirely new procedures, modifications of single procedural steps, and introduction of new devices or robotic approaches, often applied in new clinical contexts. Terminology used to describe stages of innovation is heterogeneous, ambiguous, and rarely defined, with terms such as “new,” “novel,” or “modified” used interchangeably without clear criteria. Justification for the study design is primarily based on limited existing evidence and uncertainty regarding feasibility and safety outcomes, rather than standardized criteria for stage classification. Challenges in clearly defining and identifying stages of innovation limit consistent application of the framework and indicate the need for more objective approaches based on risk, uncertainty, and available evidence. |
| 76 | Schad F and Thronicke A, 2022 | Real World Evidence – Current Developments and Perspectives | 10.3390/ijerph191610159 | RWE is generated from routinely collected healthcare data, including electronic health records, claims data, registries, and patient-generated data from devices and home settings. Its role has expanded from post-approval evaluation to inclusion in regulatory decision-making and early drug assessment processes. Regulatory agencies such as the FDA and EMA have introduced multiple frameworks and guidelines to standardize the use, submission, and evaluation of RWD. RWE has contributed significantly to areas such as COVID-19 research, oncology, and rare diseases by enabling faster evidence generation and inclusion of broader patient populations. Key challenges include a lack of standardized definitions, data heterogeneity, limited transparency, and issues with data quality and interoperability, which require further methodological and regulatory development. |
| 77 | Schnell-Inderst P et al., 2024 | Report on study design recommendations in guidance documents for high-risk MDs | N/A | The report identified and analysed 30 regulatory guidance documents, 12 ISO standards, and additional documents from research consortia addressing clinical investigations of high-risk MDs. Guidance across jurisdictions consistently emphasizes the need for clinical investigations based on evaluation of existing evidence, with randomized controlled trials generally considered the most robust design for confirmatory studies. Recommendations cover key aspects of study design, including population selection, comparators, endpoints, and statistical methods, although the level of detail and methodological rigor varies substantially between regulators. Regulatory guidance from the United States provides the most comprehensive and systematic recommendations, while European guidance is more limited and often focused on reporting requirements rather than detailed study design. Important gaps include lack of standardized terminology for objective performance criteria, limited methodological guidance for deriving such criteria, and insufficient differentiation between study design approaches for established versus novel medical technologies. |
| 78 | Serrano-Aguilar P et al., 2021 | Postlaunch evidence-generation studies for MDs in Spain: the RedETS approach to integrate RWE into decision making. | 10.1017/S0266462321000295 | The RedETS Monitoring Studies program was developed to generate postlaunch RWE for MDs to support coverage and reimbursement decisions within the Spanish National Health System. These studies are prospective, observational, single-arm investigations designed to address uncertainties related to effectiveness, safety, costs, and real-world use of selected technologies under routine clinical conditions. The approach includes structured protocols, predefined eligibility criteria, standardized data collection systems, and involvement of multiple stakeholders, including clinicians, patients, health authorities, and industry representatives. Findings from these studies are used to inform policy decisions such as maintaining, modifying, or withdrawing technologies from public funding based on real-world performance and impact. Implementation of the program revealed challenges, including delays in study initiation, difficulties in patient recruitment and data collection, and variability in research infrastructure, highlighting the need for improved coordination, streamlined processes, and stronger collaboration at national and European levels. |
| 79 | Shimazawa R and Ikeda M, 2019 | Regulatory requirements for companion diagnostics – Japan | 10.1016/B978-0-12-813539-6.00019-5 | Regulation of companion diagnostics in Japan is based on a risk-based framework in which these tests are classified as high-risk IVDs requiring review and approval by the PMDA in conjunction with therapeutic products. Clinical trial design emphasizes early identification and validation of predictive biomarkers, including the inclusion of biomarker-negative patients to assess differential treatment effects and support evaluation of clinical utility. Prospectively designed randomized controlled trials are preferred for confirmatory evidence, although retrospective analyses may be accepted under strict methodological conditions when prospective trials are not feasible. A key regulatory feature is the requirement for coordinated drug–diagnostic codevelopment, with contemporaneous submission and validation of both the therapeutic product and the corresponding diagnostic test. Additional requirements include analytical and clinical validation of diagnostics, concordance studies when different tests are used, and consideration of population-specific genetic differences that may influence biomarker relevance and regulatory decisions. |
| 80 | Siontis GCM et al., 2024 | Quality and transparency of evidence for implantable cardiovascular MDs assessed by the CORE-MD consortium | 10.1093/eurheartj/ehad567 | The analysis included 71 high-risk cardiovascular MDs and identified 308 prospectively designed studies involving 97,886 patients, with 81% of studies being non-randomized and only 19% randomized clinical trials. The majority of evidence was generated after CE-mark approval, with no randomized clinical trials published before approval for any device, and nearly one-third of devices lacked any prospective clinical study. Study quality was limited by small sample sizes, short follow-up periods, low rates of protocol preregistration, and infrequent availability of publicly accessible protocols, while outcome adjudication was reported in less than half of the studies. Non-randomized designs predominated and were frequently associated with high or critical risk of bias, whereas randomized trials were larger and methodologically more robust but remained limited in number. The overall body of evidence was insufficient in both quantity and quality, indicating major gaps in transparency, methodological rigor, and timely generation of clinical data for high-risk cardiovascular devices. |
| 81 | Song C et al., 2024 | Statistical considerations for some issues in clinical bridging studies evaluating companion diagnostic devices | 10.1080/10543406.2023.2220398 | Clinical bridging studies are used when a clinical trial assay is employed for patient enrolment instead of the final companion diagnostic, requiring retesting of preserved samples to establish concordance and infer clinical effectiveness of the final test. Key statistical measures include positive and negative percent agreement between assays, which directly influence the estimation of clinical efficacy in the target population. Multiple methodological challenges arise, including missing data due to poor sample retention, variability introduced by local laboratory tests, pre-screening bias, and limited evaluability in low biomarker prevalence settings. Missing data and sample quality issues can substantially reduce reliability and introduce bias, requiring approaches such as multiple imputation and sensitivity analyses to support valid inference. Low biomarker positivity rates and differences in assay sensitivity can lead to high uncertainty in effectiveness estimates, emphasizing the need for careful study design, sufficient sample sizes, and strategies to ensure representativeness and robustness of bridging analyses. |
| 82 | Stüdeli T and Hochberg L, 2021 | Clinical Usability Studies – Clash of Cultures? Study Design Proposal from Lessons Learned | N/A | Clinical usability studies integrate methods from human factors engineering and clinical trials to evaluate both usability and clinical outcomes of MDs in real-world settings over extended periods of use. Traditional usability studies are typically conducted in simulated environments with small samples and qualitative focus, while clinical trials emphasize controlled, quantitative, and statistically driven methodologies, creating challenges when combining these approaches. The proposed framework includes an initial laboratory-based session, a longitudinal real-world usage phase with mixed data collection methods, and a final controlled assessment to capture both first-time and learned user interactions. Data collection combines qualitative and quantitative measures, including user interviews, usage data, clinical endpoints, and patient-reported outcomes, allowing analysis of relationships between usability, adherence, and clinical effectiveness. Clinical usability studies provide added value by capturing long-term behavior, real-world adoption, and user experience, but require careful design to manage biases, training effects, and interactions between researchers and participants that may influence outcomes. |
| 83 | Su G, 2025 | Could one strategy fit all? A comparison of regulatory guidance from China, Europe, and the USA on MD clinical evaluation throughout the total product lifecycle | 10.1080/17434440.2024.2448848 | Regulatory frameworks in China, Europe, and the United States share common principles for clinical evaluation of MDs across the total product lifecycle, but differ substantially in specific requirements and implementation. Clinical evaluation strategies are based on three main approaches: exemption from clinical data, use of existing clinical evidence through equivalence, and generation of new data via clinical trials, with selection depending on device risk level and novelty. Key differences exist in exemption criteria, equivalence requirements, access to technical data of comparator devices, and the need for and structure of clinical evaluation reports. The European framework imposes more stringent and detailed requirements, particularly for high-risk and implantable devices, including broader obligations for post-market clinical follow-up and continuous updating of clinical documentation. These differences result in variations in time, cost, and evidence generation processes, requiring tailored regulatory strategies rather than a single unified approach across jurisdictions. |
| 84 | Tan J et al., 2022 | Expert consensus on post-marketing risk monitoring technology for high-risk implantable passive MDs based on real world data | N/A | Post-marketing surveillance of high-risk implantable passive MDs relies on RWD to identify and evaluate safety signals and ensure continued safe and effective use. Risk monitoring incorporates both passive approaches based on spontaneous reporting systems and active approaches that use predefined data collection from sources such as electronic health records, registries, and insurance databases. A structured monitoring framework consists of three sequential stages: signal identification through database construction and data processing, signal detection using statistical and analytical methods, and signal confirmation through observational study designs to establish causal relationships. Key methodological challenges include data quality issues, missing information, reporting bias, and confounding, requiring advanced techniques such as natural language processing, propensity score methods, and multivariable regression analyses. Integration of multiple RWD sources, development of device-specific registries, and application of advanced analytical methods are essential to improve signal detection, reduce underreporting, and enhance the overall effectiveness of post-market risk monitoring systems. |
| 85 | Tarricone R et al., 2020 | Lifecycle evidence requirements for high-risk implantable MDs: a European perspective | 10.1080/17434440.2020.1825074 | Clinical evidence generation for high-risk implantable MDs follows a lifecycle approach consisting of four key stages: pre-clinical pre-market, clinical pre-market, post-market diffusion, and post-market obsolescence and replacement. Each stage requires different types of evidence, starting with laboratory and simulation studies, progressing to early feasibility and comparative clinical studies, and continuing with long-term RWD collection through registries and observational studies. RCTs are considered the preferred design for comparative effectiveness, but practical and ethical limitations often require alternative approaches such as single-arm studies, registry-based comparisons, or hybrid designs. Post-market evidence plays a critical role due to initial uncertainty at market entry, with registries and RWD enabling assessment of long-term safety, effectiveness, and variability across populations and clinical settings. Effective evidence generation requires early planning, integration of regulatory and HTA requirements, and continuous data collection across the entire product lifecycle to support decision-making, reimbursement, and timely adoption or replacement of technologies. |
| 86 | Tarricone R et al., 2023 | An accelerated access pathway for innovative high-risk MDs under the new European Union MDs and health technology assessment regulations? Analysis and recommendations | 10.1080/17434440.2023.2192868 | The paper examines clinical evidence generation for high-risk MDs within the framework of new EU MDR and HTA regulations and proposes an Accelerated Access Pathway to improve timely patient access. Evidence generation follows a lifecycle approach from pre-clinical testing and early clinical studies to post-market surveillance using RWD, registries, and observational studies. Randomized controlled trials are preferred for demonstrating effectiveness, but practical and ethical constraints often require alternative designs such as single-arm or registry-based studies. Increased uncertainty at the time of approval, particularly under accelerated pathways, necessitates mandatory post-market evidence generation, continuous monitoring, and the possibility of reassessment or withdrawal if benefits are not confirmed. The proposed pathway emphasizes early and continuous coordination between regulators, HTA bodies, and manufacturers to align evidence requirements and support faster, evidence-based decisions on approval, reimbursement, and clinical use. |
| 87 | Timbie JW et al., 2021 | Use of RWE for Regulatory Approval and Coverage of MDs: A Landscape Assessment | 10.1016/j.jval.2021.07.003 | RWE, derived from sources such as electronic health records, claims databases, and patient registries, is increasingly explored to support regulatory approval and coverage decisions for MDs, although it remains primarily complementary to traditional clinical trial evidence. Randomized controlled trials continue to be the main standard for premarket approval, while RWE is more commonly used to supplement clinical data, particularly for assessing long-term outcomes, rare adverse events, and real-world performance. Significant challenges limit the use of RWE, including difficulties in accessing and linking data sources, a lack of unique device identifiers, limited longitudinal follow-up, insufficient methodological expertise, and uncertainty about regulatory acceptance. Regulatory guidance has encouraged the use of RWE, but ambiguity and lack of concrete examples of successful applications reduce manufacturers’ willingness to invest in its development. Payers apply similar standards of rigor to RWE as to other evidence but remain cautious due to concerns about bias, data quality, and analytical methods, emphasizing the need for improved study design, clearer guidance, and stronger methodological frameworks to expand its role in decision-making. |
| 88 | Timbie JW et al., 2024 | Lessons on the use of RWD in MD research: findings from the National Evaluation System for Health Technology Test-Cases | 10.57264/cer-2024-0078 | The study synthesized findings from 18 NESTcc Test-Cases to evaluate the use of RWD for MD research across multiple clinical areas and regulatory use cases. Major challenges included difficulty identifying specific devices due to limited use of unique device identifiers, incomplete and inconsistent data capture in electronic health records, limited reliability of coding systems, and challenges in extracting detailed information from unstructured data. Measurement of long-term outcomes was often constrained by incomplete follow-up and patient attrition, while data sharing barriers and missing data further reduced the reliability of analyses. Successful strategies included linking multiple data sources, using supply chain and manufacturer data to identify device use, leveraging clinical registries, applying common data models, and involving multidisciplinary teams with clinical and data science expertise. The findings highlight that although RWD has strong potential to support regulatory decision-making, improvements in data standardization, linkage, and methodological approaches are necessary to ensure reliable and scalable evidence generation. |
| 89 | Vidal C et al., 2022 | Contribution of methodologies adapted to clinical trials focusing on high risk MDs | 10.5220/0009374503370343 | Clinical trials for high-risk MDs are complex, costly, and often difficult to conduct using traditional RCT designs due to small populations, short device life cycles, operator dependence, and challenges with blinding and comparators. Adaptive and alternative methodological approaches, including cluster trials, expertise-based trials, cross-over designs, and sequential or multi-arm multi-stage trials, can address these limitations by increasing flexibility, improving feasibility, and reducing required sample sizes and study duration. Adaptive trial designs allow modifications during the study based on interim analyses, such as early stopping, sample size reassessment, or adaptation of treatment allocation, enabling more efficient and responsive evaluation. Bayesian methods further enhance flexibility by incorporating prior information from previous studies, registries, or similar devices and updating evidence as new data are collected, supporting decision-making under uncertainty. Despite their advantages, these methodologies remain underutilized due to limited expertise, regulatory concerns, and the need for extensive planning and stakeholder involvement, highlighting the importance of early collaboration and methodological training to improve their adoption in MD research. |
| 90 | Weir CJ and Taylor RS, 2022 | Informed decision-making: Statistical methodology for surrogacy evaluation and its role in licensing and reimbursement assessments | 10.1002/pst.2219 | Surrogate endpoints are widely used to accelerate clinical evaluation and decision-making by providing earlier indications of treatment effects, but their validity depends on demonstrating a strong and reliable relationship with final patient-relevant outcomes. A broad range of statistical methods has been developed to evaluate surrogate validity, including hypothesis-testing approaches, causal inference frameworks, meta-analytic models, and information-theoretic methods, with increasing use of Bayesian approaches to incorporate prior evidence and quantify uncertainty. Despite methodological advances, challenges remain due to variability in the strength of association between surrogate and clinical outcomes, potential bias, and the risk that surrogate improvements do not translate into real clinical benefit. In regulatory and reimbursement contexts, surrogate endpoints are frequently used for licensing and health technology assessment decisions, but often without consistent application of formal validation frameworks, leading to uncertainty in estimating long-term effectiveness and cost-effectiveness. Improved decision-making requires systematic validation of surrogate endpoints, use of quantitative frameworks to assess uncertainty, and implementation of conditional approval models with continued evidence generation based on final clinical outcomes. |
| 91 | White NA et al., 2023 | Question-based development of high-risk MDs: A proposal for a structured design and review process | 10.1111/bcp.15685 | The paper proposes a structured framework for the clinical development of high-risk MDs based on a question-based approach adapted from pharmaceutical research, addressing the lack of detailed guidance under the European MD Regulation. The framework begins with defining a target product profile that specifies desired and minimally acceptable characteristics related to safety, performance, and clinical benefit, which then guides the formulation of key development questions. These questions are systematically linked to clinical and pre-clinical studies, enabling clear identification of evidence requirements, measurable endpoints, and decision points throughout development. The approach emphasizes early identification of uncertainties, iterative refinement of design based on emerging data, and use of go/no-go decisions to minimize development risks and avoid late-stage failures. Integration of this structured process with post-market surveillance ensures that remaining uncertainties are monitored over time, supporting continuous evaluation of benefit–risk balance and facilitating communication with regulators, clinicians, and other stakeholders. |
| 92 | Wilkinson B and Van Boxtel R, 2020 | The MD Regulation of the European Union Intensifies Focus on Clinical Benefits of Devices. | 10.1177/2168479019870732 | The EU MDR introduces a stronger focus on demonstrating clinical benefits of MDs, requiring that intended benefits be clearly defined, measurable, and supported by clinical evidence as part of the overall benefit–risk assessment. Under this framework, clinical evaluation must go beyond safety and technical performance to include patient-relevant outcomes such as improvements in health status, quality of life, or disease management. Randomized controlled trials are considered a robust source of evidence, but practical and ethical limitations often necessitate the use of alternative data sources, particularly RWE collected during the post-market phase. Post-market clinical follow-up plays a critical role in confirming, refining, and sometimes identifying new or unexpected clinical benefits, especially for devices not previously evaluated in rigorous premarket studies. Continuous collection and evaluation of RWD, along with user training and feedback, support a dynamic understanding of device performance and enable adaptation of clinical benefit claims throughout the device lifecycle. |
| 93 | Zebachi S et al., 2025 | Navigating the Real World: A Scoping Review of Structured Frameworks to Effectively Identify, Evaluate, and Select Real‐World Data Sources for Fit‐for‐Purpose Studies | 10.1002/cpt.3746 | The scoping review identified nine structured frameworks designed to support the identification, evaluation, and selection of RWD sources for generating evidence in regulatory and health technology assessment contexts. These frameworks assess multiple dimensions, including study design characteristics, data relevance and reliability, ethical considerations, and practical aspects such as feasibility, cost, and data access. While randomized controlled trials remain the standard for evidence generation, RWD provide complementary advantages such as larger sample sizes, longer follow-up, and improved generalizability, but also introduce challenges related to data quality, completeness, and standardization. The identified frameworks vary in scope and methodology, with some focusing on feasibility and study design, while others emphasize data quality assessment or structured decision-making through scoring systems. Despite their usefulness, none of the frameworks fully integrate both identification and evaluation of data sources, and there is no consensus on a single optimal approach, highlighting the need for harmonized methods and more comprehensive tools to support fit-for-purpose RWE generation. |
| 94 | Zhang B et al., 2022 | Design and analysis of crossover trials for investigating high-risk MDs: A review | 10.1016/j.conctc.2022.101004 | Crossover clinical trials are used in the evaluation of high-risk MDs as an alternative to traditional randomized controlled trials, allowing each participant to receive multiple interventions and serve as their own control, which reduces variability and sample size requirements. These designs are particularly suitable for chronic conditions and have been applied in regulatory submissions to demonstrate safety and effectiveness, although they remain relatively uncommon in practice. Key advantages include improved efficiency, ethical benefits due to exposure of all participants to the intervention, and enhanced statistical power, while major limitations involve potential carryover effects, longer study duration, and increased analytical complexity. Regulatory recommendations emphasize careful study design, including prespecification of statistical analysis, evaluation of period and carryover effects, and strategies to minimize bias and confounding. Despite these advantages, crossover trials are still underutilized in high-risk device research, and their use is appropriate only when treatment effects are reversible and the condition being studied is stable over time. |
| 95 | Zhao Y et al., 2023 | Advances in studies on post-market safety data sources and signal detection for MDs | 10.19803/j.1672-8629.20220562 | Post-market safety monitoring of MDs increasingly relies on diverse RWD sources, including spontaneous adverse event reporting systems, electronic health records, insurance claims databases, and clinical registries, which together provide broader and more representative information on device performance in routine clinical practice. Emerging data sources such as social media and device-generated data further expand the scope of surveillance, although they introduce challenges related to data quality, standardization, and interpretation. Signal detection methods commonly combine multiple approaches, including disproportionality analyses, pharmacoepidemiological study designs, sequential probability ratio testing, and machine learning techniques, to improve sensitivity and accuracy in identifying potential safety issues. Advanced data governance techniques, such as natural language processing and the use of unique device identifiers and common data models, enable integration and analysis of heterogeneous data sources, supporting more effective and scalable surveillance systems. Despite these advancements, limitations related to bias, incomplete data, and lack of standardized methodologies remain significant, highlighting the need for continued development of integrated, data-driven frameworks for timely and reliable detection of safety signals in post-market settings. |
| 96 | Zhao Y et al., 2023a | Applicability of patient registry research to regulation of MDs | 10.19803/j.1672-8629.20220654 | Patient registries represent an organized form of RWD collection using observational methods to systematically capture clinical outcomes in defined populations, and are increasingly applied in the regulation of high-risk MDs. They enable continuous, standardized data collection across the product lifecycle, supporting evaluation of safety, effectiveness, and benefit–risk balance in real-world settings, particularly for implantable devices requiring long-term follow-up. Registries provide important advantages over spontaneous reporting systems by improving data completeness, enabling estimation of event rates, and allowing comparison across patient groups and devices, while also supporting early detection of safety signals and assessment of rare adverse events. Evidence generated from registries has been used to inform major regulatory decisions, including product recalls, safety warnings, and reclassification of device risk, demonstrating their value in proactive post-market surveillance. Despite their benefits, challenges remain related to data standardization, resource requirements, and integration with other data sources, highlighting the need for coordinated frameworks and the incorporation of registries into comprehensive regulatory systems. |
| 97 | Ziegler A et al., 2021 | A Modular Approach to Combine Postmarket Clinical Follow-Up Studies and Postmarket Surveillance Studies | 10.1055/s-0041-1735165 | The study proposes a modular approach to integrate PMCF and PMS into a single clinical investigation plan, addressing increased regulatory requirements under the EU MDR. The approach enables simultaneous collection of data on safety, performance, and device function within a unified framework, reducing duplication of effort and improving efficiency compared to conducting separate studies. A flexible modular structure allows inclusion of different datasets, patient populations, devices, and study endpoints, with the possibility to adapt specific modules depending on clinical context, regulatory requirements, and resource availability. The developed template incorporates international standards and guidance, including ISO 14155 and SPIRIT recommendations, to ensure methodological rigor and regulatory compliance. This combined approach facilitates more comprehensive and cost-effective evidence generation across the device lifecycle, although adaptation may be required depending on jurisdictional requirements and study-specific conditions. |
| 98 | Zisis K et al., 2024 | RWD: a comprehensive literature review on the barriers, challenges, and opportunities associated with their inclusion in the health technology assessment process | 10.3389/jpps.2024.12302 | The review evaluates the use of RWD and RWE in HTA and shows that, although randomized controlled trials remain the primary source of evidence, RWD is increasingly used to complement clinical data, particularly for long-term outcomes, effectiveness in routine practice, and cost-effectiveness analyses. Acceptance and use of RWD vary across countries and organizations, with some stakeholders remaining cautious due to concerns about reliability, while others actively incorporate observational studies, registries, and pragmatic data into decision-making. Major barriers include limited availability of high-quality local data, lack of standardization, methodological challenges in non-randomized study design, data fragmentation, privacy concerns, and insufficient expertise among stakeholders. Despite these limitations, RWD provides important advantages such as improved understanding of treatment performance in real-world populations, inclusion of underrepresented groups, and support for reimbursement and policy decisions when randomized evidence is limited. Addressing these challenges through improved data infrastructure, standardized methodologies, stronger collaboration, and clearer guidance is essential to enable more consistent and effective integration of RWD into health technology assessment processes. |

RWD - Real-World Data, RWE - Real-World Evidence, IVDs - In Vitro Diagnostics, MDs - Medical Devices, CDSS - Clinical Decision Support Systems/Software, ISCTs - In Silico Clinical Trials, HTA - Health Technology Assessment, CIMDs - Connected Intelligent Medical Devices, RCTs - Randomized Controlled Trials, EFS - Early Feasibility Studies, EU MDR - European Union Medical Device Regulation, IDEAL(-D) - Idea, Development, Exploration, Assessment, Long-term study framework (for Devices), NEST - National Evaluation System for health Technology, ML - Machine Learning, IDE - Investigational Device Exemption, AI - Artificial Intelligence, CE - Conformité Européenne marking, CDx - Companion Diagnostics, PMS - Post-Market Surveillance, UDI - Unique Device Identifier, EU IVDR - European Union In Vitro Diagnostic Regulation, EMA - European Medicines Agency, NBs - Notified Bodies, FDA - Food and Drug Administration, SaMD - Software as a Medical Device, PMCF - Post-Market Clinical Follow-up, ROC - Receiver Operating Characteristic, FROC - Free-response Receiver Operating Characteristic
